# Drivers of menstrual material disposal and washing practices: A systematic review

**DOI:** 10.1371/journal.pone.0260472

**Published:** 2021-12-03

**Authors:** Hannah Jayne Robinson, Dani Jennifer Barrington

**Affiliations:** 1 University of Leeds, Leeds, West Yorkshire, United Kingdom; 2 The University of Western Australia, Crawley, Western Australia, Australia; Kyonggi University, REPUBLIC OF KOREA

## Abstract

**Background:**

Disposal and washing facilities and services for menstrual materials are often designed based upon technical specifications rather than an in-depth understanding of what drives peoples’ choices of practices.

**Objectives and data sources:**

This systematic review identified and summarised the main behavioural drivers pertaining to the choice of disposal and washing practices of menstrual materials through the thematic content analysis and study appraisal of 82 publications (80 studies) on menstrual health and hygiene published since 1999, reporting the outcomes of primary research across 26 countries.

**Results:**

Disposal and washing behaviours are primarily driven by the physical state of sanitation facilities; however, this is intrinsically linked to taboos surrounding and knowledge of menstruation.

**Implications:**

Using reasons given for disposal and washing practices by menstruators or those who know them well, or inferred by authors of the reviewed studies, we identify the key considerations needed to design facilities and services which best suit the desired behaviours of both planners and those who menstruate.

**Inclusivity:**

The term menstruators is used throughout to encompass all those mentioned in the studies reviewed (girls and women); although no studies explicitly stated including non-binary or transgender participants, this review uses inclusive language that represents the spectrum of genders that may experience menstruation.

**Registration:**

The review protocol is registered on PROSPERO: 42019140029.

## Introduction

Menstrual health and hygiene (MHH) are an integral part of public health, recognized by an increase in research on this topic in the past decade [e.g. 1,2], and the recent definition of menstrual health [3]. Within the MHH space there has been a lot of research into the provision of menstrual materials, and subsequent interventions that provide those who menstruate with both reusable and disposable materials [4–6]. However, there has been less research into what happens once these materials have been used; the full lifecycle of these materials has often not been documented. Understanding the full lifecycle of menstrual materials is especially important for those designing the infrastructure of water, sanitation and hygiene (WASH) programs, including, but not limited to, toilets, bathing facilities, washing and drying facilities, incinerators, and solid waste management services. Water, sanitation and hygiene facilities need to be technically and socially appropriate to allow people to change and dispose of menstrual materials safely for them, the associated infrastructural systems and the environment. Disposal choices directly affect the functioning of sanitation systems; if materials are discarded in toilets or pit latrines, they can create blockages which reduce functionality of a system [7]. Disposal and washing practices can also have adverse health effects on users, for example, if there are no spaces for drying reusable materials, it is possible that infections could manifest if materials are used before they are dried properly [8].

Currently, the drivers behind menstruators’ choice of disposal and washing practices are often not documented and rarely considered when WASH facilities are designed. For example, although it alludes to the need for ‘cultural considerations’ when designing facilities, the International Standard on Non-Sewered Sanitation requires technology developers to provide users with instructions on how to dispose of their menstrual materials so as to protect the functioning of the technology, but this does not necessarily consider the reasons why a user may choose to flush materials despite knowing that it may harm the infrastructure [9]. This views users through a deficit lens [10], it assumes that if only they knew better they would change their behaviour. But WASH behaviours are not always driven by possessing the appropriate ‘knowledge’, for example, people may have been taught that it is unsafe to practice open defecation but choose to do so for reasons of convenience, pride and mental well-being [11]. Such disconnects between ‘knowledge’ and ‘action’ are why the field of WASH behaviour change scholarship exists [12].

Two systematic reviews have been published compiling the methods of menstrual disposal used in low and middle income countries [13,14], but neither thoroughly investigates why users practice these behaviours. A recent critical review of ‘unflushable’ objects entering waterborne sewerage highlights the lack of research into the drivers behind user decisions to dispose of non-biodegradable materials in toilets around the world [15]. No reviews have been published on menstrual material washing practices.

To better design WASH systems that meet the needs of those who menstruate it is important to understand what disposal and washing practices are currently used, but more importantly, why: if WASH professionals can understand what drives washing and disposal methods in different contexts, they can design technically robust systems that those who menstruate want to use. To address this, we systematically investigated the extant peer-reviewed literature on menstrual disposal and washing practices so as to determine: 1) What drives the behaviours of those who menstruate when deciding on a method of disposal or washing of used menstrual products?; 2) Are there differences in behavioural drivers and practices between low, middle and high income economies?; 3) If MHH programming is to be socially, environmentally, economically and technically sustainable, how does it need to engage with the drivers of behaviour around disposal and washing?

## Method

The review protocol is registered on PROSPERO: 42019140029 (https://www.crd.york.ac.uk/prospero/display_record.php?RecordID=140029) and is reported according to PRISMA guidance [16 and S1 PRISMA Checklist].

### Search strategy

**Fig 1:**
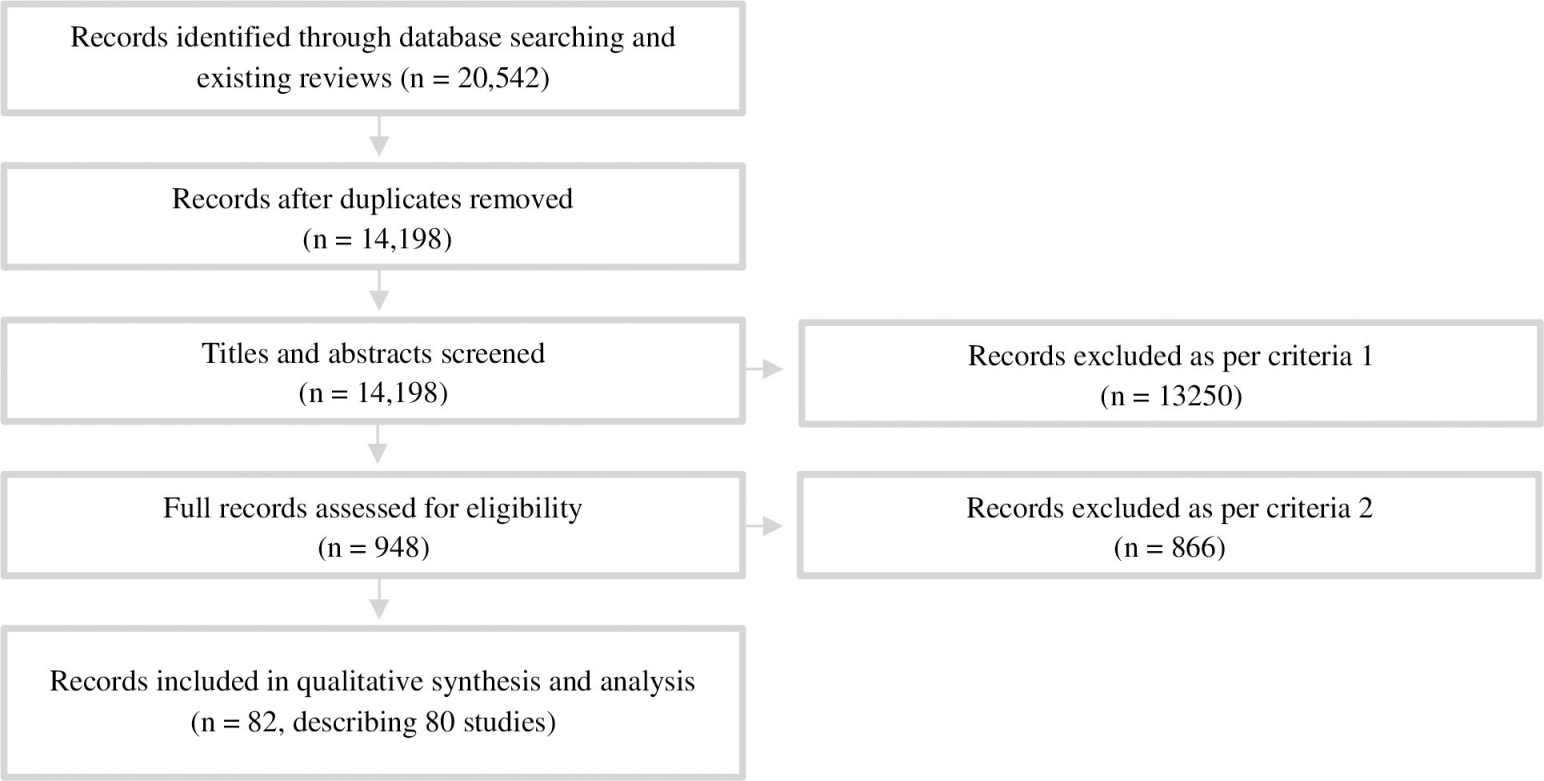
Inclusion and exclusion flowchart for systematic review [17]. Criteria 1: Primarily about menstruation or sexual and reproductive health; Criteria 2: Published post-1999 and discusses behaviours post-1999, in English or has an English translation available, discusses menstrual material disposal, washing, drying and/or reuse and gives reasons for these behaviours.

A systematic search of peer-reviewed literature was conducted according to the PRISMA guidelines [17] (Fig 1) and included documents published since 1999, to ensure that findings were relevant to current disposal and washing practices.

Topics to be searched for in the documents included: solid waste disposal; menstrual waste disposal; health effects regarding disposal and material usage, and factors affecting disposal routes (Table 1). Searching was undertaken in eight databases in June 2019 and updated in June 2021.

**Table 1:** Scopus search strategy.

Search 1: Menstruation	(menstru* or menarche).ab,kw,ti.
Search 2: Disposal or washing	(wash* OR dispos* OR dri* OR dry* OR recycl* OR reus* OR process* OR waste* or "used product*" OR threw* OR throw* OR rubbish OR garbage OR landfill OR bin OR hide OR bury* OR buried OR burn* OR hygien*).ab,kw,ti.
Search 3: Final	1 AND 2

ab = abstract, kw = keywords, ti = title.

This yielded the following results for initial screening: Scopus (8,367), Web of Science (5,050), EBSCO (consisting of CINAHL, GreenFILE, Social Work Abstracts, Child Development & Adolescent Studies) (1,681), MEDLINE (5,005) and Proquest Dissertations and theses (440). We also hand-searched the bibliographies of the two existing reviews on low/middle income country menstrual material disposal methods [13,14].

After removing duplicates, titles and/or abstracts of 14,198 publications were screened against Criteria 1 (primarily about menstruation or sexual and reproductive health), followed by the full-text screening of 948 publications against Criteria 2 (published post-1999 and discusses behaviours post-1999; in English or has an English translation available; discusses menstrual material disposal, washing, drying and/or reuse and gives reasons for these behaviours). This led to the inclusion of 82 publications (80 studies) (Table 2 provides a summary of the included studies, for full details of each see S1 Table). Grey literature was not searched as the themes which arose from the peer-reviewed publications reached saturation.

**Table 2:** Included studies.

Citation	Country	Economic status	Population	Sample Size
Abera, 2004 [18]	Ethiopia	Low	Girls in school*, school staff	863 questionnaires (female, grades 9–10 (≈ aged 14–16), across 8 schools), 4 focus group discussions (8 students in each), and key informant interviews with school authorities (number unspecified)
Ahmmed et al, 2021 [19]	Bangladesh	Lower-middle	Adolescent girls, Women, Birth Attendants and Medicine Vendors	89 married women (reproductive age), 42 adolescent girls (aged 14–18), 18 elderly women, 3 traditional birth attendants, 3 medicine vendors
Alda-Vidal and Browne, 2021 [20]	Malawi	Low	Women	40 Women (age unspecified), 13 sanitation workers and 15 external MHM actors
Alexander et al, 2014 [21]	Kenya	Lower-middle	School staff	62 Headteachers (age unspecified)
Asimah et al, 2017 [22]	Ghana	Lower-middle	Girls in school*, guardians	319 pupils (aged 10–19, with 229 females, 90 males across 15 schools), and 333 household heads (241 males, 92 females)
Averbach, et al, 2009 [23]	Zimbabwe	Lower-middle	Women	43 women (aged 18–45)
Behera et al, 2015 [24]	India	Lower-middle	Adolescent girls**	32 adolescent girls (female, aged 14–15)
Bhattacharjee, 2019 [25]	India	Lower-middle	Women and Adolescent Girls	84 Women and adolescent girls (aged 15–50, across 3 villages)
Caruso et al, 2017 [26]	India	Lower-middle	Women	69 women (for interviews, aged 18–75), and 46 women (for discussions, aged 18–70)
Caruso et al, 2014 [27]	Kenya	Lower-middle	Girls in school*, school staff	36 students (female, aged 11–17, across 3 primary schools for focus groups), 6 students (selected from the focus group discussions, for in-depth interviews), 2 teachers (for in-depth interviews)
Chakravarthy et al, 2019 [28] *(Paper uses 3 studies– 1 available report and 2 unpublished documents)*	India	Lower-middle	Women & girls, Government officials	Unspecified number of adolescent girls (aged 10–19) women (aged 20–49) and 20 government officials. Breakdown of participants not specified.
Chinyama et al, 2019 [29]	Zambia	Lower-middle	Girls in school*, school staff, guardians	64 students (aged 14–18, 48 female, 16 male, for 8 focus group discussions), 12 students (aged 14–18, female, for in-depth interviews), 7 teachers (for key informant interviews), (all across 6 schools), 7 guardians (for key informant interviews), and 11 leaders (both male and female (for key informant interviews)
Chothe et al, 2014 [30]	India	Lower-middle	Girls in school*	381 students (female, aged 9–13)
Connolly and Sommer, 2013 [31]	Cambodia	Lower-middle	Adolescent girls**, school staff, guardians	146 adolescent girls (female, aged 16–19, mix of in and out of school), and 15 parents/ teachers
Coswosk et al, 2019 [32]	Brazil	Upper-middle	Girls in school*, school staff	School principal and vice-principal, 39 students (female and male, aged 13–17)
Crankshaw et al, 2020 [33]	South Africa	Upper-middle	Girls in school, Boys in School, School staff, Mothers of Girls in School	505+ students (across 10 schools), 8 teachers, 9 mothers of students, (Breakdown of school students not specified)
Crichton et al, 2013 [34]	Kenya	Lower-middle	Adolescent girls**	87 students (aged 12–17), 69 women, 5 teachers, 1 nurse
Crofts and Fisher, 2011 and Crofts and Fisher, 2012 [35,36]	Uganda	Low	Girls in school*, school staff, business leaders	134 students (female, aged 13–20, for participatory activities and FDGs), 9 business leaders, 12 school staff
Daniels, 2016 [37]	Cambodia	Lower-middle	Adolescent girls**, adolescent boys, women, men, school staff	165 participants (for interviews), 181 participants (for focus group discussions), including girls, boys, mothers, fathers, and teachers. Breakdown of participants not specified.
Dhingra et al, 2009 [38]	India	Lower-middle	Adolescent girls**	200 girls (aged 13–15)
Dolan et al, 2014 [39]	Ghana	Lower-middle	Girls in school*, parents, school staff	99 girls (age unspecified, for interviews), 136 girls (age unspecified, including dropouts, for focus group discussions), 246 parents, 12 school staff (for key informant interviews), 156 school staff (for focus group discussions)
Ellis et al, 2016 [40]	Philippines	Lower-middle	Girls in school*	79 students (female, aged 11–18, across 3 schools in urban Manilla, and 10 rural schools)
Enoch at el, 2020 [41]	Ghana	Lower-middle	Adolescent girls	18 adolescent girls (aged 12–19, with visual, hearing or physical disabilities (6 girls for each disability))
Garikipati and Boudot, 2017 [42]	India	Lower-middle	Adolescent girls**and women	150 women and adolescent girls (aged 15–49, from 3 slum locations)
George and Leena, 2020 [43]	India	Lower-middle	Women	22 women (aged 25–49)
Girod et al, 2017 [44]	Kenya	Lower-middle	Girls in school*, school staff	51 students (approximately–number of students not explicitly stated, female, grades 6–8 (≈ aged 11–14), across 6 different primary schools) and 6 Headteachers
Gultie et al, 2014 [45]	Ethiopia	Low	Girls in school*	492 students (female, grades 9–12, aged 13–21+)
Habtegiorgis et al, 2021 [46]	Ethiopia	Low	Girls in School	536 students (female, aged 13–19, across 5 schools (3 public, 2 private, 457:79)
Hawkins et al, 2019 [47]	UK	High	Women	10 women (female, aged 18–30)
Hennegan et al, 2020 [48]	Uganda	Low	Women	35 Women (female, aged 18–35)
Hennegan and Sol, 2020 [49]	Bangladesh	Lower-middle	Girls in school	1359 students (female, aged 10–16, across 149 schools, (approximately 9 students per school))
Hennegan et al, 2017 [50]	Uganda	Lower-middle	Girls in school*	27 students (female, aged 12–17, across 8 schools)
Hennegan et al, 2016 [51]	Uganda	Lower-middle	Girls in school*	205 students (female, aged 10–19, across 8 schools)
Htun et al, 2021 [52]	Myanmar	Lower-middle	Adolescent girls	410 adolescent girls (aged 9–15, across 38 villages)
Jahan et al, 2020 [53]	Bangladesh	Lower-middle	Girls in school	Pre-intervention Period (PrIP):168 students and 17 school staff // Intervention Design Period (IDP): 139 students and 12 school staff // Post-intervention Period (PIP): 100 students and 20 school staff // 468 individuals, including students (419), teachers (21), and janitors (28) (All students aged 12–16)
Kambala et al, 2020 [54]	Malawi	Low	Women, girls in school, school staff, community leaders, community health workers, and MHM service providers	80 students (female, aged 10–18), 61 women, 12 school staff, 6 community leaders, 8 community health workers, and 9 MHM service providers
Karibu et al, 2019 [55]	Nigeria	Lower-middle	Adolescent girls**	492 adolescent girls (aged 10–19, covering both those in and out of school)
Kemigisha et al, 2020 [56]	Uganda	Low *(refugee settlement)*	Adolescent girls	28 adolescent girls (aged 13–19)
Kohler et al, 2019 [57]	India, Uganda	Lower-middle	Women, and men (inpatients and healthcare staff)	50 Indian participants and 40 Ugandan participants (across 4 hospitals, for workshops, interviewees selected from this sample). Both samples included inpatients and staff.
Kumbeni et al, 2020 [58]	Ghana	Lower-middle	Girls in school	730 students (female, aged 10–19, across 15 schools)
Lahme et al, 2018 [59]	Zambia	Lower-middle	Girls in school*	51 students (female, aged 13–20, across 3 schools)
MacRae et al, 2019 [60]	India	Lower-middle	Women	114 Women (across 12 communities– 39 unmarried women, 12 recently married women, 38 married women, 25 older women, age unspecified)
Mason et al, 2013 [61]	Kenya	Lower-middle	Girls in school*	120 Students (female, aged 14–16, cross 6 schools)
Maulingin-Gumbaketi et al, 2021 [62]	Papua New Guinea	Low-middle	Women	98 women (aged 13–45+, across 4 provinces)
McHenga et al, 2020 [63]	Malawi	Low	Girls in school and school staff	228 students (female, aged 11–22), 22 school staff (Head Teachers and Senior female teachers)
Miiro et al, 2018 [64]	Uganda	Lower-middle	Girls in school*, boys in school, school staff, Municipality officials, parents	562 Students (352 female and 210 male, aged 13–18, across 4 schools), 11 teachers, 2 municipality officials (Ministry of Education and the Ministry of Health), 10 parents
Mohamed et al, 2018 [65]	Fiji, Papua New Guinea, Solomon Islands	Lower-middle	Women & girls, men, school staff, community members (including vendors, employers, health workers, community leaders and vulnerable women)	54 girls in school (aged 13–26), 43 adolescent girls (aged 13–29), 118 women (aged 19–61), 51 men (aged 23–70), 8 school staff, and 34 community members
Mohammed and Larsen-Reindorf, 2020 and Mohammed et al, 2020 [66,67]	Ghana	Lower-middle	Girls in school, boys in school and 5 school staff	280 Students (250 female, aged 10–19, across 5 schools; 30 male, across 3 schools) and 5 head teachers
Mumtaz et al, 2019 [68]	Pakistan	Lower-middle	Girls in school, women, school staff, care providers, local religious leaders and a scholar	312 students (female, aged 16–19 years), 15 mothers, 11 female school teachers, 9 health care providers, 5 local religious leaders and 1 scholar
Muralidharan, 2019 [69]	India	Lower-middle	Women & girls	Up to 72 adolescent girls (aged 15–24), and their mothers (total of number of participants not stated)
Nalugya et al, 2020 [70]	Uganda	Low	Girls in school, parents, school staff	450 Students (baseline: 232 female and 218 male, aged 13–21, across 2 schools) 369 Students (endline: 188 female and 181 male, aged 13–21, across 2 schools), 10 parents, 10 teachers
Ndlovu and Bhala, 2016 [71]	Zimbabwe	Lower-middle	Women, NGOs, Public Sector, Religious institutions	40 women, 30 key informants (15 males and 5 females, including public sector departments, churches and NGOs)
Oche et al, 2012 [72]	Nigeria	Lower-middle	Adolescent girls**	122 adolescent girls (aged 15–20, across 4 schools)
Parker et al, 2014 [73]	Uganda	Lower-middle, and Displacement Camp (in and out of displacement camps)	Girls in school*, women, school staff, health workers	Up to 240 students (aged 9–20, across 14 schools), 8 senior/head teachers, 9 health workers, up to 75 women (across 4 villages), up to 450 women (across 13 IDP settings) (Total of number of participants not stated)
Rajagopal and Mathur, 2017 [74]	India	Lower-middle	Adolescent girls**	270 adolescent girls (130 school-going, 140 non-school-going, aged 10–20, across 5 schools)
Rajaraman et al, 2013 [75]	India	Lower-middle	Women	48 women (age unspecified)
Ramathuba, 2015 [76]	South Africa	Upper-middle	Girls in school*	273 students (female, aged 14–19, across 6 schools)
Rastogi et al, 2019 [77]	India	Lower-middle	Girls in school*, parents, school staff	187 students (female, aged 13–15, across 4 schools), parents and school staff. Total of number of participants not stated.
Rheinländer et al, 2019 [78]	Ghana	Lower-middle	Girls in school*, school staff	33 students (female, aged 14–23, across 2 schools), 4 school staff (female)
Rizvi and Ali, 2016 [79]	Pakistan	Lower-middle	Adolescent girls**	20 adolescent girls (aged 13–19, non-school-going)
Roxburgh et al, 2020 [80]	Malawi	Low	Women and university staff	31 women (aged 19–60+) and 2 university staff
Schmitt et al, 2017 [81]	Lebanon, Myanmar	Displacement Camp	Women & girls, humanitarian staff	117 women (aged 19–49), 71 adolescent girls (aged 14–18, 32 for focus group discussions and 39 for participatory mapping), 17 emergency response staff
Schmitt et al, 2021 [82]	Bangladesh	Low-middle *(refugee settlement)*	Women & girls, humanitarian response staff	47 Adolescent girls and women (aged 15–35), 19 humanitarian response staff
Scorgie et al, 2016 [83]	South Africa	Upper-middle	Women	21 women (aged 18–35, 17 of these completed the photovoice segment, then 7 of these then completed interviews)
Shah et al, 2019 [84]	Gambia	Low	Girls in school*, mothers, school staff	470 students (427 female—aged 11–21, 43 male–aged 15–21), 3 school staff, 5 mothers
Sheoran et al, 2020 [85]	India	Lower-middle	Women & girls	800 Women & girls (aged 14–49)
Sivakami et al, 2019 [86]	India	Lower-Middle	Girls in school*	2564 students (female, aged 12+ (average age 14), across 43 schools)
Sommer et al, 2015 [87]	Cambodia, Ghana, Ethiopia (This study also draws from a previous study from Tanzania–Sommer, 2009, for comparison purposes, this study is detailed below)	Low and Lower-Middle	Adolescent girls**, school staff, parents, health staff	≈ 450 adolescent girls (aged 16–19, both in and out of school, across the 3 countries), school staff, parents, health staff (total of number of participants not stated)
Sommer, 2009 [88]	Tanzania	Lower-Middle	Adolescent girls**	≈ 140 adolescent girls (aged 16–19) (Total of number of participants not stated)
Sommer et al, 2020 [89]	USA	High *(homeless women)*	Women, government staff, shelter staff	22 women (aged 16–62), 3 government staff and 12 shelter staf
Tamiru et al, 2015 [90]	Ethiopia, South Sudan, Tanzania, Uganda, Zimbabwe	Low and Lower-Middle	Girls in school*, boys in school, school staff, community members	Total of number of participants not stated (students aged 11+)
Tegegne and Sisay, 2014 [91]	Ethiopia	Low	Adolescent girls**, school Staff	At least 595 students (female, aged 10–19), 5 adolescent girls (who had dropped out of school), 4 teachers (all female). Total of number of participants not stated.
Trinies et al, 2015 [92]	Mali	Low	Girls in school*, school Staff	26 students (female, aged 12–17, across 8 schools), 14 school staff (4 female, 10 male, across 8 schools).
Umeora and Egwuatu, 2008 [93]	Nigeria	Lower-middle	Women	1692 women (female, aged 17–56).
Visaria and Mishra, 2017 [94]	India	Lower-middle	Adolescent girls**	585 adolescent girls (aged 12–19, split across the experiment area (406) and a control group (179), spanning rural and urban communities). Total number of participants not explicitly stated.
Wardell and Czerwinski, 2001 [95]	USA	High	Women	33 women (aged 22–27, on active duty or reserve forces for the military)
WaterAid Nepal, 2009 [96]	Nepal	Low	Girls in school*	204 students (female, aged 12–20, across 4 schools)
Wilbur et al, 2021 [97]	Nepal	Lower-middle	Women and carers	20 women and girls (aged 15–24) and 13 carers
Wilson et al, 2014 [98]	Kenya	Lower-middle	Girls in School*	302 students (female, unknown age, across 10 schools)
Yeasmin et al, 2017 [99]	Bangladesh	Lower-middle	Women, men, waste emptiers	43 women, 25 men, 14 children, 5 faecal sludge emptying operators, 4 waste bin emptiers

*‘Girls in school’ refers to studies that were specifically conducted in a school environment

**‘adolescent girls’ refers to participants either not in education, or studies that were set outside the school environment.

### Quality appraisal

Included studies were assessed on their level of trustworthiness and relevance by adapting the method of Rees et al [100] to be applicable to both quantitative and qualitative MHH studies. The assessment of trustworthiness was dependant on the sampling, data collection and analysis, and interpretation of data; this yielded high, medium, and low ratings. Relevance related to the proportion of the paper dedicated to menstrual material disposal or washing, whether confidentiality had been assured and appropriate consent obtained, and who gave the reasons for behaviours; this yielded high, medium, and low ratings (for full details of the quality appraisal of each study, see S2 Table). We considered studies which took a social constructivist approach to understand drivers of menstrual disposal and washing, evidenced by the experiences of menstruators, to be of higher relevance than those which presented less direct evidence and tended along positivist epistemological lines. Considering the trustworthiness and relevance of studies allowed us to weight themes more heavily where they were results of robust research, particularly where the reasons for disposal and washing behaviours were given by those who menstruate themselves.

### Thematic content analysis

After reviewing the full texts of each publication, deductive and inductive coding was undertaken using NVivo 12 [101]:

1. Examples of practices were deductively coded as “intentional disposal” or “washing drying or reuse”.
2. Axial coding was conducted to identify the drivers behind each practice (see Table 3 for codebook and coding frequency). This led to an understanding of the influence of physical and social drivers on menstrual material disposal and washing practices and how these must be considered by those planning WASH facilities.
3. Publications were also classified demographically in order to understand the study context (Tables 2 and S1).

**Table 3:** Codebook definitions and coding frequency.

	Code	Definition		Studies
**Practise (Deductive Codes)**	Intentional Disposal	Menstruators chose to engage in a certain disposal technique (e.g. throwing into a latrine, field, jungle, canal, or bin; flushing down a toilet, burying them; wrapping them in newspaper, plastic, or paper; or leaving them on the toilet floor)		56 (70%)
	Washing, drying or reuse	Washing between uses to reuse materials; washing blood of materials for religious/cultural reasons before disposing; drying in the sun (on roofs/washing lines); or drying inside homes (open-air drying and hiding whilst drying)		47 (59%)
**Reason / Behavioural Driver (Inductive Codes)**	State of Available Facilities *(40 Studies)*	Physical Infrastructure	Does the sanitation facility meet desired physical sanitation needs?	52 (65%)
		Social Perceptions	Does the sanitation facility meet desired social needs?	42 (53%)
	Knowledge *(11 Studies)*	Lack of knowledge	Menstruators have not been taught how to dispose / wash / dry materials	14 (18%)
	Menstrual Taboos and Social Stigma *(36 studies)*	Cultural Beliefs	General beliefs discouraging / encouraging certain methods of disposal	28 (35%)
		Embarrassment and Worry	Unpleasant emotions related to doing something considered by others to be wrong or shameful	35 (44%)
		Fear	Unpleasant emotion caused by the threat of danger, pain or other harmful consequences	13 (16%)

A conceptual model was developed to understand the breadth of reasoning behind disposal and washing practices.

## Results

Publications detailed studies in low income economies (17 studies), lower middle income economies (58 studies), upper-middle income economies (5 studies), high income economies (3 studies), and displacement camps (3 studies) (as defined by World Bank in 2020 [102]) (Tables 2 and S1). There was a skew towards girls’ (<18 years old) experiences over women’s experiences (58 instances vs. 29 instances). There were no studies which detailed the experiences of those who identify as trans-men or gender non-binary.

49 studies were rated as high trustworthiness, 30 medium trustworthiness and 1 low trustworthiness. High trustworthiness papers were characterised by having more than 50 participants, a clear analysis description, and supportive quotes that were clearly distinguishable between participants. 26 studies were considered of high relevance to this review, 46 medium relevance and 8 low relevance. High relevance studies tended to have a high proportion of the paper discussing findings relevant to disposal and washing of menstrual materials, evidence of behavioural drivers as stated by menstruators themselves, and consent and confidentiality measures stated clearly. Lower relevance studies only briefly mentioned disposal and washing behaviours and/or presented the reasons for behaviours mostly from author inferences, rather than given by menstruators themselves.

In 29 studies, reasons for disposal and washing practise were given by menstruators alone, in 23 reasons were given solely by authors, and in 28 there was a mixture of authors’ reasoning and menstruators’ reasoning. The reasons given for disposal and washing practices for each study are detailed in S1 Table and a summary of the frequency of reasons is detailed in Table 3. Illustrative examples of the drivers of disposal and washing behaviours are provided in S3 Table.

### Reasoning behind behaviour

When investigating the reasoning behind menstruators’ choice to use certain disposal and washing practices, the predominant factor was the availability of appropriate WASH facilities (where ‘appropriateness’ was defined by users). Of the 80 studies, 56 mentioned that the reason for the disposal or washing practice directly related to the state of the facilities used to manage menstruation, 52 the physical needs of the individual, and 42 the social perceptions of the facility according to individuals. There were 13 instances of menstruators stating lack of knowledge as a reason that affected their behaviour, as they stated they were unsure what was supposed to be done after menstrual materials had been used. Menstrual stigma and taboos were stated in 55 papers as a reason influencing disposal and washing practices.

Where the physical state of WASH infrastructure was mentioned, it related to the quantity of available and physically functional toilets/latrines [22,26,28,32,33,37,40,44,46,58,63,73,75,76,78,81,86,87,89,91], the design of toilets/latrines [32,37,40,44,48,51,57,63,64,68,71,73,75,81,83,87,90,97], the quality and availability of running water in and around toilets or latrines [18,20,31,33,37,39,40,45,46,48,49,51,53,54,56–60,62,63,66–68,71,73,77,87,88,91,92,94–96], the availability of soap for washing [20,33,49,53,54,56–58,60,61,63,64,66,67,73,77,90,91] and the availability of a physically functional disposal mechanism and/or service for used material [18,28,31–37,42,44–48,52,53,57,58,60,62,63,66–68,71,76,77,82,83,86,90,91,95–97].

Social perceptions of appropriate infrastructure were driven by the presence or absence of a private/safe space for managing menstruation [28,34,36,37,39,40,44–46,48,51–54,57,60,63,64,66–69,71,74,76,80,81,83,87,89–92,96], the cleanliness and maintenance of the facility [25–28,31,33,36,37,40,43,44,46,48,53,57,63,66–68,71,74,77,78,87–90,92,96], the time they had available to change, wash or dispose of materials [25,28,33,53,86] and the availability of gender-segregated toilets / latrines [53,58,63,81,87,91,92].

In 14 of the studies menstruators explicitly stated that they had been given no, or limited, advice regarding how to dispose of menstrual materials [21,24,28,30,37,45,64,74,78,81,83]. However, menstruators also stated they chose their method of disposal to limit environmental harm [55], or not cause detrimental harm to infrastructure systems [83] (e.g., disposable pads blocking flush systems).

55 studies indicated that the choice of disposal or washing behaviour was driven by menstrual taboos and social stigma. For example, menstruators that used reusable materials often dried them discreetly to hide them from others, often drying their washed materials inside, sometimes in a hidden corner [38,50,51,55,73,74,84,91–94], under their clothes [60,69,74,76,81], hidden under or within other drying items [25,36,49,60,65,73,96], or generally inside out of view [48,52,58,68]. There were instances of drying menstrual materials outside [46,49,50,55,56,65,73,98] but typically in cases where menstruators believed that there was adequate separation of homes so that neighbours could not see. When using disposable materials, many menstruators wrapped their used menstrual materials in newspaper or polythene bags before disposal, so as to obscure their waste [24,25,32,40,41,43,47,54,60,63,69,74,77,79,81,83,85,97,99]. In addition, some cultures discouraged the use of open disposal, such as bins, due to fears of witchcraft and infertility [19,20,29,30,36,41,42,53,57,60,68,71,72,82,83,87,90,92,93,99], or due to beliefs it is a sin to throw (especially unwashed) materials into a bin [79].

## Discussion

There are three main drivers of menstrual disposal and washing behaviour, and they can be independent or influenced by one another. They can be standalone, for example, there may be no bin within the WASH facility so a menstruator cannot dispose of materials into a receptacle, the menstruator may not have been taught how to dispose of used materials so does not know what to do, or the menstruator may have been taught that they must not incinerate materials lest evil spirits negatively affect their health. However, the reasons for disposal and washing menstrual materials can also be multi-faceted. In some instances all three combine to influence behaviour: for example, a menstruator may not have been taught how to dispose of materials, feel uncomfortable openly discussing disposal options due to menstrual stigma and may not know what facilities are needed or available, or how to change facilities or practices to make them more appropriate.

### Understanding the drivers

The state of WASH facilities was the predominant driver in 57 studies. This included the physical needs and social perceptions of menstruators, but often also incorporated menstrual taboos and social stigma, for example, just because facilities existed, and were technically ‘appropriate’, did not mean they were used. Crofts and Fisher noted that incinerators had been constructed in five of the 18 schools where interviews took place, so there was a physically functioning menstrual disposal option present, however the actual usage was low [36]. The incinerators had been built on the opposite side of the school to the toilets, so students did not want to be seen entering the incinerator building, and even when disposal buckets were provided in toilets, there was no management in place to take waste from toilets to the incinerator [36]. So, although there were physically appropriate disposal facilities, due to stigma around being seen holding or disposing of used materials, the disposal facility was not used. The theme of menstruators not wanting to be seen disposing or washing their used materials was driven by two emotions: being embarrassed and/or worried (35 studies), or fearing that they would be seen, and what would happen if they were seen (13 studies).

Embarrassment and worry were direct results of being seen disposing or washing menstrual materials, and often lead to hiding of materials, and secretive behaviours [19,20,25–28,33–35,37,40,44,47–49,53,58,60,62,65,68,73,74,76–81,83–85,92,97,99]. This worry was usually related to having ones’ menstrual status exposed (meaning those around the menstruating individual are aware that they are currently experiencing their period). Embarrassment drove disposal and washing behaviours and methods that favoured privacy. This behaviour can be harmful, as if menstruators hide materials when drying them (e.g. under beds or other clothes), they may not be properly dried, and it is possible to contract infections and skin irritation. Menstruators were often aware of the dangers of incomplete drying of reusable products [23,36,54,56,60,71], but felt they have no other alternative due to the stigma surrounding exposing menstrual status, an example of knowledge existing but stigma more strongly influencing a behaviour.

Beyond embarrassment, fear of being seen disposing or washing was a recurring theme [34,35,40,59,61,68,69,78,80,81,83,87,91]. This related to favouring methods of disposal or washing that were deemed secretive or hidden in order to limit their menstrual status being exposed [34,47,60,68,78,91]. Within this theme, menstruators noted they wanted to hide their used materials so that animals, specifically dogs, couldn’t find their materials and expose their status [20,41,60,70,82,83,87]. They especially wanted to hide their status from males, specifically fathers and fathers-in-law [40,47,60,61,68,80]. This was in part due to fears of unwanted sexual advance [35], abuse and violence [19,69,81] and being forced to leave school at menarche so as to get married [59,68]. Other fears to use disposal methods stemmed from young menstruators being afraid that having a period was a “punishment from God,” so hiding materials so that their parents would not find out [35], or that if seen, they would risk “infertility” or “being cursed” [87].

Cultural knowledge also influenced the choices that drove disposal and washing behaviours. For example, incinerators are often used for menstrual waste [35], and although they may be technically appropriate, in some cultures there are negative connotations surrounding the burning of menstrual blood [84] or instances where menstrual blood is kept to be used for ritual purposes [22,93]. These feelings were highlighted in Karibu et al’s Nigerian study where “38.0% stated that they chose what they considered to be the best disposal method to ensure protection from metaphysical forces…[and]… 2.4% said they chose their method to avoid evil people” [55].

### Biased menstrual choices

When examining the language used in the papers, it became apparent that there is a bias in the way some disposal and washing methods, as well as menstrual materials themselves, were written about by the authors, most of whom are WASH researchers or practitioners. There has been a tendency to write about material use or disposal and washing behaviours with a view that some are superior to others, even where there is limited health or technical evidence to support this. Bias was highlighted in statements such as ‘*only* x % of interviewees use sanitary pads’ (emphasis added, [42,90,91,94]). Although sometimes the bias was used in ways to highlight unhygienic practices, for example “only a few used any kind of antiseptic soap or liquid [to wash their pads]” [24] it still singled out and shamed individuals rather than considering the myriad of factors contributing to their behaviours and the suitability of their washing and disposal practices to their personal circumstances. A similar bias occurs in Community Led Total Sanitation and some sanitation marketing programmes, where people are identified, and often shamed for their behaviours, regardless of their ability to change a situation, whether that be due to insufficient funds, lack of access to different options, or personal behavioural and cultural choices [103,104].

It must be understood that there are many reasons for choosing specific menstrual materials and disposal and washing behaviours, and that writing in a style that judges choices may vilify individuals, reinforce harmful taboos, and not succeed in changing behaviours to those which may improve the wellbeing of those who menstruate. WASH practitioners who read such studies may develop their own biases against behaviours which are in fact appropriate to local contexts and low risk to menstruators’ health. For example, there has been a frequent bias in the WASH literature and programming against the use of ‘cloths’ or ‘rags’, often conflated as one. However, this does not consider that a cleaned and dried reusable pad or cloth may be just as hygienic and clean as a disposable pad, whilst a dirty rag poses obvious hygiene risks. In Chakravarty et al’s paper, this infiltrated bias had directly affected material use, with one menstruator stating, “here we use only pads, we now [after MHH programming] realise how unhygienic it is to use cloth” [28]. There are several factors that contribute to menstrual material choice including, but not limited to, the preferences of and options for disposal and washing available to those who menstruate.

### Implications for WASH programming

When programming for MHH and the associated WASH facilities, WASH professionals must consider not just preferences for menstrual material choice, technologies, and disposal and washing practices, but also the physical and social perceptions of menstruators, the availability and form of knowledge in the cultural context, and the menstrual taboos and social stigma that continue to impact on those who menstruate across the globe. The focus of MHH programming must be to improve the physical, mental, and social well-being of menstruators within their own contexts (the very definition of menstrual health [3]), not to promote specific WASH facilities or technologies preferred by implementers; considering the drivers of menstrual behaviour throughout the lifecycle of materials will assist in improved experiences for menstruators.

### Limitations

This was a global review of published data. We originally aimed to highlight and understand the disposal and washing practices of those who menstruate around the world, with no geographical or economic limitations. However, of the 80 studies included in this review, only three were from high-income economies and another five were from upper-middle income economies. This was surprising given the media coverage in recent years surrounding the ‘fatbergs’ negatively impacting the functionality of sewers in cities in higher income countries [105]. Such fatbergs are attributed largely to the flushing of menstrual materials and wet wipes. Water utilities have put out calls to stop such behaviour [106], but, similar to the way users are often encouraged to change their menstrual material disposal behaviours in LMICs through the imparting of knowledge, these campaigns tend to assume that people flush these items because they do not know how harmful the practice is. Very limited research has investigated the behavioural drivers of menstrual material disposal in higher income economies.

Thus, although this study set out to reflect worldwide practices, due to the lack of data from higher income economies, this study is not representative of global practices. There were parallels within the small amount of high/upper-middle income economy data that showed similar themes and reasoning behind drivers of behaviour to lower-middle and low income economices, but due to the low proportion of these papers, we cannot determine with certainty whether these behaviours are universal. We join with Alda-Vidal et al. [15] in calling for further social science research on the clear gap of higher income economy data, and suggest it will be of particular interest in areas where fatbergs are impacting on WASH systems.

## Conclusion

This review demonstrates the complex nature of washing and disposal behaviours related to menstruation. Behaviours are often not solely reliant on one factor, but several interrelated considerations. It is the first review that has aimed at understanding why people choose to engage in various menstrual material disposal and washing practices.

WASH professionals and other implementers of menstrual disposal and washing facilities and services need to ensure that disposal and washing options are appropriate for their context. Even when facilities are installed and accessible, if considered inappropriate they will not be used [36,57,71]. In addition, educational policy needs to allow for the teaching of menstruation in a scientific, judgement-free zone, where those who menstruate feel comfortable to learn without the fear of embarrassment. Many young menstruators, and teachers in some instances, were highlighted as not having sufficient knowledge about menstruation and menstrual management, or were biased to certain materials and practices, with a specific emphasis on missing disposal information [21,24,30]. By creating a safe space to facilitate discussion, young menstruators will be able to learn more about this taboo topic.

Menstrual material disposal and washing is an area that is poorly understood globally, and as the first systematic review compiling these behaviours, this paper begins to provide clarity in an under-researched area. By exploring the drivers of disposal and washing behaviours, we demonstrate the interfaces between facilities, knowledge and taboos d. It is clearly important to integrate these aspects into the planning and provision of infrastructure systems that interface with MHH in order to provide accessible and appropriate facilities for all.

## Supporting information

S1 Checklist PRISMA 2009 checklist. (DOCX)

S1 Table Characteristics of studies included in the systematic review. (DOCX)

S2 Table Quality appraisal of included studies. (DOCX)

S3 Table Illustrative examples showing drivers of behaviours. (DOCX)

## References

[1] Sommer M, Sahin M. Advancing the global agenda for menstrual hygiene management for schoolgirls.Advancing the global agenda for menstrual hygiene management for schoolgirls 2013;103(9):1556–9. doi: 10.2105/AJPH.2013.301374 23865645 PMC3780686

[2] Hennegan J, Montgomery P. Do menstrual hygiene management interventions improve education and psychosocial outcomes for women and girls in low and middle income countries? A systematic review.PLoS One 2016;11(2). doi: 10.1371/journal.pone.0146985 26862750 PMC4749306

[3] Hennegan J, Winkler IT, Bobel C, Keiser D, Hampton J, Larsson G, et al. Menstrual health: a definition for policy, practice, and research.Menstrual health: a definition for policy, practice, and research 2021;29(1):1911618. doi: 10.1080/26410397.2021.1911618 33910492 PMC8098749

[4] Shah SP, Nair R, Shah PP, Modi DK, Desai SA, Desai L. Improving quality of life with new menstrual hygiene practices among adolescent tribal girls in rural Gujarat, India.Reprod Health Matters 2013;21(41):205–13. doi: 10.1016/S0968-8080(13)41691-9 23684203

[5] APS Group Scotland. Access to Sanitary Products Aberdeen Pilot: Evaluation Report. Edinburgh, UK: The Scottish Government; 2018.

[6] Kansiime C, Hytti L, Nalugya R, Nakuya K, Namirembe P, Nakalema S, et al. Menstrual health intervention and school attendance in Uganda (MENISCUS-2): a pilot intervention study.BMJ Open 2020;10(2):e031182. doi: 10.1136/bmjopen-2019-031182 32024786 PMC7044877

[7] Mattsson J, Hedström A, Ashley RM, Viklander M. Impacts and managerial implications for sewer systems due to recent changes to inputs in domestic wastewater–A review.J Environ Manage 2015;161:188–97. doi: 10.1016/j.jenvman.2015.06.043 26182992

[8] Sumpter C, Torondel B. A Systematic Review of the Health and Social Effects of Menstrual Hygiene Management.PLoS One 2013;8(4). doi: 10.1371/journal.pone.0062004 23637945 PMC3637379

[9] International Standards Organisation. Non-sewered sanitation systems—Prefabricated integrated treatment units—General safety and performance requirements for design and testing. ISO 30500:2018. Switzerland: Geneva.: International Standards Organisation,; 2018.

[10] Wynne B. Knowledges in Context. Sci Technol Human Values 1991;16(1):111–21.

[11] Coffey D, Gupta A, Hathi P, Khurana N, Spears D, Srivastav N, et al. Revealed preference for open defecation.Econ Polit Wkly 2014;49(38):43–55.

[12] Curtis VA, Danquah LO, Aunger RV. Planned, motivated and habitual hygiene behaviour: an eleven country review.Health Educ Res 2009;24(4):655–73. doi: 10.1093/her/cyp002 19286894 PMC2706491

[13] Elledge MF, Muralidharan A, Parker A, Ravndal KT, Siddiqui M, Toolaram AP, et al. Menstrual Hygiene Management and Waste Disposal in Low and Middle Income Countries—A Review of the Literature.Menstrual Hygiene Management and Waste Disposal in Low and Middle Income Countries—A Review of the Literature 2018;15(11):1–16. doi: 10.3390/ijerph15112562 30445767 PMC6266558

[14] Kaur R, Kaur K, Kaur R. Menstrual Hygiene, Management, and Waste Disposal: Practices and Challenges Faced by Girls/Women of Developing Countries.J Environ Public Health 2018;2018. doi: 10.1155/2018/1730964 29675047 PMC5838436

[15] Alda‐Vidal C, Browne A, Hooloha C. Unflushables”: Establishing a global agenda for action on everyday practices associated with sewer blockages, water quality, and plastic pollution.Unflushables”: Establishing a global agenda for action on everyday practices associated with sewer blockages, water quality, and plastic pollution. 2020;7(7):e1452.

[16] Moher D, Liberati A, Tetzlaff J, Altman DG, The Prisma Group. Preferred Reporting Items for Systematic Reviews and Meta-Analyses: The PRISMA Statement.PLoS Med 2009;6(7):e1000097. doi: 10.1371/journal.pmed.1000097 19621072 PMC2707599

[17] Preferred Reporting Items for Systematic Reviews and Meta-Analyses. PRISMA 2009 Checklist 2009 [1 september 2019]. Available from: http://www.prisma-statement.org/PRISMAStatement/Checklist.aspx.

[18] Menarche Abera Y., Menstruation related Problems and Practices among Adolescent High School Girls in Addis Ababa, 2003/04: Addis Ababa University; 2004.

[19] Ahmmed F, Chowdhury MS, Helal SM. Sexual and reproductive health experiences of adolescent girls and women in marginalised communities in Bangladesh.Cult Health Sex 2021:1–16. doi: 10.1080/13691058.2021.1909749 33941046

[20] Alda-Vidal C, Browne AL. Absorbents, practices, and infrastructures: Changing socio-material landscapes of menstrual waste in Lilongwe, Malawi.Soc Cult Geogr 2021:1–21. doi: 10.1080/14649365.2021.1901974

[21] Alexander KT, Oduor C, Nyothach E, Laserson KF, Amek N, Eleveld A, et al. Water, sanitation and hygiene conditions in kenyan rural schools: Are schools meeting the needs of menstruating girls? Water (Basel) 2014;6(5):1453–66. doi: 10.3390/w6051453

[22] Asimah SA, Diabene PY, Wellington SNL. Menstrual hygiene management in Ghana: understanding the socio-cultural, economic, political factors, challenges and opportunities. Local action with international cooperation to improve and sustain water, sanitation and hygiene (WASH) services; Loughborough, UK. Loughborough: Loughborough University; 2017.

[23] Averbach S, Sahin-Hodoglugil N, Musara P, Chipato T, van der Straten A. Duet® for menstrual protection: a feasibility study in Zimbabwe.Contraception 2009;79(6):463–8. doi: 10.1016/j.contraception.2008.12.002 19442783

[24] Behera D, Sivakami M, Behera MR. Menarche and Menstruation in Rural Adolescent Girls in Maharashtra, India.Menarche and Menstruation in Rural Adolescent Girls in Maharashtra, India 2015;17(4):510–9. doi: 10.1177/0972063415612581 . Language: English. Entry Date: 20180117. Revision Date: 20180118. Publication Type: Article.

[25] Bhattacharjee M. Menstrual Hygiene Management During Emergencies: A Study of Challenges Faced by Women and Adolescent Girls Living in Flood-prone Districts in Assam.Indian J Gend Stud 2019;26(1–2):96–107. doi: 10.1177/0971521518811172

[26] Caruso BA, Clasen TF, Hadley C, Yount KM, Haardörfer R, Rout M, et al. Understanding and defining sanitation insecurity: Women’s gendered experiences of urination, defecation and menstruation in rural Odisha, India.BMJ Glob Health 2017;2(4). doi: 10.1136/bmjgh-2017-000414 29071131 PMC5640070

[27] Caruso BA, Dreibelbis R, Ogutu EA, Rheingans R. If you build it will they come? Factors influencing rural primary pupils’ urination and defecation practices at school in western Kenya.J Water Sanit Hyg Dev 2014;4(4):642–53. doi: 10.2166/washdev.2014.028

[28] Chakravarthy V, Rajagopal S, Joshi B. Does Menstrual Hygiene Management in Urban Slums Need a Different Lens? Challenges Faced by Women and Girls in Jaipur and Delhi.Indian J Gend Stud 2019;26(1–2):138–59. doi: 10.1177/0971521518811174

[29] Chinyama J, Chipungu J, Rudd C, Mwale M, Verstraete L, Sikamo C, et al. Menstrual hygiene management in rural schools of Zambia: A descriptive study of knowledge, experiences and challenges faced by schoolgirls.BMC Public Health 2019;19(1). doi: 10.1186/s12889-018-6360-2 30611223 PMC6321718

[30] Chothe V, Khubchandani J, Seabert D, Asalkar M, Rakshe S, Firke A, et al. Students’ Perceptions and Doubts About Menstruation in Developing Countries: A Case Study From India.Health Educ Res 2014;15(3):319–26. doi: 10.1177/1524839914525175 24618653

[31] Connolly S, Sommer M. Cambodian girls’ recommendations for facilitating menstrual hygiene management in school.J Water Sanit Hyg Dev 2013;3(4):612–22. doi: 10.2166/washdev.2013.168

[32] Coswosk ÉD, Neves-Silva P, Modena CM, Heller L. Having a toilet is not enough: The limitations in fulfilling the human rights to water and sanitation in a municipal school in Bahia, Brazil.BMC Public Health 2019;19(1). doi: 10.1186/s12889-019-6469-y 30704435 PMC6357509

[33] Crankshaw TL, Strauss M, Gumede B. Menstrual health management and schooling experience amongst female learners in Gauteng, South Africa: a mixed method study. Reprod Health 2020;17(1):1–15. doi: 10.1186/s12978-019-0847-x . Language: English. Entry Date: 20200420. Revision Date: 20200429. Publication Type: Article. 32293481 PMC7158143

[34] Crichton J, Okal J, Kabiru CW, Zulu EM. Emotional and Psychosocial Aspects of Menstrual Poverty in Resource-Poor Settings: A Qualitative Study of the Experiences of Adolescent Girls in an Informal Settlement in Nairobi.Health Care Women Int 2013;34(10):891–916. doi: 10.1080/07399332.2012.740112 23570366

[35] Crofts T J., Fisher J, editors. Schoolgirls’ experiences of managing menstrual hygiene in Uganda. 2011 35th WEDC International Conference—The Future of Water, Sanitation and Hygiene in Low-Income Countries: Innovation, Adaptation and Engagement in a Changing World; 2011; Loughborough.

[36] Crofts T, Fisher J. Menstrual hygiene in Ugandan schools: An investigation of low-cost sanitary pads. J Water Sanit Hyg Dev 2012;2(1):50–8. doi: 10.2166/washdev.2012.067

[37] Daniels GJ. Investigating Fear, Shyness, And Discomfort Related To Menstrual Hygiene Management In Rural Cambodia: Yale University; 2016.

[38] Dhingra R, Kumar A, Kour M. Knowledge and practices related to menstruation among Tribal (Gujjar) adolescent girls.Studies on Ethno-Medicine 2009;3(1):43–8. doi: 10.1080/09735070.2009.11886336

[39] Dolan CS, Ryus CR, Dopson S, Montgomery P, Scott L. A blind spot in girls’ education: Menarche and its webs of exclusion in Ghana.J Int Dev 2014;26(5):643–57. doi: 10.1002/jid.2917

[40] Ellis A, Haver J, Villasenor J, Parawan A, Venkatesh M, Freeman MC, et al. WASH challenges to girls’ menstrual hygiene management in Metro Manila, Masbate, and South Central Mindanao, Philippines.Waterlines 2016;35(3):306–23. doi: 10.3362/1756-3488.2016.022

[41] Enoch A, Nadutey A, Afful BF, Anokye R. Menstrual Hygiene Management: Challenges and Coping Strategies for Adolescents with Disabilities in the Kumasi Metro of Ghana. Disability, CBR & Inclusive Development 2020;31(2):77–91. doi: 10.47985/dcidj.364 Language: English. Entry Date: 20201126. Revision Date: . Publication Type: Article.20201126

[42] Garikipati S, Boudot C. To Pad or Not to Pad: Towards Better Sanitary Care for Women in Indian Slums.J Int Dev 2017;29(1):32–51. doi: 10.1002/jid.3266

[43] George AM, Leena KC. Experiences of the Women Using Menstrual Cup on Free Will—A Qualitative Inquiry.Online J Health Allied Sci 2020;19(3):1–4. . Language: English. Entry Date: 20210202. Revision Date: 20210203. Publication Type: Article.

[44] Girod C, Ellis A, Andes KL, Freeman MC, Caruso BA. Physical, Social, and Political Inequities Constraining Girls’ Menstrual Management at Schools in Informal Settlements of Nairobi, Kenya.J Urban Health 2017;94(6):835–46. doi: 10.1007/s11524-017-0189-3 28875308 PMC5722726

[45] Gultie T, Hailu D, Workineh Y. Age of menarche and knowledge about menstrual hygiene management among adolescent school girls in amhara province, Ethiopia: Implication to health care workers & school teachers.PLoS One 2014;9(9). doi: 10.1371/journal.pone.0108644 25268708 PMC4182550

[46] Habtegiorgis Y, Sisay T, Kloos H, Malede A, Yalew M, Arefaynie M, et al. Menstrual hygiene practices among high school girls in urban areas in Northeastern Ethiopia: A neglected issue in water, sanitation, and hygiene research.PLoS One 2021;16(6):e0248825. doi: 10.1371/journal.pone.0248825 34106948 PMC8189485

[47] Hawkins A, Sharpe R, Spence K, Holmes N. Inappropriate flushing of menstrual sanitary products.Proceedings of the Institution of Civil Engineers-Water Management 2019;172(4):163–9. doi: 10.1680/jwama.17.00050 WOS:000475712900001.

[48] Hennegan J, Kibira SPS, Exum NG, Schwab KJ, Makumbi FE, Bukenya J. ’I do what a woman should do’: a grounded theory study of women’s menstrual experiences at work in Mukono District, Uganda. BMJ Glob Health 2020;5(11). doi: 10.1136/bmjgh-2020-003433 33219001 PMC7682193

[49] Hennegan J, Sol L. Confidence to manage menstruation at home and at school: findings from a cross-sectional survey of schoolgirls in rural Bangladesh.Cult Health Sex 2020;22(2):146–65. doi: 10.1080/13691058.2019.1580768 30931818

[50] Hennegan J, Dolan C, Steinfield L, Montgomery P. A qualitative understanding of the effects of reusable sanitary pads and puberty education: Implications for future research and practice.Reprod Health 2017;14(1). doi: 10.1186/s12978-017-0339-9 28655302 PMC5488479

[51] Hennegan J, Dolan C, Wu M, Scott L, Montgomery P. Measuring the prevalence and impact of poor menstrual hygiene management: A quantitative survey of schoolgirls in rural Uganda.BMJ Open 2016;6(12). doi: 10.1136/bmjopen-2016-012596 28039290 PMC5223625

[52] Htun NN, Laosee O, Rattanapan C. Factors that influence menstrual hygiene management in adolescent girls in Mudon Township, Mon State, Myanmar.J Health Sci Med Res JHSMR 2021;39(3):207–17. doi: 10.31584/jhsmr.2021778

[53] Jahan F, Nuruzzaman M, Sultana F, Mahfuz MT, Rahman M, Akhand F, et al. Piloting an acceptable and feasible menstrual hygiene products disposal system in urban and rural schools in Bangladesh.BMC Public Health 2020;20(1):N.PAG-N.PAG. doi: 10.1186/s12889-020-09413-x . Language: English. Entry Date: In Process. Revision Date: 20210103. Publication Type: journal article. Journal Subset: Biomedical. 32894120 PMC7487504

[54] Kambala C, Chinangwa A, Chipeta E, Torondel B, Morse T. Acceptability of menstrual products interventions for menstrual hygiene management among women and girls in Malawi. Reprod Health 2020;17(1):N.PAG-N.PAG. Language: English. Entry Date: 20201127. Revision Date: 20201127. Publication Type: Article. doi: 10.1186/s12978-020-01045-z 33228723 PMC7686682

[55] Karibu K, Salami JC, Azeez M, Azeez A. Onset of Menarche and Adolescent Menstrual Hygiene Practices in Semi-Urban Ibadan Community, Nigeria.Onset of Menarche and Adolescent Menstrual Hygiene Practices in Semi-Urban Ibadan Community, Nigeria 2019;6(2):102–17. doi: 10.1080/23293691.2019.1601903

[56] Kemigisha E, Rai M, Mlahagwa W, Nyakato VN, Ivanova O. A qualitative study exploring menstruation experiences and practices among adolescent girls living in the nakivale refugee settlement, Uganda.Int J Environ Res Public Health 2020;17(18):1–11. doi: 10.3390/ijerph17186613 32932817 PMC7558145

[57] Kohler P, Renggli S, Lüthi C. WASH and gender in health care facilities: The uncharted territory.WASH and gender in health care facilities: The uncharted territory 2019;40(1):3–12. doi: 10.1080/07399332.2017.1395440 29116887

[58] Kumbeni MT, Otupiri E, Ziba FA. Menstrual hygiene among adolescent girls in junior high schools in rural northern Ghana.Pan Afr Med J 2020;37:190. doi: 10.11604/pamj.2020.37.190.19015 33447345 PMC7778209

[59] Lahme AM, Stern R, Cooper D. Factors impacting on menstrual hygiene and their implications for health promotion.Glob Health Promot 2018;25(1):54–62. doi: 10.1177/1757975916648301 27380769

[60] MacRae ER, Clasen T, Dasmohapatra M, Caruso BA. ’It’s like a burden on the head’: Redefining adequate menstrual hygiene management throughout women’s varied life stages in Odisha, India.PLoS One 2019;14(8):e0220114. doi: 10.1371/journal.pone.0220114 31369595 PMC6675075

[61] Mason L, Nyothach E, Alexander K, Odhiambo FO, Eleveld A, Vulule J, et al. ’We keep it secret so no one should know’—A qualitative study to explore young schoolgirls attitudes and experiences with menstruation in rural Western Kenya.PLoS One 2013;8(11). doi: 10.1371/journal.pone.0079132 24244435 PMC3828248

[62] Maulingin-Gumbaketi E, Larkins S, Gunnarsson R, Rembeck G, Whittaker M, Redman-MacLaren M. ’Making of a Strong Woman’: a constructivist grounded theory of the experiences of young women around menarche in Papua New Guinea. BMC Womens Health 2021;21(1):144. doi: 10.1186/s12905-021-01229-0 33832465 PMC8034129

[63] McHenga J, Phuma-Ngaiyaye E, Kasulo V. Do sanitation facilities in primary and secondary schools address Menstrual Hygiene needs? A study from Mzuzu City, Malawi.Phys Chem Earth 2020;115. doi: 10.1016/j.pce.2020.102842

[64] Miiro G, Rutakumwa R, Nakiyingi-Miiro J, Nakuya K, Musoke S, Namakula J, et al. Menstrual health and school absenteeism among adolescent girls in Uganda (MENISCUS): A feasibility study.BMC Womens Health 2018;18(1). doi: 10.1186/s12905-017-0502-z 29298699 PMC5753466

[65] Mohamed Y, Durrant K, Huggett C, Davis J, Macintyre A, Menu S, et al. A qualitative exploration of menstruation-related restrictive practices in Fiji, Solomon Islands and Papua New Guinea.PLoS One 2018;13(12). doi: 10.1371/journal.pone.0208224 30507969 PMC6277107

[66] Mohammed S, Emil Larsen-Reindorf R. Menstrual knowledge, sociocultural restrictions, and barriers to menstrual hygiene management in Ghana: Evidence from a multi-method survey among adolescent schoolgirls and schoolboys.PLoS One 2020;15(10 October). doi: 10.1371/journal.pone.0241106 33091080 PMC7580927

[67] Mohammed S, Larsen-Reindorf RE, Awal I. Menstrual Hygiene Management and School Absenteeism among Adolescents in Ghana: Results from a School-Based Cross-Sectional Study in a Rural Community.Int J Reprod Med 2020;2020:6872491. doi: 10.1155/2020/6872491 32411782 PMC7204135

[68] Mumtaz Z, Sivananthajothy P, Bhatti A, Sommer M. "How can we leave the traditions of our Baab Daada" socio-cultural structures and values driving menstrual hygiene management challenges in schools in Pakistan.J Adolesc 2019;76:152–61. doi: 10.1016/j.adolescence.2019.07.008 . 31487579

[69] Muralidharan A. Constrained Choices? Menstrual Health and Hygiene Needs Among Adolescents in Mumbai Slums.Indian J Gend Stud 2019;26(1–2):12–39. doi: 10.1177/0971521518808104

[70] Nalugya R, Tanton C, Hytti L, Kansiime C, Nakuya K, Namirembe P, et al. Assessing the effectiveness of a comprehensive menstrual health intervention program in Ugandan schools (MENISCUS): process evaluation of a pilot intervention study.Pilot Feasibility Stud 2020;6:51. doi: 10.1186/s40814-020-00585-2 32346485 PMC7181508

[71] Ndlovu E, Bhala E. Menstrual hygiene—A salient hazard in rural schools: A case of Masvingo district of Zimbabwe.Jamba 2016;8(2):1–8. doi: 10.4102/jamba.v8i2.204 29955312 PMC6014141

[72] Oche MO, Umar AS, Gana GJ, Ango JT. Menstrual health: The unmet needs of adolescent girls’ in Sokoto, Nigeria. Menstrual health: The unmet needs of adolescent girls’ in Sokoto, Nigeria 2012;7(3):410–8.

[73] Parker AH, Smith JA, Verdemato T, Cooke J, Webster J, Carter RC. Menstrual management: A neglected aspect of hygiene interventions.Disaster Prev Manag 2014;23(4):437–54. doi: 10.1108/DPM-04-2013-0070

[74] Rajagopal S, Mathur K. ‘Breaking the silence around menstruation’: experiences of adolescent girls in an urban setting in India.Gend Dev 2017;25(2):303–17. doi: 10.1080/13552074.2017.1335451

[75] Rajaraman D, Travasso SM, Heymann SJ. A qualitative study of access to sanitation amongst low-income working women in Bangalore, India.J Water Sanit Hyg Dev 2013;3(3):432–40. doi: 10.2166/washdev.2013.114

[76] Ramathuba DU. Menstrual knowledge and practices of female adolescents in Vhembe district, Limpopo Province, South Africa.Curationis 2015;38(1). doi: 10.4102/curationis.v38i1.1551 26841923 PMC6091664

[77] Rastogi S, Khanna A, Mathur P. Uncovering the challenges to menstrual health: Knowledge, attitudes and practices of adolescent girls in government schools of Delhi.Health Educ J 2019;78(7):839–50. doi: 10.1177/0017896919850209

[78] Rheinländer T, Gyapong M, Akpakli DE, Konradsen F. Secrets, shame and discipline: School girls’ experiences of sanitation and menstrual hygiene management in a peri-urban community in Ghana.Health Care Women Int 2019;40(1):13–32. doi: 10.1080/07399332.2018.1444041 29485336

[79] Rizvi N, Ali TS. Misconceptions and Mismanagement of Menstruation among Adolescents Girls who do not attend School in Pakistan.Journal of Asian Midwives 2016;3(1):46–62.

[80] Roxburgh H, Hampshire K, Kaliwo T, Tilley EA, Tilley EA, Oliver DM, et al. Power, danger, and secrecy-A socio-cultural examination of menstrual waste management in urban Malawi.PLoS One 2020;15(6 June). doi: 10.1371/journal.pone.0235339 32589649 PMC7319299

[81] Schmitt ML, Clatworthy D, Ratnayake R, Klaesener-Metzner N, Roesch E, Wheeler E, et al. Understanding the menstrual hygiene management challenges facing displaced girls and women: Findings from qualitative assessments in Myanmar and Lebanon.Confl Health 2017;11(1). doi: 10.1186/s13031-017-0121-1 29046714 PMC5641996

[82] Schmitt ML, Wood OR, Clatworthy D, Rashid SF, Sommer M. Innovative strategies for providing menstruation-supportive water, sanitation and hygiene (WASH) facilities: learning from refugee camps in Cox’s bazar, Bangladesh.Confl Health 2021;15(1):10. doi: 10.1186/s13031-021-00346-9 33637096 PMC7912835

[83] Scorgie F, Foster J, Stadler J, Phiri T, Hoppenjans L, Rees H, et al. “Bitten By Shyness”: Menstrual Hygiene Management, Sanitation, and the Quest for Privacy in South Africa.Med Anthropol 2016;35(2):161–76. doi: 10.1080/01459740.2015.1094067 26436841

[84] Shah V, Nabwera HM, Sosseh F, Jallow Y, Comma E, Keita O, et al. A rite of passage: A mixed methodology study about knowledge, perceptions and practices of menstrual hygiene management in rural Gambia.BMC Public Health 2019;19(1). doi: 10.1186/s12889-019-6599-2 30845945 PMC6407285

[85] Sheoran P, Kaur S, Lata H, Sarin J. A descriptive study of menstrual hygiene practices among women at the rural area of Haryana.Journal of Nursing & Midwifery Sciences 2020;7(4):269–73. doi: 10.4103/JNMS.JNMS_18_20 . Language: English. Entry Date: 20201103. Revision Date: 20201103. Publication Type: Article.

[86] Sivakami M, van Eijk AM, Thakur H, Kakade N, Patil C, Shinde S, et al. Effect of menstruation on girls and their schooling, and facilitators of menstrual hygiene management in schools: Surveys in government schools in three states in India, 2015.J Glob Health 2019;9(1). doi: 10.7189/jogh.09.010408 30546869 PMC6286883

[87] Sommer M, Ackatia-Armah N, Connolly S, Smiles D. A comparison of the menstruation and education experiences of girls in Tanzania, Ghana, Cambodia and Ethiopia. Compare 2015;45(4):589–609. doi: 10.1080/03057925.2013.871399

[88] Sommer M. Ideologies of sexuality, menstruation and risk: girls’ experiences of puberty and schooling in northern Tanzania.Cult Health Sex 2009;11(4):383–98. doi: 10.1080/13691050902722372 19326264

[89] Sommer M, Gruer C, Smith RC, Maroko A, Hopper K. Menstruation and homelessness: Challenges faced living in shelters and on the street in New York City.Health Place 2020;66. doi: 10.1016/j.healthplace.2020.102431 xsWOS:000594147200014. 32987242

[90] Tamiru S, Mamo K, Acidria P, Mushi R, Ali CS, Ndebele L. Towards a sustainable solution for school menstrual hygiene management: Cases of Ethiopia, Uganda, South-Sudan, Tanzania, and Zimbabwe.Waterlines 2015;34(1):92–102. doi: 10.3362/1756-3488.2015.009

[91] Tegegne TK, Sisay MM. Menstrual hygiene management and school absenteeism among female adolescent students in Northeast Ethiopia.BMC Public Health 2014;14(1). doi: 10.1186/1471-2458-14-1118 25355406 PMC4232635

[92] Trinies V, Caruso BA, Sogoré A, Toubkiss J, Freeman MC. Uncovering the challenges to menstrual hygiene management in schools in Mali. Waterlines 2015;34(1):31–40. doi: 10.3362/1756-3488.2015.004

[93] Umeora OU, Egwuatu VE. Menstruation in rural Igbo women of south east Nigeria: attitudes, beliefs and practices. Afr J Reprod Health 2008;12(1):109–15. doi: 10.2307/25470641 20695163

[94] Visaria L, Mishra RN. Health Training Programme for Adolescent Girls: Some Lessons from India’s NGO Initiative.J Health Manag 2017;19(1):97–108. doi: 10.1177/0972063416682586

[95] Wardell DW, Czerwinski B. A military challenge to managing feminine and personal hygiene.J Am Acad Nurse Pract 2001;13(4):187–93. doi: 10.1111/j.1745-7599.2001.tb00245.x 11930532

[96] Nepal WaterAid. Is Menstrual Hygiene and Management an Issue for Adolescent School Girls? A Comparative Study of Four Schools in Different Settings of Nepal. Kathmandu: WaterAid Nepal, 2009.

[97] Wilbur J, Kayastha S, Mahon T, Torondel B, Hameed S, Sigdel A, et al. Qualitative study exploring the barriers to menstrual hygiene management faced by adolescents and young people with a disability, and their carers in the Kavrepalanchok district, Nepal.BMC Public Health 2021;21(1):1–15. doi: 10.1186/s12889-020-10013-y . Language: English. Entry Date: In Process. Revision Date: 20210315. Publication Type: Article. Journal Subset: Biomedical. 33691653 PMC7944905

[98] Wilson E, Reeve J, Pitt A. Education. Period. Developing an acceptable and replicable menstrual hygiene intervention.Dev Pract 2014;24(1):63–80. doi: 10.1080/09614524.2014.867305

[99] Yeasmin F, Luby SP, Saxton RE, Nizame FA, Alam M, Dutta NC, et al. Piloting a low-cost hardware intervention to reduce improper disposal of solid waste in communal toilets in low-income settlements in Dhaka, Bangladesh. Piloting a low-cost hardware intervention to reduce improper disposal of solid waste in communal toilets in low-income settlements in Dhaka, Bangladesh 2017;17:1–11. doi: 10.1186/s12889-017-4693-x . Language: English. Entry Date: 20180723. Revision Date: 20180724. Publication Type: journal article. 28851334 PMC5576109

[100] Rees R, Oliver K, Woodman J, Thomas J. Children’s views about obesity, body size, shape and weight. University of London: Institute of Education; 2009. Available from: http://eppi.ioe.ac.uk/cms/Portals/0/Obesity%20Views%20Children%20R2009Rees.pdf?ver=2010-12-22-121209-040.

[101] NVivo qualitative data analysis software: QSR International Pty Ltd; Version 12, 2018.

[102] The World Bank. World Bank Country and Lending Groups. 2020 [18th November 2020]. Available from: https://datahelpdesk.worldbank.org/knowledgebase/articles/906519-world-bank-country-and-lending-groups.

[103] Bartram J, Charles K, Evans B, O’Hanlon L, Pedley S. Commentary on community-led total sanitation and human rights: should the right to community-wide health be won at the cost of individual rights? J Water Health 2012;10(4):499–503. doi: 10.2166/wh.2012.205 23165706

[104] Barrington DJ, Sridharan S, Shields KF, Saunders SG, Souter RT, Bartram J. Sanitation marketing: A systematic review and theoretical critique using the capability approach.Soc Sci Med 2017;194:128–34. doi: 10.1016/j.socscimed.2017.10.021 29100137

[105] Michael M. London’s fatbergs and affective infrastructuring.Soc Stud Sci 2020;50(3):377–97. doi: 10.1177/0306312720917754 32356482 PMC7411533

[106] ABC. Wet wipes, tampons, condoms targeted in new fatberg-fighting campaign. 2019. Available from: https://www.abc.net.au/radio/programs/pm/wet-wipes-tampons-condoms-targeted-fatberg-fighting-campaign/11339770.

