# Correction: Drivers of menstrual material disposal and washing practices: A systematic review

**DOI:** 10.1371/journal.pone.0316495

**Published:** 2024-12-20

**Authors:** Hannah Jayne Robinson, Dani Jennifer Barrington

In Table 3, there are errors in column “Code”. The “State of Available Facilities (40 *Studies*)” should have been “State of Available Facilities (57 *Studies*)”, the “Knowledge (11 *Studies*)” should be Knowledge (14 *Studies*) and the “Menstrual Taboos and Social Stigma (36 *studies*)” should be “Menstrual Taboos and Social Stigma (55 *studies*). Please see the correct Table 3 here.

**Table 3:** Codebook definitions and coding frequency.

	Code	Definition		Studies
**Practise** **(Deductive Codes)**	**Intentional Disposal **	**Menstruators chose to engage in a certain disposal technique (e.g. throwing into a latrine, field, jungle, canal, or bin; flushing down a toilet, burying them; wrapping them in newspaper, plastic, or paper; or leaving them on the toilet floor) **		56 (70%)
	**Washing, drying or reuse **	**Washing between uses to reuse materials; washing blood of materials for religious/cultural reasons before disposing; drying in the sun (on roofs/washing lines); or drying inside homes (open-air drying and hiding whilst drying) **		47 (59%)
**Reason / Behavioural Driver** **(Inductive Codes)**	**State of Available Facilities** *(57 Studies) *	Physical Infrastructure	Does the sanitation facility meet desired physical sanitation needs?	52 (65%)
		Social Perceptions	Does the sanitation facility meet desired social needs?	42 (53%)
	**Knowledge** *(14 Studies) *	Lack of knowledge	Menstruators have not been taught how to dispose / wash / dry materials	14 (18%)
	**Menstrual Taboos and Social Stigma** *(55 studies) *	Cultural Beliefs	General beliefs discouraging / encouraging certain methods of disposal	28 (35%)
		Embarrassment and Worry	Unpleasant emotions related to doing something considered by others to be wrong or shameful	35 (44%)
		Fear	Unpleasant emotion caused by the threat of danger, pain or other harmful consequences	13 (16%)

In the Reasoning behind behaviour subsection of the Results, references 19, 46 and 47 are omitted from the first sentence of the fourth paragraph. The correct sentence is: In 14 of the studies menstruators explicitly stated that they had been given no, or limited, advice regarding how to dispose of menstrual materials [19,21,24,28,30,37,45,46,47,64,74,78,81,83].

The references are:

19. Ahmmed F, Chowdhury MS, Helal SM. Sexual and reproductive health experiences of adolescent girls and women in marginalised communities in Bangladesh.Cult Health Sex 2021:1–16. pmid:33941046

46. Habtegiorgis Y, Sisay T, Kloos H, Malede A, Yalew M, Arefaynie M, et al. Menstrual hygiene practices among high school girls in urban areas in Northeastern Ethiopia: A neglected issue in water, sanitation, and hygiene research.PLoS One 2021;16(6):e0248825. pmid:34106948

47. Hawkins A, Sharpe R, Spence K, Holmes N. Inappropriate flushing of menstrual sanitary products.Proceedings of the Institution of Civil Engineers-Water Management 2019;172(4):163–9. WOS:000475712900001.
