# Supplementary material for: Drivers of menstrual material disposal and washing practices: A systematic review
Source: PLoS One. 2021 Dec 3;16(12):e0260472. doi: 10.1371/journal.pone.0260472 (PMC8641861; doi:10.1371/journal.pone.0260472)
Supplement: S1 Table — (DOCX) [file pone.0260472.s002.docx]

| Citation | Country | Economic status | Population | Sample Size | Data Collection Method | Qualitative, Quantitative, or mixed-methods | Disposal/ Washing Behaviour | Reason for behaviour | Reason provided by | Reliability | Usefulness |
| --- | --- | --- | --- | --- | --- | --- | --- | --- | --- | --- | --- |
| Abera, 2004 (1) | Ethiopia | Low | Girls in school, school Staff | Girls in School (*grades 9-10 (≈ aged 14-16), across 8 schools)*, and School Staff  Breakdown of school students not specified. | 863 Close-end structured, self-administered questionnaires, 4 focus group discussions *(8 students in each)*, and key informant interviews with school authorities *(number unspecified),* and checklist for observation of facilities available | Qualitative | Predominantly disposed into latrines (75%). Also noted throwing into bins and open fields. | Absence of bins, absent or broken incinerators | Author inferred | Medium | Medium |
| Ahmmed et al, 2021 (2) | Bangladesh | Lower-middle | Adolescent girls, Women, Birth Attendants and Medicine Vendors | 89 married women *(reproductive age),* 42 adolescent girls *(aged 14-18)*, 18 elderly women, 3 traditional birth attendants, 3 medicine vendors | 131 in-depth interviews *(with married women and adolescent girls),* 3 focus groups *(with elderly women),* 6 key informant *interviews (with birth attendants and medicine vendors)* | Qualitative | Choosing not to dry cloths openly | Worries that men would see, and concerns of attacks from evil spirits | Author inferred |  |  |
|  |  |  |  |  |  |  | *(no specific behaviour given)* | Unawareness amongst menstruators of options available to manage | Author inferred |  |  |
| Alda-vidal and Browne, 2021 (3) | Malawi | Low | Women | 40 Women, 13 sanitation workers, 15 external MHM actors | 47 semi-structured interviews (19 women, 13 sanitation workers, 15 external MHM actors), and 5 focus groups *(4-8 women, 21 total participants)* | Qualitative | Keeping all materials hidden from view | Worries that if women are ‘careless’ they may have ‘complications or die’ due to association of menstrual blood with magic and witchcraft practice | Participants |  |  |
|  |  |  |  |  |  |  | Washing reusable materials immediately after changing | Beliefs that it would reduce risks associated with witchcraft | Participants |  |  |
|  |  |  |  |  |  |  | Burning or throwing reusable items in pit latrines | Lack of water and soap to allow for hygienic practices | Participants |  |  |
|  |  |  |  |  |  |  | Burning used menstrual materials | As to not fil up pit latrines too quickly, or due to inability to flush down toilets | Participants |  |  |
| Alexander et al, 2014 (4) | Kenya | Lower-middle | School staff | 62 Headteachers | 62 Structured interviews and observation of facilities | Qualitative | Disposal into latrines, carried home from school, burned in rubbish pit | Appropriate disposal was “inconsistent, unknown and unsustainable” across study schools (predominantly poor condition of WASH facilities) | Author inferred (*and described by head teachers)* | Medium | Low |
| Asimah et al, 2017 (5) | Ghana | Lower-middle | Girls in school, guardians | 319 pupils *(aged 10-19, with 229 females, 90 males across 15 schools)*, and 333 household heads *(241 males, 92 females)* | 319 ‘Knowledge, attitude, practise, belief’ (KAPB) surveys, focus group discussions with pupils, and 333 in-depth interviews with household heads | Qualitative | Predominantly disposed in the toilet or bin (61%), but also noted burying, throwing into bushes, refuse dump, or in the incinerator. | To *allow* menstrual blood to be used for rituals (uncommon)  Due to lack of access to facilities | Author inferred | Medium | Medium |
| Averbach, et al, 2009 (6) | Zimbabwe | Lower-middle | Women | 43 women *(aged 18-45)* | 43 Self-administered surveys and focus group discussions | Mixed-methods | Washing without soap | Unavailability/ expensive cost of soap | Participants | High | Medium |
|  |  |  |  |  |  |  | Drying materials inside the home | Secrecy and shame preventing open air outdoor drying | Participants and inferred by Author |  |  |
| Behera et al, 2015 (7) | India | Lower-middle | Adolescent girls | 32 adolescent girls *(female, aged 14-15)* | 5 focus group discussions (guidelines and unstructured questions used) | Qualitative | Predominantly disposed of in bins after wrapping in paper, few threw into open space behind school. | Lack of awareness about proper disposal methods (re: throwing into open spaces) | Author inferred | High | Medium |
|  |  |  |  |  |  |  | Those reusing materials washed with plain soap and water (few used antiseptics) | Lack of access to antiseptic soap/liquid | Participants |  |  |
|  |  |  |  |  |  |  | Disposing of used menstrual materials into bins | Avoiding disposing in the toilet as it creates issues for sewer companies | Participant *(male)* |  |  |
|  |  |  |  |  |  |  | *(no specific behaviour given)* | 30% not aware of environmental concerns associated with disposable products | Participants |  |  |
| Bhattacharjee, 2019 (8) | India | Lower-middle | Women and Adolescent Girls | 84 Women and adolescent girls *(aged 15-50, across 3 villages)* | Semi-structured interviews and focus group discussions | Qualitative | Only partially drying reusable materials before storing | Preference to not dry in the open where people could see the materials | Author Inferred |  |  |
|  |  |  |  |  |  |  | Disposing of used pads in bags filled with pebbles and throwing into the river | Pebbles were used as to weigh down the bags so they did not float, lessening chances of it being seen | Author Inferred |  |  |
|  |  |  |  |  |  |  | Being unable to change materials | Lack of time given to menstruators | Participants |  |  |
| Caruso et al, 2017 (9) | India | Lower-middle | Women | 115 women *(aged 18-75)* | 69 Free-list interviews and 8 focus group discussions *(totalling 46 participants)* | Qualitative | Disposing of materials by throwing into the jungle, rivers, ponds, and forested areas | Lack of toilet to throw in, and worries around throwing outside for fear of being seen | Participants | High | High |
|  |  |  |  |  |  |  | Washing at night or out of view of others | Lack of safe convenient place to wash, dry and dispose of menstrual materials out of sight of others | Participants |  |  |
| Caruso et al, 2014 (10) | Kenya | Lower-middle | Girls in school, school staff | 36 students *(female, aged 11-17, across 3 primary schools for focus groups)*, 6 students *(selected from the focus group discussions, for in-depth interviews)*, 2 teachers *(for in-depth interviews)* | Two free-listing focus group *discussions (one with girls, one with boys in each school)*, in depth interviews *(6 pupils, 2 staff members)*, latrine walks, | Qualitative | Disposing of materials by throwing in latrine | To maintain privacy and to avoid carrying soiled materials elsewhere | Participants | High | Medium |
| Chakravarthy et al, 2019 (11)  *(Paper uses 3 studies – 1 available report and 2 unpublished documents)* | India | Lower-middle | Women & girls, Government officials | Unspecified number of adolescent girls *(aged 10-19)* women *(aged 20-49)* and 20 government officials.  Breakdown of participants not specified. | 45 focus group discussions *(35 with adolescent girls, 10 with women),* 20 in-depth interviews | Qualitative | Throwing pads in/around the toilet *(pre-intervention)* | Lack of bins/disposal mechanisms | Participants | Low | Medium |
|  |  |  |  |  |  |  | Placing old cardboard boxes in the toilets lined with newspaper for temporary bins *(after intervention)* | Participants realised they had a role to keep sanitation facilities clean/ hygienic | Participants |  |  |
|  |  |  |  |  |  |  | Wrapping pads before throwing them away | To conceal their blood | Author Inferred |  |  |
|  |  |  |  |  |  |  | *(no specific behaviour given)* | Apprehension to use facilities where blood could be seen in drains when changing | Author Inferred |  |  |
|  |  |  |  |  |  |  | *(no specific behaviour given)* | Agreement that dumping pads was bad for sanitation, but there was not an alternative | Author Inferred |  |  |
| Chinyama et al, 2019 (12) | Zambia | Lower-middle | Girls in school,  school staff, guardians, | 64 students *(aged 14-18, 48 female, 16 male, for 8 focus group discussions)*, 12 students *(aged 14-18, female, for in-depth interviews)*, 7 teachers *(for key informant interviews)*, (all across 6 schools), 7 guardians *(for key informant interviews)*, and 11 leaders *(both male and female) (for key informant interviews)* | 12 in-depth interviews, 8 focus groups *(6 female groups, 2 male groups),* 25 key informant interviews | Qualitative | Disposing of materials in latrines (as opposed to available bins) | For fear of witches and Satanists getting hold of waste “to rob them of their fertility” | Participants | High | Medium |
|  |  |  |  |  |  |  | *(no specific behaviour given)* | Lack of provision of adequate disposal systems | Author inferred |  |  |
| Chothe et al, 2014 (13) | India | Lower-middle | Girls in school | 381 students *(female, aged 9-13)* | Qualitative ethnographic research – students posed open-ended questions about MHH to paediatricians | Qualitative | *(no specific behaviour given)* | Fear of becoming infertile if animals see/smell | Participants | High | Low |
|  |  |  |  |  |  |  | *(no specific behaviour given)* | Lack of knowledge of how to dispose of menstrual materials | Participants and author inferred |  |  |
| Connolly and Sommer, 2013 (14) | Cambodia | Lower-middle | Adolescent girls, school staff, guardians | 146 adolescent girls *(female, aged 16-19, mix of in and out of school)*, and 15 parents/ teachers | Ethnographic observation, 15 key informant interviews, participatory activities with 9 groups *(146 girls across 9 groups)* | Qualitative | *(no specific behaviour given)* | Lack of bins inside toilet stalls. Students reported avoiding using externa bins as they’d be seen by others | Author inferred | Medium | Medium |
| Coswosk et al, 2019 (15) | Brazil | Upper-middle | Girls in school, school staff | School principal and vice-principal, 39 students *(female and male, aged 13-17)* | Participant observation, structured focus groups *(4 groups, 29 girls and 10 boys),* individual semi-structured interviews | Qualitative | Leaving used menstrual materials wrapped in paper on the toilet floor, or throwing into the latrine | Wrapping to hide the contents, and throwing into the latrine due to lack of bin | Participants | Medium | Medium |
| Crankshaw et al, 2020 (16) | South Africa | Upper-middle | Girls in school, Boys in School, School staff, Mothers of Girls in School | 505+ students *(across 10 schools)*, 8 teachers, 9 mothers of students,  Breakdown of school students not specified. | 505 quantitative surveys *(with girls in school),* 47 semi-structured interviews *(30 girls, 8 school staff, 9 mothers)*, | Mixed-methods | Wrapping used menstrual materials and taking them home from school to dispose, or throwing used items in the toilet, burning or burying them *(less common)* | No/few bins in school facilities *(or dislike of using the bins therefore no disposal option)* | Participants |  |  |
|  |  |  |  |  |  |  | Changing only at home | Unusable WASH facilities without water and soap, and some school staff refusing to let students use the facilities during lessons | Participants |  |  |
| Crichton et al, 2013 (17) | Kenya | Lower-middle | Adolescent girls | 87 students *(aged 12-17)*, 69 women, 5 teachers, 1 nurse | Open-ended in-depth interviews *(15 with girls and 14 with women),*  focus groups discussions *(10 with girls and 8 with women),* key informant interviews *(5 teachers and 1 community nurse)* | Qualitative | Used menstrual materials discarded in streets | Lack of refuse collection facility | Author Inferred | High | Medium |
|  |  |  |  |  |  |  | *(no specific behaviour given)* | Fear of next user seeing used menstrual materials if discarded in latrine | Participants |  |  |
| Crofts and Fisher, 2011(18)  And  Crofts and Fisher, 2012 (19) | Uganda | Low | Girls in school school staff, business leaders | 134 students *(female, aged 13-20, for participatory activities and FDGs)*, 9 business leaders, 12 school staff | Participatory activities *(undertaken by 134 girls)*, 40 focus groups *(across 18 schools)*, 21 key informant interviews, and observations of facilities *(across 18 schools)* | Qualitative | Mainly washing menstrual materials in bathing shelters, some washed in the dormitories | Deemed culturally inappropriate to wash in public spaces | Participants | Medium | High |
|  |  |  |  |  |  |  | Only 3% dried in sunlight, 1/7 noted chafing and infection from damp materials | Avoiding hanging pads in public display | Participants |  |  |
|  |  |  |  |  |  |  | Throwing used menstrual materials in pit latrines | Lack of bins inside toilets | Participants |  |  |
|  |  |  |  |  |  |  | *(no specific behaviour given)* | Separated incinerators and latrines, causing worry if girls seen moving between them | Author inferred |  |  |
|  |  |  |  |  |  |  | *(no specific behaviour given)* | Fears of becoming infertile if left around for animals to find | Author inferred |  |  |
| Daniels, 2016 (20) | Cambodia | Lower-middle | Adolescent girls,  adolescent boys, women, men, school staff | 165 participants *(for interviews)*, 181 participants *(for focus group discussions)*, including girls, boys, mothers, fathers, and teachers. Breakdown of participants not specified. | 165 Structured interviews and 24 structured focus group discussions (with a total of 181 participants) | Qualitative | Placing used menstrual materials in plastic bags and then burning/ throwing away/ burying | Lack of a private place to dispose of materials | Author Inferred | High | Medium |
|  |  |  |  |  |  |  |  |  |  |  |  |
| Dhingra et al, 2009 (21) | India | Lower-middle | Adolescent girls | 200 girls *(aged 13-15)* | In-depth interviews and focus group discussions *(groups of 2-3),* and clinical observations *(1 group a week, 20 girls)* | Qualitative | 88.5% of the adolescent girls washed their cloth (then dried on a corner), then reused or buried it | Cloth was washed before burying for religious practises | Participants | Medium | Low |
|  |  |  |  |  |  |  | Cloth dried in ‘secret’ | To hide the material from others | Author inferred |  |  |
| Dolan et al, 2014 (22) | Ghana | Lower-middle | Girls in school, parents,  school staff | 99 girls *(age unspecified, for interviews)*, 136 girls *(age unspecified, including dropouts, for focus group discussions)*, 246 parents, 12 school staff *(for key informant interviews)*, 156 school staff *(for focus group discussions)* | 111 Semi structured interviews *(99 girls, 12 school staff),* focus group discussions *(136 girls, 246 parents, 156 school staff)* | Qualitative | Burying used pads in the forest | Feared pads would be retrieved by others or seen and used for witchcraft | Author inferred | Medium | Low |
|  |  |  |  |  |  |  | Hiding cloth under beds to dry | *(no specific reasoning given)* |  |  |  |
| Ellis et al, 2016 (23) | Philippines | Lower-middle | Girls in school | 79 students (female, aged 11-18, across 3 schools in urban Manilla, and 10 rural schools) | 13 Focus group discussions *(typically 6-8 girls each)* | Qualitative | Avoided using water – *problematic specifically for Muslim girls wanting to wash blood off cloths before disposing* | Large water drums too tall for students to access, students feared falling in, and water was ‘dirty. Also, cultural beliefs dictating washing before disposal | Author inferred | High | High |
|  |  |  |  |  |  |  | Bins were not used in ‘non- sex-segregated toilets | Girls were afraid of boys seeing/smelling used menstrual materials so avoided disposing of used menstrual materials into provided bins | Author inferred |  |  |
|  |  |  |  |  |  |  | Used menstrual materials were wrapped before being disposed of | Girls wrapped materials to prevent a smell | Participants |  |  |
|  |  |  |  |  |  |  | Throwing used menstrual materials behind the school or into the canal | *(no specific reasoning given)* |  |  |  |
| Enoch et al, 2020 (24) | Ghana | Lower-middle | Adolescent girls | 18 adolescent girls *(aged 12-19, with visual, hearing or physical disabilities (6 girls for each disability))* | 18 In-depth interviews and 3 focus group discussions *(6 members each)* | Qualitative | Burning used menstrual materials | To ensure materials are not stolen by dogs and left at shrines, as worries of infertility charms | Participants |  |  |
|  |  |  |  |  |  |  | Wrapping in toilet roll before disposing menstrual materials | Worries that is others see their blood, their periods will stop and they will become sick | Participants |  |  |
| Garikipati and Boudot, 2017 (25) | India | Lower-middle | Adolescent girls and women | 150 women and adolescent girls *(aged 15-49, from 3 slum locations)* | 150 semi structured interviews (questionnaires with categorical and open-ended answers), and observation of local shops (that sold menstrual materials) | Mixed-methods | 29% of participants reported not drying used cloth | Disposal practises predominantly tied into beliefs about blood being used for witchcraft (causing infertility and blindness) | Participants | Medium | High |
|  |  |  |  |  |  |  | Burying or burning used menstrual materials | 13% of the sample stated wanting to change products, but being unable due to restricted disposal | Participants |  |  |
| George and Leena, 2020 (26) | India | Lower-middle | Women | 22 women *(aged 25-49)* | 22 semi-structured interviews | Qualitative | General difficultly in disposing *(no specific behaviour given)* | Homes are too close to burn used menstrual materials | Participants |  |  |
|  |  |  |  |  |  |  | Only changing/ washing menstrual cup at home | Worries that public toilets are not clean enough to safely clean the material | Participants |  |  |
| Girod et al, 2017 (27) | Kenya | Lower-middle | Girls in school, school staff | 51 students *(approximately – number of students not explicitly stated, female, grades 6-8 (≈ aged 11-14), across 6 different primary schools) and* 6 Headteachers | 6 focus group discussions *(6-11 participants each, 1 discussion at each school),* 6 key informant interviews *(with head teacher at each school),* observation of school facilities, and an anonymous question session | Qualitative | Girls kept used menstrual materials in pockets as opposed to available bins | Girls did now want other students to see they were menstruating | Participants | High | High |
|  |  |  |  |  |  |  | Throwing used menstrual materials on the floor | Could not wait for a stall with a bin to become available | Participants |  |  |
| Gultie et al, 2014 (28) | Ethiopia | Low | Girls in school | 492 students *(female, grades 9-12, aged 13-21+)* | 492 Structured self-administered questionnaire | Quantitative | 69.3% disposed used menstrual materials in school latrine, 14.4% threw into a bin, 9.3% took materials home, 6.3% threw into open fields, 0.4% buried them | 39.2% had no private space | Participants | High | Medium |
|  |  |  |  |  |  |  |  | 10.0% had no disposal option |  |  |  |
| Habtegiorgis et al, 2021 (29) | Ethiopia | Low | Girls in School | 536 students *(female, aged 13-19, across 5 schools (3 public, 2 private, 457:79)* | 536 structured pre-tested questionnaires and observational checklist for WASH facilities | Mixed-methods | 41.9% dried reusable materials without sunlight | Students did not want to be seen handling absorbents outside | Author inferred |  |  |
|  |  |  |  |  |  |  | Avoided going to school whilst menstruating | No available place to wash, or dispose of used materials | Participants |  |  |
|  |  |  |  |  |  |  | *(no specific behaviour given)* | Lack of knowledge of how to manage menstruation | Participants |  |  |
| Hawkins et al, 2019 (30) | UK | High | Women | 10 women *(female, aged 18-30)* | in-depth semi-structured interviews | Qualitative | Not flushing menstrual pads down toilets | Both environmental concerns and fears of blocking or breaking the toilet | Participants |  |  |
|  |  |  |  |  |  |  | Flushing tampons down toilets | Beliefs that these materials were ‘flushable’, or due to worries of being embarrassed for used products to be seen in the bin | Participants |  |  |
|  |  |  |  |  |  |  | Wrapping up used products and taking them home to dispose of | Limits the probability other women in toilets would see used products if disposal is not within an individual cubicle | Participants |  |  |
|  |  |  |  |  |  |  | Avoidance of using reusable products in public | ‘Incompatibility’ of reusable products when using shared handwashing basins | Participants |  |  |
|  |  |  |  |  |  |  | *(no specific behaviour given)* | 10.0% had no disposal option |  |  |  |
| Hennegan et al, 2020a (31) | Uganda | Low | Women | 35 Women *(female, aged 18-35)* | 35 semi-structured  in- depth interviews | Qualitative | Drying materials inside the house | Menstrual materials seen as ‘too private’ for people to see | Participants |  |  |
|  |  |  |  |  |  |  | Forced to throw used menstrual materials in the latrine | Infrequent cleaning of bins left them overflowing and unusable | Participants |  |  |
|  |  |  |  |  |  |  | Avoidance of using work facilities to change, so using nearby businesses or travelling home | Menstruators described work facilities were too dirty, costly or insufficiently private for changing | Participants |  |  |
| Hennegan and Sol, 2020 (32) | Bangladesh | Lower-middle | Girls in school | 1359 students *(female, aged 10-16, across 149 schools, (approximately 9 students per school))* | School intervention *(345 students),* Community intervention and school intervention *(373),* and a control group *(641).* | Mixed-methods | Burying used materials or hiding them outdoors | Methods favour discretion | Author inferred |  |  |
| Hennegan et al, 2017 (33) | Uganda | Low | Girls in school | 27 students *(female, aged 12-17, across 8 schools)* | 27 In-depth semi-structured interviews | Qualitative | Disposable pads predominantly disposed in pit latrines | *(no specific reasoning given)* |  | High | High |
|  |  |  |  |  |  |  | Instances of washing and drying reusable pads in the evening and drying inside | Hiding materials from others | Author inferred |  |  |
| Hennegan et al, 2016 (34) | Uganda | Low | Girls in school | 205 students *(female, aged 10-19, across 8 schools)* | 205 surveys *(following up a control trial on MHM)* | Quantitative | 58% of girls dried materials inside, 47% of these were ‘hidden inside’ | Worries material may be seen | Author inferred | High | Medium |
|  |  |  |  |  |  |  | *(no specific behaviour given)* | 76.7-91.5% of girls were ‘worried about being observed washing *[their materials]*’ | Participants |  |  |
|  |  |  |  |  |  |  | Carrying used menstrual materials home to clean/dispose from school | To wash at home, possible causing increased anxiety and stigma | Author inferred |  |  |
| Htun et al, 2021 (35) | Myanmar | Lower-middle | Adolescent girls | 410 adolescent girls *(aged 9-15, across 38 villages)* | 410 structured interviews | Quantitative | Menstruators did not dry used materials in direct sunlight | Feelings of shame and limited space | Author inferred |  |  |
| Jahan et al, 2020 (36) | Bangladesh | Lower-middle | Girls in school | Pre-intervention Period (PrIP):168 students and 17 school staff  Intervention Design Period (IDP): 139 students and 12 school staff  Post-intervention Period (PIP): 100 students and 20 school staff  468 individuals, including students (419), teachers (21), and janitors (28)  *All students aged 12-16* | In-depth-interviews with girls *(48 PrIP, 139 IDP, 100 PIP),* In-depth-interviews with janitors *(4 PrIP, 20 IDP),* 4 participatory ranking exercises *(PrIP),* 4 drawing exercise *(PrIP),* 4 vignette exercise *(PrIP),* 4 focus group discussions *(IDP - with school staff and janitors),* and spot checks of facilities *(12 IDP, 20 PIP)* | Mixed-methods | Avoiding changing materials when at school | No disposal facility on school premises, and WASH facilities had a general low level of cleanliness and bad odours | Participants |  |  |
|  |  |  |  |  |  |  | Throwing used materials into the latrine | No disposal facility on school premises | Participants |  |  |
|  |  |  |  |  |  |  | Burying or disposing of used  cloths and pads | Beliefs that heavy bleeding or abdominal pain may occur if they wash and dry the cloths in the open. | Participants |  |  |
| Kambala et al, 2020 (37) | Malawi | Low | Women, girls in school, school staff, community leaders, community health workers, and MHM service providers | 80 students *(female, aged 10-18)*, 61 women, 12 school staff, 6 community leaders, 8 community health workers, and 9 MHM service providers | 46 in-depth interviews *(20 girls, 26 women),* 35 key informant interviews *(12 teachers, 6 community leaders, 8 community health workers, and 9 MHM service providers)),* and 11 focus group discussions *(35 women (over 4 groups), 60 girls (over 7 groups))* both across 8 districts. | Qualitative | Difficult to always ensure reusable materials were washed with soap | Lack of availability of soap | Participants |  |  |
|  |  |  |  |  |  |  | Avoidance of burying disposable pads | Due to the perceived long time to decompose due to plastic composition | Participants |  |  |
|  |  |  |  |  |  |  | Ensuring that materials aren’t disposed of in pit latrines/toilets | Increased chance of blockages/filling up latrines too quickly | Participants |  |  |
|  |  |  |  |  |  |  | Drying pads in light spaces | Limit chances for flies to breed and decrease risk of infections | Participants |  |  |
| Karibu et al, 2019 (38) | Nigeria | Lower-middle | Adolescent girls | 492 adolescent girls *(aged 10-19, covering both those in and out of school)* | 492 structured interviews with a 69-item questionnaire | Qualitative | 53.1% of girls disposed of used products in bins, and 14.0% stated into latrines | 38.0% chose their method to ‘ensure protection from metaphysical forces’, 8.9% said it would reduce environmental contamination, 2.4% said they chose a method to avoid ‘evil people’ | Participants | High | Medium |
|  |  |  |  |  |  |  | Girls identified choosing ‘poor hygiene’ practises | 37% attributed this to lack of finance, 14.2% attributed to cultural taboos, 14% attributed to laziness | Participants |  |  |
|  |  |  |  |  |  |  | 53.9% of girls dried materials inside after washing, 30.0% dried outside | Those that dried outside did so to reduce infection | Author inferred |  |  |
| Kemigisha et al, 2020 (39) | Uganda | Low *(refugee settlement)* | Adolescent girls | 28 adolescent girls *(aged 13-19)* | 28 semi-structured interviews and 2 focus groups | Qualitative | Washing reusable materials with soap and drying outside | For good hygiene and to decrease risks of infection | Author inferred |  |  |
| Kohler et al, 2019 (40) | India, Uganda | Lower-middle and low | Women and men *(inpatients and healthcare staff)* | 50 Indian participants and 40 Ugandan participants *(across 4 hospitals, for workshops, interviewees selected from this sample)*.  Both samples included in-patients and staff. | 6 GALS Workshops *(2 in India – 50 participants (28 female and 22 male), 4 in Uganda – 40 participants (20 female and 20 male)),* 72 semi-structured interviews *(36 in each country),* and observation of facilities | Qualitative | Throwing used menstrual materials into toilets or out of windows | Lack of disposal option, and no place to change/ wash/ dry materials | Participants | High | Medium |
|  |  |  |  |  |  |  | Choosing not to use bins provided | Cultural beliefs around witchcraft *(Uganda)* and impurity *(India)* | Author inferred |  |  |
| Kumbeni et al, 2020 (41) | Ghana | Lower-middle | Girls in school | 730 students *(female, aged 10-19, across 15 schools)* | Cross-sectional study with 730 structured questionnaires and a WASH facility checklist | Mixed-methods | 68.9% washed and dried their reusable materials inside rooms | Worried they will get teased if their materials are seen by others | Author inferred |  |  |
| Lahme et al, 2018 (42) | Zambia | Lower-middle | Girls in school | 51 students *(female, aged 13-20, across 3 schools)* | 6 focus group discussions *(2 in each school, each with 8-10 students)* | Qualitative | Wrapping used menstrual materials in newspaper and hiding in school bags | Lack of disposal options at school | Participants | Medium | Medium |
| MacRae et al, 2019 (43) | India | Lower-middle | Women | 114 Women *(across 12 communities – 39 unmarried women, 12 recently married women, 38 married women, 25 older women)* | 68 Free-list interviews *(across 8 communities -*  *16 UMW, 12 RMW, 22 MW, 18 OW)* and 8 focus groups *(5-7 participants each, across 4 communities -*  *23 UMW, 16 MW, 7 OW)* | Qualitative | Hiding used materials if no formal disposal location was available | To obscure the view of used materials | Author inferred |  |  |
|  |  |  |  |  |  |  | Disposal of used materials into ponds, rivers, jungles, or burying in the ground | Lack of formal disposal system | Participants |  |  |
|  |  |  |  |  |  |  | When washing reusable materials, menstruators poured water on the ground around the tube wells | In order to conceal signs of blood from others | Participants |  |  |
|  |  |  |  |  |  |  | Hiding used materials in dirty places to wash at a later time | Waiting until there was privacy to wash materials, and not wanting materials inside the home | Participants |  |  |
|  |  |  |  |  |  |  | Drying materials on roofs, in bushes, in cow sheds, in dark places inside homes, and underneath other clothing *(but acknowledged the potential dangers of these practises in terms of infection risk)* | Menstruators felt ashamed if other people saw the materials whilst drying | Participants |  |  |
|  |  |  |  |  |  |  | Avoidance of disposal methods that would allow animals to come into contact with materials | Worries of increased bleeding, infertility | Participants |  |  |
| Mason et al, 2013 (44) | Kenya | Lower-middle | Girls in school | 120 students *(female, aged 14-16, cross 6 schools)* | 11 focus group discussions *(each with 7-13 participants, set across the 6 schools)* | Qualitative | *(no specific behaviour given)* | Fear of having menstrual materials/ blood being seen and consequentially seen as an ‘adult’ | Participants | High | Medium |
|  |  |  |  |  |  |  | Washing materials without soap | Lack of availability | Participants |  |  |
|  |  |  |  |  |  |  | Dried reusables at night or hidden under clothes | Prevent others seeing their materials | Participants |  |  |
|  |  |  |  |  |  |  | Avoiding disposal in latrines or burning them | Girls stated wanting to dispose of them ‘some place no one can see’ | Participants |  |  |
| Maulingin-Gumbaketi et al, 2020 (45) | Papua New Guinea | Low-middle | Women | 98 women *(aged 13-45+, across 4 provinces)* | 6 semi-structured interviews and 10 focus groups | Qualitative | Putting soiled items in the general rubbish bin | Unavailability of disposal facilities | Participants |  |  |
|  |  |  |  |  |  |  | Burning used materials at night | Lack of disposal facilities, and decreased chances of others seeing | Participants |  |  |
| McHenga et al, 2020 (46) | Malawi | Low | Girls in school and school staff | 228 students *(female, aged 11-22)*, 22 school staff *(Head Teachers and Senior female teachers)* | 228 interviews *(131 primary and 97 secondary students)*, 13 Focus Group Discussions *(with students),* 22 key informant interviews *(with Head Teachers and Senior female teachers)* and observations of WASH facilities | Qualitative | Not changing materials at school | Minimal privacy due to lack of doors and locks on latrines | Participants |  |  |
|  |  |  |  |  |  |  | Going home to change materials | Lack of disposal options for used materials | Participants |  |  |
| Miiro et al, 2018 (47) | Uganda | Low | Girls in school,  boys in school, school staff,  Municipality officials,  parents | 562 students *(352 female and 210 male, aged 13-18, across 4 schools)*, 11 teachers, 2 municipality officials *(Ministry of Education and the Ministry of Health),* 10 parents | 16 in-depth interviews (with 4 girls from each school), 8 Group Interviews *(each with 8-12 girls)*, 14 key-informant interviews, 562 questionnaire participants, WASH facility observation, written diary entries for 10 girls. | Mixed-methods | *(no specific behaviour given)* | Need for private disposal option for used menstrual materials and private space where male students cannot see into (48% of pit latrines without locks) | Author inferred | High | Medium |
|  |  |  |  |  |  |  | Not being able to access clean water to clean materials/ themselves | Due to taps being locked, and water and soap not being provided inside toilets |  |  |  |
| Mohamed et al, 2018 (48) | Fiji, Papua New Guinea, Solomon Islands | Upper-middle, lower-middle | Women & girls, men, school staff, community members (*including vendors, employers, health workers, community leaders and vulnerable women)* | 54 girls in school *(aged 13-26),* 43 gdolescent girls *(aged 13-29),* 118 women *(aged 19-61),* 51 men *(aged 23-70),* 8 school staff, and 34 community members | 31 focus group discussions,  8 in-depth interviews and 34 key informant interviews *(across 3 countries)* | Qualitative | Washing pads prior to disposal/ burning | Thoughts of menstrual blood bringing ‘bad luck’/ black magic | Participants | High | High |
|  |  |  |  |  |  |  | Drying reusable materials under larger items, or drying inside the house | To hide materials from sight | Author inferred |  |  |
| Mohammed et al, 2020a (49)  And  Mohammed et al, 2020b (50) | Ghana | Lower-middle | Girls in school, boys in school and 5 school staff | 280 Students *(250 female, aged 10-19, across 5 schools; 30 male, across 3 schools)* and 5 head teachers | 250 quantitative questionnaires, focus group discussions *(with 30 boys)*, and 5 key informant interviews *(with head teachers)* | Mixed-methods | Disposing of used materials into pit latrines | Lack of disposal facilities at school, and to decrease the chance of someone seeing their used materials | Author inferred |  |  |
| Mumtaz et al, 2019 (51) | Pakistan | Lower-middle | Girls in school, women, school staff, care providers, local religious leaders and a scholar | 312 students *(female, aged 16–19 years)*, 15 mothers, 11 female school teachers, 9 health care providers, 5 local religious leaders and 1 scholar | Participatory activities and informal discussions with 312 students, observations of 7 School WASH facilities, 42 key informant interviews | Mixed-methods | Reusable materials were dried in dark, often dirty corners inside homes, with teachers stating to never dry materials ‘under an open sky’ | To ensure men would not see the bloodstained materials and to hide menstrual blood from evil spirits *(Jinns)* | Author inferred |  |  |
|  |  |  |  |  |  |  | All materials (reusable and disposable) were first washed and then wrapped in a plastic bag before disposal | To ensure no man would see any menstrual blood *(which was identified as a sin)* | Author inferred |  |  |
|  |  |  |  |  |  |  | Hiding all materials and underlying secrecy with all management practises | Possibility of early marriage if fathers became aware of their daughters apparent ‘sexual maturity’ and fears that menstrual blood could be used for black magic with evil spirits | Participants |  |  |
|  |  |  |  |  |  |  | Staying at home for the first days of menstruation each month to manage at home | Inability to manage menstruation at school due to ‘dirty, gender unfriendly WASH facilities’ | Author inferred |  |  |
| Muralidharan, 2019 (52) | India | Lower-middle | Women & girls | Up to 72 Adolescent girls *(aged 15-24)*, and their mothers  Total of number of participants not stated. | 6 focus group discussions *(3 with mothers and 3 with unmarried women, consisting of 6-12 participants in each, using participatory tools such as body mapping and calendar methods),* 36 in-depth interviews *(26 with unmarried women, 10 with mothers)* | Qualitative | Throwing used menstrual materials into the street | Lack of disposal facilities in slum | Author inferred | Medium | Medium |
|  |  |  |  |  |  |  | Wrapping used menstrual materials in paper before disposing | Found menstrual blood dirty and did not want to wash cloths for reuse | Author inferred |  |  |
|  |  |  |  |  |  |  | Drying materials under clothes | Prevent people seeing the materials drying | Participant |  |  |
| Nalugya et a, 2020 (53) | Uganda | Low | Girls in school, parents, school staff | 450 Students *(baseline: 232 female and 218 male, aged 13-21, across 2 schools)*,  369 Students *(endline: 188 female and 181 male, aged 13-21, across 2 schools)*, 10 parents, 10 teachers | Baseline survey *(450 students)* and endline survey *(369 students),* 40 in-depth interviews *(10 parents, 10 teachers and 20 female students),* 8 focus group discussions *(4 at each school, with 6-10 participants each),* and WASH facility observation | Mixed-methods | Ensuring that used materials were not disposed of in an open place | Fears that if a dog ate the used pad, they may menstruate for life | Participant |  |  |
| Ndlovu and Bhala, 2016 (54) | Zimbabwe | Lower-middle | Women,  NGOs,  public sector,  religious institutions | 40 women, 30 key informants *(15 males and 5 females, including public sector departments, churches and NGOs)* | Focus group discussions *(with 40 women),* 30 structured survey responses *(from key informants)* | Mixed-methods | Throw into a pit latrine *(47%),* burning *(22%),* flush down toilets *(16%),* bury *(9%),* throw into bush *(6%)* | 85% of participants indicated a lack of proper disposal facilities. | Participants | Medium | Medium |
|  |  |  |  |  |  |  | Burning materials or washing before disposed | Fear of blood being seen and used for witchcraft | Participants |  |  |
| Oche et al, 2012 (55) | Nigeria | Lower-middle | Adolescent girls | 122 adolescent girls *(aged 15-20, across 4 schools)* | 122 self-administered questionnaires *(cross-sectional descriptive study)* | Qualitative | Predominantly, used menstrual materials were burnt, or wrapped up and thrown away | Beliefs that blood stained pads will attract witches/ others that could perform blood rituals | Author inferred | High | Low |
| Parker et al, 2014 (56) | Uganda | Low, and Displacement Camp  *(in and out of displacement camps)* | Girls in school, women, school staff, health workers | Up to 240 students *(aged 9-20, across 14 schools),* 8 Senior/head teachers, 9 health workers, up to 75 women (across 4 villages), up to 450 women (across 13 IDP settings)  Total of number of participants not stated. | 29 focus group discussions *(16 with girls in school, 10 with women in IDP camps, 3 with women in villages),* and 17 in-depth interviews *(8 with head teachers, 9 with health workers)* | Qualitative | Reusable materials were hung inside (sometimes also covered) | To hide from sight of family members *(can dry in village where there is space, but not IDP camp)* | Participants | Medium | Medium |
| Rajagopal and Mathur, 2017 (57) | India | Lower-middle | Adolescent girls | 270 adolescent girls *(130 school-going, 140 non-school-going, aged 10-20, across 5 schools)* | Surveys, focus group discussions and in-depth interviews  *(Number of each unspecified)* | Mixed-methods | Dried reusable materials under clothes, or in a secluded area | To be out of the way of ‘the male gaze’ | Author inferred | Medium | Medium |
|  |  |  |  |  |  |  | Wrapping used menstrual materials in newspaper or placing in plastic bags before disposing | Girls felt embarrassed if seen | Author inferred |  |  |
| Rajaraman et al, 2013 (58) | India | Lower-middle | Women | 48 women | 48 socio-economic questionnaires and in-depth interviews with each participant | Mixed-methods | Washing menstrual materials in public | Public toilets do not have adequate washing facilities | Participants | Medium | High |
|  |  |  |  |  |  |  | Avoiding washing and changing materials during the day | No facilities at work, so women have to just wait until arriving back home *(also leads to women missing work as they do not have spaces to change/wash)* | Participants |  |  |
| Ramathuba, 2015 (59) | South Africa | Upper-middle | Girls in school | 273 students *(female, aged 14-19, across 6 schools)* | 273 self-reported questionnaires | Quantitative | 63% threw into latrine, 33% into bins, and 3% flushed away | *(no specific reasoning given)* |  | Medium | Medium |
|  |  |  |  |  |  |  | 90% of students hid menstrual materials after washing, and the 7% that exposed them to sunlight did so under a washing cloth | Seen to be frowned upon if materials are left exposed, so they are hidden | Author inferred |  |  |
|  |  |  |  |  |  |  | Washing materials early in the morning | To clean them before male members of the family awoke | Author inferred |  |  |
| Rastogi et al, 2019 (60) | India | Lower-middle | Girls in school, parents, school staff | 187 students *(female, aged 13-15, across 4 schools),* parents and School staff  Total of number of participants not stated. | 187 Questionnaires, 18 focus group discussions *(8 with girls, 4 with mothers, 4 with teachers),* observation of facilities | Mixed-methods | All girls wrapped their used menstrual materials before disposing | To prevent materials being seen by others | Participants | Medium | Medium |
|  |  |  |  |  |  |  | *(no specific behaviour given)* | Girls were told disposing of used menstrual materials at home was not appropriate | Participants |  |  |
|  |  |  |  |  |  |  | Collecting all materials use throughout 1 period and then disposing of materials in one go | To prevent multiple trips, to decrease risk of being seen | Author inferred |  |  |
| Rheinländer et al, 2019 (61) | Ghana | Lower-middle | Girls in school, school staff | 33 students *(female, aged 14-23, across 2 schools),* 4 school staff (*female)* | 4 focus group discussions, observation of facilities *(through transect walks)*, 4 semi-structured in-depth interviews *(with school staff)* | Qualitative | Used menstrual materials were disposed of in open garage piles, bushy areas, under rocks on the beach, burned at home or into latrines | No waste management system on school premises | Author inferred | High | High |
|  |  |  |  |  |  |  | Using the bush as a toilet during menstruation | Girls can hide their blood by covering with sand | Participants |  |  |
|  |  |  |  |  |  |  | No girls used wastebins to dispose materials | Bins were in public spaces only, and girls wanted more privacy | Author inferred |  |  |
|  |  |  |  |  |  |  | Wrapping used menstrual materials in plastic covers/bags before throwing away/taking home to dispose | Added layer of secrecy so others could not identify waste | Participants |  |  |
| Rizvi and Ali, 2016 (62) | Pakistan | Lower-middle | Adolescent girls, | 20 adolescent girls *(aged 13-19, non-school-going)* | 3 focus group discussions *(each with 6-8 girls),* and 6 in-depth interviews with selected participants | Qualitative | Hiding material when drying | To make sure male family members did not see | Author inferred | Medium | Medium |
|  |  |  |  |  |  |  | Washing cloth before throwing away | It was a ‘sin’ to throw away unwashed materials | Participants |  |  |
|  |  |  |  |  |  |  | Placing used menstrual materials in bags for disposal | To avoid public exposure | Author inferred |  |  |
| Roxburgh et al, 2020 (63) | Malawi | Low | Women and university staff | 31 women *(aged 19-60+)* and 2 university staff | 35 interviews, 4 small focus groups (2-3 participants) and 2 key informant interviews *(with university staff)* | Qualitative | Hiding materials and home and then burning them when finished with, and avoiding disposing in the open, or leaving them in a bin | Fear of used materials *(menstrual cloth or used pad, or even stained underwear)* being used for witchcraft (ufiti) resulting in infertility or death *(predominantly older generations)* | Participants |  |  |
|  |  |  |  |  |  |  | Ensuring no water can be seen draining from the bathroom when washing materials | Ensuring blood cannot be seen by others | Author inferred |  |  |
|  |  |  |  |  |  |  | Burning used materials, but only early in the morning or late in evenings | Reduces chances of being seen by others, and provided a ‘safe solution’ by turning used materials to ash, protecting them from being used in ufiti, | Participants |  |  |
|  |  |  |  |  |  |  | Throwing used materials into pit latrines | Quick and discreet method of disposal, but worries of filling up latrines too quickly | Author inferred |  |  |
|  |  |  |  |  |  |  | Avoidance of using solid waste systems *(e.g. in bins or on local dumpsites)* | Seen as ‘immoral and disrespectful.’ as a public disposal method, and linked with fears of ufiti | Participants |  |  |
| Schmitt et al, 2021 (64) | Bangladesh | Low-middle *(refugee settlement)* | Women & girls, humanitarian response staff | 47 adolescent girls and women *(aged 15-35)*, 19 humanitarian response staff | 19 key informant interviews *(with humanitarian response staff),* 6 focus group discussions *(with 47 rohingya adolescent girls and women, 5-10 participants for each group),* and direct observations of 8 wash facilities | Mixed-methods | Throwing used materials into pit latrines and roadside drains | Lack of disposal facility | Author inferred |  |  |
|  |  |  |  |  |  |  | Burying used menstrual materials | For privacy reasons, and in some instances to lessen the risk of other people and evil spirits encountering menstrual materials which was seen to be ‘unclean and shameful’ | Author inferred |  |  |
| Schmitt et al, 2017 (65) | Lebanon, Myanmar | Lower-middle and upper-middle *(Displacement Camp)* | Women & girls, humanitarian staff | 117 women *(aged 19-49)*, 71 adolescent girls *(aged 14-18, 32 for focus group discussions and 39 for participatory mapping), 17 emergency response staff* | 17 Key-informant interviews with emergency response staff, focus group discussions with adolescent girls and women and participatory mapping with adolescent girls | Qualitative | Drying materials under clothes/ mattresses | To hide from men/boys | Author inferred | High | High |
|  |  |  |  |  |  |  | Throwing used menstrual materials into latrines | Perceived as convenient and discrete | Author inferred |  |  |
|  |  |  |  |  |  |  | Resistance from community to use bins placed in latrines | Cultural taboos and perceived humiliation from others being able to see used pads *(also men using female latrines)* | Author inferred |  |  |
|  |  |  |  |  |  |  | *(no specific behaviour given)* | Women and girls were not taught how to dispose of disposable pads | Participants |  |  |
| Scorgie et al, 2016 (66) | South Africa | Upper-middle | Women | 21 women *(aged 18-35, 17 of these completed the photovoice segment, then 7 of these then completed interviews)* | Workshops on photovoice and group discussions on MHM *(with all 17 participants),* then 7 in-depth interviews with photo-elicitation | Qualitative | Throwing used menstrual materials in the bin | Decreased chance of others seeing it | Participants | High | High |
|  |  |  |  |  |  |  | Wrapping materials in newspaper/toilet paper *(and then sometimes also in a bag)* before putting into latrine | Avoid others seeing used menstrual materials, especially if the latrine was beginning to fill up *(also women faced judgement from other women within the communities about how they managed their disposal)* | Participants |  |  |
|  |  |  |  |  |  |  | Avoiding disposal in communal bins | Worry that dogs will knock bins over and materials will be strewn | Participants |  |  |
|  |  |  |  |  |  |  | *(no specific behaviour given)* | Women knew not to flush disposable products, but all communities noted absence of MHM bins | Participants |  |  |
| Shah et al, 2019 (67) | Gambia | Low | Girls in school, mothers, school staff | 470 students *(427 female - aged 11-21, 43 male – aged 15-21),* 3 school staff, 5 mothers | 331 survey responses (from schoolgirls), 20 focus group discussions (9 with post-menarche girls, 5 with pre-menarche girls, 6 with boys) and 13 in-depth interviews *(3 school staff, 5 mothers, 5 boys)* | Mixed-methods | Drying materials  In a women’s only bathroom (72%), or either inside a bag or under a mattress (6%) | To make sure the materials remain hidden from others (especially males) whilst drying | Participants | High | High |
|  |  |  |  |  |  |  | Throwing used menstrual materials into the latrine | Beliefs around infertility if the material is burnt | Participants |  |  |
|  |  |  |  |  |  |  | *(no specific behaviour given)* | Hiding materials from other women to prevent curses | Participants |  |  |
| Sheoran et al, 2020 (68) | India | Lower-middle | Women & girls | 800 Women & girls *(aged 14-49)* | 800 structured questionnaires | Quantitative | 70.50% of women wrapped used materials in newspaper  before disposing | Taboos and shame around others seeing used materials causing embarrassment | Author inferred |  |  |
| Sivakami et al, 2019 (69) | India | Lower-Middle | Girls in school | 2564 students *(female, aged 12+ (average age 14), across 43 schools)* | 2564 close-ended surveys | Quantitative | Disposal of menstrual items into toilets, incinerators, bins, pits, by burning, or taking home | Only 27% of girls stated good disposal facilities were available on school grounds | Participants | High | Medium |
|  |  |  |  |  |  |  | *(no specific behaviour given)* | 38% of girls stated too little time in breaks to be able to change materials | Participants |  |  |
| Sommer et al, 2020 (70) | USA | High  *(homeless women)* | Women, government staff, shelter staff | 22 women *(aged 16-62)*, 3 government staff and 12 shelter staff | 22 interviews *(with homeless women),* 15 key informant interviews *(with staff at government agencies, shelters and service provider organizations)* and field audits of public toilets. | Qualitative | Wrapping used materials in toilet paper/tissue before disposing in a bin | Due to unspoken ‘menstrual etiquette’ | Author inferred |  |  |
|  |  |  |  |  |  |  | Flushing used materials down the toilet | Lack of disposal option for disposable materials | Author inferred |  |  |
| Sommer et al, 2015 (71) | Cambodia, Ghana, Ethiopia,  *(This study draws from a previous study from Tanzania – Sommer, 2009, for comparison purposes, this study is detailed below)* | Low and Lower-Middle | Adolescent girls, school staff,  parents,  health staff | ≈ 450 adolescent girls *(aged 16-19, both in and out of school, across the 3 countries),* school Staff,  parents,  health staff  Total of number of participants not stated. | Participatory activities (with the adolescent girls), observation of facilities, and key informant interviews *(with School Staff,*  *Parents,*  *Health Staff)* | Qualitative | *C= Cambodia, E = Ethiopia, G = Ghana, T = Tanzania* | | | Medium | High |
|  |  |  |  |  |  |  | *(no specific behaviour given)* | ‘Improper’ disposal would lead to infertility through animals touching materials (G&T) | Author inferred |  |  |
|  |  |  |  |  |  |  | Not allowing others to see materials when drying/washing (T) | Fears of infertility or being cursed if girls practised ‘improper disposal’ (T) | Author inferred |  |  |
|  |  |  |  |  |  |  | Drying materials under clothes, under beds or in dark corners | To reduce risk of other people seeing their drying materials (G&E) | Author inferred |  |  |
|  |  |  |  |  |  |  | *(no specific behaviour given)* | Hiding all aspects of menstruation to avoid physical and mental abuse (E&G) | Participants |  |  |
| Sommer, 2009 (72) | Tanzania | Lower-Middle | Adolescent girls | ≈ 140 adolescent girls *(aged 16-19)*  Total of number of participants not stated. | Participatory activities and 16 in-depth interviews |  | *(no specific behaviour given)* | Not feeling comfortable to change menstrual materials because there is no tap and no water available at school | Participants | High | Medium |
|  |  |  |  |  |  |  | Choosing not to throw menstrual material in the school toilets | Because toilets provided are flush toilets and know their materials cannot go in there | Participants |  |  |
| Tamiru et al, 2015 (73) | Ethiopia, South Sudan, Tanzania, Uganda, Zimbabwe | Low and Lower-Middle | Girls in school, boys in school, school Staff, community members | *Total of number of participants not stated.* | Structured interviews, and in-depth interviews, key informant interviews, focus group discussions, and observation of facilities  *(Quantity of activities not stated)* | Qualitative | Avoiding disposal in open spaces | If seen, fear of having witchcraft used against them | Participants | Medium | Medium |
|  |  |  |  |  |  |  | 47% threw used pads in the pit latrine, 16% flushed them down the toilets, 37% threw them into the forest | Lack of MM facilities *(99% do not have ‘appropriate MHM facilities’ in Tanzania, 81% in South Sudan, and 100% in Ethiopia)* | Author inferred |  |  |
| Tegegne and Sisay, 2014 (74) | Ethiopia | Low | Adolescent girls, school staff | At least 595 students *(female, aged 10-19),* 5 adolescent girls *(who had dropped out of school),* 4 teachers *(all female)*  Total of number of participants not stated. | In-depth interviews *(5 with girls who had dropped out of school, 4 with female teachers),* 4 focus group discussions *(with schoolgirls – unclear if overlap between those surveyed),* and 595 close-ended survey responses from schoolgirls | Mixed-methods | Choosing not to change/wash at school or avoiding school altogether during menstruation | Absence of separate gendered toilets that are ‘gender friendly’ | Participants | High | Medium |
|  |  |  |  |  |  |  | Disposing of used menstrual materials in latrines *(77%)*, or into open fields *(33%)* | *(no specific reasoning given)* |  |  |  |
| Trinies et al, 2015 (75) | Mali | Low | Girls in school, school staff | 26 students *(female, aged 12-17, across 8 schools),* 14 school staff *(4 female, 10 male, across 8 schools)* | 26 in-depth interviews *(with students)* and 14 key informant interviews *(with school staff)* | Qualitative | Reusable materials were dried inside (under beds, or in the latrine) | To remain private and to ensure concealment | Author inferred | High | High |
|  |  |  |  |  |  |  | Materials were disposed of by burning or throwing in latrines | Deemed ‘not appropriate’ for materials to go in a bin in case others saw – also to prohibit use of materials in form of witchcraft | Author inferred |  |  |
|  |  |  |  |  |  |  | Washing blood off used menstrual materials before disposing | *(no specific reasoning given)* |  |  |  |
| Umeora and Egwuatu, 2008 (76) | Nigeria | Lower-middle | Women | 1692 women *(female, aged 17-56)* | 1692 open-ended questionnaires and 12 in-depth interviews with women | Qualitative | Reusable materials were prominently dried in ‘hidden corners’ | *(no specific reasoning given)* |  | Medium | Medium |
|  |  |  |  |  |  |  | Materials were disposed of into latrines, refuse dumps, or buried | To hide menstrual blood from witches who would use it for witchcraft | Participants |  |  |
|  |  |  |  |  |  |  | Avoiding burning used menstrual materials | Beliefs that it could cause cancer/infertility | Participants |  |  |
| Visaria and Mishra, 2017 (77) | India | Lower-middle | Adolescent girls | 585 adolescent girls *(aged 12-19, split across the experiment area (406) and a control group (179), spanning rural and urban communities)*  Total number of participants not explicitly stated. | MHM Training Program, followed by a semi-structured questionnaire *(705 responses),* 589 interviews, and focus group discussions to assess relative knowledge | Mixed-methods | Reusable material was predominantly dried in sunlight *(>90% urban, 40-60% rural),* however, there were instances of girls having to dry materials inside | Lack of access to open space, issues with visibility *(especially in slums)* where materials may be visible to adjacent households | Author inferred | Medium | Medium |
|  |  |  |  |  |  |  | Compromised ability to change/wash materials frequently | Scarcity of water in rural areas | Author inferred |  |  |
| Wardell and Czerwinski, 2001 (78) | USA | High | Women | 33 women *(aged 22-27, on active duty or*  *reserve forces for the military)* | 33 in-depth interviews | Qualitative | Burying used menstrual materials, or packing up to take home | Lack of bins in sanitation facilities | Participants | Medium | High |
|  |  |  |  |  |  |  | *(no specific behaviour given)* | No MHM facilities, only ‘water and waste’ was managed | Participants |  |  |
| WaterAid Nepal, 2009 (79) | Nepal | Low | Girls in school | 204 students *(female, aged 12-20, across 4 schools)* | 204 self-administered structured close-ended questionnaire surveys*,* 4 focus group discussions *(each with 9-13 students, 47 students total),* and 5 in-depth interviews | Mixed-methods | Disposing of used menstrual materials in the latrine | 28% reported lack of a disposal system, 23% reported lack of water supply | Participants | High | Medium |
|  |  |  |  |  |  |  | Students in urban schools stated refraining from using school facilities at all | Due to facilities being ill-managed for washing/ disposing of used menstrual materials | Participants |  |  |
|  |  |  |  |  |  |  | Drying used menstrual materials outside (59%), but even so, in a separate space or under other items | So as not to be noticed by others | Participants |  |  |
|  |  |  |  |  |  |  | Disposing of used menstrual materials in latrines, streams, by burying and by burning | Lack of specific place to dispose of used menstrual materials | Participants |  |  |
| Wilbur et al, 2021 (80) | Nepal | Lower-middle | Women and carers | 20 women and girls *(aged 15-24)* and 13 carers | 23 in-depth interviews *(20 women and 13 carers),* photovoice *(3 women and 1 carer),* and observation of accessibility issues | Mixed-methods | Infrequent washing of reusable materials | Inaccessible WASH facilities *(particularly for menstruators with physical disabilities)* | Participants |  |  |
|  |  |  |  |  |  |  | Throwing used materials outside of the toilet/WASH facility *(visually impaired menstruator)* | No disposal option within the WASH facility that is easily located/found | Participants |  |  |
|  |  |  |  |  |  |  | Disposing of used materials down hillsides or in rivers | Less likely to be found by others | Author inferred |  |  |
| Wilson et al, 2014 (81) | Kenya | Lower-middle | Girls in School | 302 students *(female, unknown age across 10 schools)* | 302 questionnaires, training session on reusable materials, follow-up questionnaire | Quantitative | *(no specific behaviour given)* | 70% said it was better to dry materials outside, but 40% found this embarrassing | Author inferred | Medium | Low |
|  |  |  |  |  |  |  | 25% of girls were embarrassed to wash materials outside | Due to a high risk of been seen cleaning | Author inferred |  |  |
| Yeasmin et al, 2017 (82) | Bangladesh | Lower-middle | Women, men, waste emptiers | 43 women, 25 men, 14 children, 5 feacal sludge emptying operators, 4 waste bin emptiers, | 10 in-depth interviews *(5 with residents, 5 with feacal sludge emptying operators), 6 focus group discussions*  Pilot-tested intervention package, 24 in-depth interviews *(20 residents and 4 bin emptiers)* and 2 focus groups *(resident and landlords)*  *(All across the pre/post intervention phases)* | Qualitative | Occasional disposal of used menstrual materials in drains and ditches, more recently, into the latrine | To avoid disposing of used menstrual materials in public places | Author inferred | High | High |
|  |  |  |  |  |  |  | Wrapping of used menstrual materials in plastic before disposing into latrines | To hide the materials out of embarrassment | Author inferred |  |  |
|  |  |  |  |  |  |  | Avoidance of using provided bins | Feelings of disgust by men and women who can see used menstrual materials | Participants |  |  |
|  |  |  |  |  |  |  | Avoiding disposing of items ‘openly’ | Beliefs around causing diseases effecting male eyesight if used menstrual materials are seen | Participants |  |  |

*In some instances, explicit behaviours/reasoning were not identified in studies. These papers have still been included as there is either an explicit reason for not partaking in certain behaviours, a reason for not understanding the disposal/washing options available, or multiple other behaviours which do show related reasoning.

≈ Where only a school grade/level was given, this symbol represents the approximate age of the participants for use in comparison to other studies.

Reason given by:

*Participants* – there is a given quote in a paper that refers directly to a reasoning for a behaviour (direct reasoning)

*Author Inferred* – The author has either paraphrased information from participants, or is implying reasoning based on response (indirect reasoning)

Reference List:

1. Abera Y. Menarche, Menstruation related Problems and Practices among Adolescent High School Girls in Addis Ababa, 2003/04: Addis Ababa University; 2004.

2. Ahmmed F, Chowdhury MS, Helal SM. Sexual and reproductive health experiences of adolescent girls and women in marginalised communities in Bangladesh. Culture, health & sexuality. 2021:1-16.

3. Alda-Vidal C, Browne AL. Absorbents, practices, and infrastructures: Changing socio-material landscapes of menstrual waste in Lilongwe, Malawi. Social & Cultural Geography. 2021:1-21.

4. Alexander KT, Oduor C, Nyothach E, Laserson KF, Amek N, Eleveld A, et al. Water, sanitation and hygiene conditions in kenyan rural schools: Are schools meeting the needs of menstruating girls? Water. 2014;6(5):1453-66.

5. Asimah SA, Diabene PY, Wellington SNL. Menstrual hygiene management in Ghana: understanding the socio-cultural, economic, political factors, challenges and opportunities. Local action with international cooperation to improve and sustain water, sanitation and hygiene (WASH) services; Loughborough, UK. Loughborough: Loughborough University; 2017.

6. Averbach S, Sahin-Hodoglugil N, Musara P, Chipato T, van der Straten A. Duet® for menstrual protection: a feasibility study in Zimbabwe. Contraception. 2009;79(6):463-8.

7. Behera D, Sivakami M, Behera MR. Menarche and Menstruation in Rural Adolescent Girls in Maharashtra, India: A Qualitative Study. Journal of Health Management. 2015;17(4):510-9.

8. Bhattacharjee M. Menstrual Hygiene Management During Emergencies: A Study of Challenges Faced by Women and Adolescent Girls Living in Flood-prone Districts in Assam. Indian Journal of Gender Studies. 2019;26(1-2):96-107.

9. Caruso BA, Clasen TF, Hadley C, Yount KM, Haardörfer R, Rout M, et al. Understanding and defining sanitation insecurity: Women's gendered experiences of urination, defecation and menstruation in rural Odisha, India. BMJ Global Health. 2017;2(4).

10. Caruso BA, Dreibelbis R, Ogutu EA, Rheingans R. If you build it will they come? Factors influencing rural primary pupils' urination and defecation practices at school in western Kenya. Journal of Water Sanitation and Hygiene for Development. 2014;4(4):642-53.

11. Chakravarthy V, Rajagopal S, Joshi B. Does Menstrual Hygiene Management in Urban Slums Need a Different Lens? Challenges Faced by Women and Girls in Jaipur and Delhi. Indian Journal of Gender Studies. 2019;26(1-2):138-59.

12. Chinyama J, Chipungu J, Rudd C, Mwale M, Verstraete L, Sikamo C, et al. Menstrual hygiene management in rural schools of Zambia: A descriptive study of knowledge, experiences and challenges faced by schoolgirls. BMC Public Health. 2019;19(1).

13. Chothe V, Khubchandani J, Seabert D, Asalkar M, Rakshe S, Firke A, et al. Students' Perceptions and Doubts About Menstruation in Developing Countries: A Case Study From India. Health Promotion Practice. 2014;15(3):319-26.

14. Connolly S, Sommer M. Cambodian girls' recommendations for facilitating menstrual hygiene management in school. Journal of Water Sanitation and Hygiene for Development. 2013;3(4):612-22.

15. Coswosk ÉD, Neves-Silva P, Modena CM, Heller L. Having a toilet is not enough: The limitations in fulfilling the human rights to water and sanitation in a municipal school in Bahia, Brazil. BMC Public Health. 2019;19(1).

16. Crankshaw TL, Strauss M, Gumede B. Menstrual health management and schooling experience amongst female learners in Gauteng, South Africa: a mixed method study. Reproductive Health. 2020;17(1):1-15.

17. Crichton J, Okal J, Kabiru CW, Zulu EM. Emotional and Psychosocial Aspects of Menstrual Poverty in Resource-Poor Settings: A Qualitative Study of the Experiences of Adolescent Girls in an Informal Settlement in Nairobi. Health Care for Women International. 2013;34(10):891-916.

18. Crofts T, J., Fisher J, editors. Schoolgirls' experiences of managing menstrual hygiene in Uganda. 2011 35th WEDC International Conference - The Future of Water, Sanitation and Hygiene in Low-Income Countries: Innovation, Adaptation and Engagement in a Changing World; 2011; Loughborough.

19. Crofts T, Fisher J. Menstrual hygiene in Ugandan schools: An investigation of low-cost sanitary pads. Journal of Water Sanitation and Hygiene for Development. 2012;2(1):50-8.

20. Daniels GJ. Investigating Fear, Shyness, And Discomfort Related To Menstrual Hygiene Management In Rural Cambodia: Yale University; 2016.

21. Dhingra R, Kumar A, Kour M. Knowledge and practices related to menstruation among Tribal (Gujjar) adolescent girls. Studies on Ethno-Medicine. 2009;3(1):43-8.

22. Dolan CS, Ryus CR, Dopson S, Montgomery P, Scott L. A blind spot in girls' education: Menarche and its webs of exclusion in Ghana. Journal of International Development. 2014;26(5):643-57.

23. Ellis A, Haver J, Villasenor J, Parawan A, Venkatesh M, Freeman MC, et al. WASH challenges to girls' menstrual hygiene management in Metro Manila, Masbate, and South Central Mindanao, Philippines. Waterlines. 2016;35(3):306-23.

24. Enoch A, Nadutey A, Afful BF, Anokye R. Menstrual Hygiene Management: Challenges and Coping Strategies for Adolescents with Disabilities in the Kumasi Metro of Ghana. Disability, CBR & Inclusive Development. 2020;31(2):77-91.

25. Garikipati S, Boudot C. To Pad or Not to Pad: Towards Better Sanitary Care for Women in Indian Slums. Journal of International Development. 2017;29(1):32-51.

26. George AM, Leena KC. Experiences of the Women Using Menstrual Cup on Free Will - A Qualitative Inquiry. Online Journal of Health & Allied Sciences. 2020;19(3):1-4.

27. Girod C, Ellis A, Andes KL, Freeman MC, Caruso BA. Physical, Social, and Political Inequities Constraining Girls’ Menstrual Management at Schools in Informal Settlements of Nairobi, Kenya. Journal of Urban Health. 2017;94(6):835-46.

28. Gultie T, Hailu D, Workineh Y. Age of menarche and knowledge about menstrual hygiene management among adolescent school girls in amhara province, Ethiopia: Implication to health care workers & school teachers. PLoS One. 2014;9(9).

29. Habtegiorgis Y, Sisay T, Kloos H, Malede A, Yalew M, Arefaynie M, et al. Menstrual hygiene practices among high school girls in urban areas in Northeastern Ethiopia: A neglected issue in water, sanitation, and hygiene research. PLoS One. 2021;16(6):e0248825.

30. Hawkins A, Sharpe R, Spence K, Holmes N. Inappropriate flushing of menstrual sanitary products. Proceedings of the Institution of Civil Engineers-Water Management. 2019;172(4):163-9.

31. Hennegan J, Kibira SPS, Exum NG, Schwab KJ, Makumbi FE, Bukenya J. 'I do what a woman should do': a grounded theory study of women's menstrual experiences at work in Mukono District, Uganda. BMJ global health. 2020;5(11).

32. Hennegan J, Sol L. Confidence to manage menstruation at home and at school: findings from a cross-sectional survey of schoolgirls in rural Bangladesh. Culture, health & sexuality. 2020;22(2):146-65.

33. Hennegan J, Dolan C, Steinfield L, Montgomery P. A qualitative understanding of the effects of reusable sanitary pads and puberty education: Implications for future research and practice. Reproductive Health. 2017;14(1).

34. Hennegan J, Dolan C, Wu M, Scott L, Montgomery P. Measuring the prevalence and impact of poor menstrual hygiene management: A quantitative survey of schoolgirls in rural Uganda. BMJ Open. 2016;6(12).

35. Htun NN, Laosee O, Rattanapan C. Factors that influence menstrual hygiene management in adolescent girls in Mudon Township, Mon State, Myanmar. Journal of Health Science and Medical Research. 2021;39(3):207-17.

36. Jahan F, Nuruzzaman M, Sultana F, Mahfuz MT, Rahman M, Akhand F, et al. Piloting an acceptable and feasible menstrual hygiene products disposal system in urban and rural schools in Bangladesh. BMC Public Health. 2020;20(1):N.PAG-N.PAG.

37. Kambala C, Chinangwa A, Chipeta E, Torondel B, Morse T. Acceptability of menstrual products interventions for menstrual hygiene management among women and girls in Malawi. Reproductive Health. 2020;17(1):N.PAG-N.PAG.

38. Karibu K, Salami J, Azeez M, Azeez A. Onset of Menarche and Adolescent Menstrual Hygiene Practices in Semi-Urban Ibadan Community, Nigeria. Women's Reproductive Health. 2019;6(2):102-17.

39. Kemigisha E, Rai M, Mlahagwa W, Nyakato VN, Ivanova O. A qualitative study exploring menstruation experiences and practices among adolescent girls living in the nakivale refugee settlement, Uganda. International Journal of Environmental Research and Public Health. 2020;17(18):1-11.

40. Kohler P, Renggli S, Lüthi C. WASH and gender in health care facilities: The uncharted territory. Health Care for Women International. 2019;40(1):3-12.

41. Kumbeni MT, Otupiri E, Ziba FA. Menstrual hygiene among adolescent girls in junior high schools in rural northern Ghana. The Pan African medical journal. 2020;37:190.

42. Lahme AM, Stern R, Cooper D. Factors impacting on menstrual hygiene and their implications for health promotion. Global Health Promotion. 2018;25(1):54-62.

43. MacRae ER, Clasen T, Dasmohapatra M, Caruso BA. 'It's like a burden on the head': Redefining adequate menstrual hygiene management throughout women's varied life stages in Odisha, India. PLoS One. 2019;14(8):e0220114.

44. Mason L, Nyothach E, Alexander K, Odhiambo FO, Eleveld A, Vulule J, et al. 'We keep it secret so no one should know' - A qualitative study to explore young schoolgirls attitudes and experiences with menstruation in rural Western Kenya. PLoS One. 2013;8(11).

45. Maulingin-Gumbaketi E, Larkins S, Gunnarsson R, Rembeck G, Whittaker M, Redman-MacLaren M. 'Making of a Strong Woman': a constructivist grounded theory of the experiences of young women around menarche in Papua New Guinea. BMC Women's Health. 2021;21(1):144.

46. McHenga J, Phuma-Ngaiyaye E, Kasulo V. Do sanitation facilities in primary and secondary schools address Menstrual Hygiene needs? A study from Mzuzu City, Malawi. Physics and Chemistry of the Earth. 2020;115.

47. Miiro G, Rutakumwa R, Nakiyingi-Miiro J, Nakuya K, Musoke S, Namakula J, et al. Menstrual health and school absenteeism among adolescent girls in Uganda (MENISCUS): A feasibility study. BMC Women's Health. 2018;18(1).

48. Mohamed Y, Durrant K, Huggett C, Davis J, Macintyre A, Menu S, et al. A qualitative exploration of menstruation-related restrictive practices in Fiji, Solomon Islands and Papua New Guinea. PLoS One. 2018;13(12).

49. Mohammed S, Emil Larsen-Reindorf R. Menstrual knowledge, sociocultural restrictions, and barriers to menstrual hygiene management in Ghana: Evidence from a multi-method survey among adolescent schoolgirls and schoolboys. PLoS One. 2020;15(10 October).

50. Mohammed S, Larsen-Reindorf RE, Awal I. Menstrual Hygiene Management and School Absenteeism among Adolescents in Ghana: Results from a School-Based Cross-Sectional Study in a Rural Community. International journal of reproductive medicine. 2020;2020:6872491.

51. Mumtaz Z, Sivananthajothy P, Bhatti A, Sommer M. "How can we leave the traditions of our Baab Daada" socio-cultural structures and values driving menstrual hygiene management challenges in schools in Pakistan. Journal of Adolescence. 2019;76:152-61.

52. Muralidharan A. Constrained Choices? Menstrual Health and Hygiene Needs Among Adolescents in Mumbai Slums. Indian Journal of Gender Studies. 2019;26(1-2):12-39.

53. Nalugya R, Tanton C, Hytti L, Kansiime C, Nakuya K, Namirembe P, et al. Assessing the effectiveness of a comprehensive menstrual health intervention program in Ugandan schools (MENISCUS): process evaluation of a pilot intervention study. Pilot and feasibility studies. 2020;6:51.

54. Ndlovu E, Bhala E. Menstrual hygiene - A salient hazard in rural schools: A case of Masvingo district of Zimbabwe. Jamba: Journal of Disaster Risk Studies. 2016;8(2):1-8.

55. Oche M, O., Umar A, S., Gana G, J., Ango J, T. Menstrual health: the unmet needs of adolescent girls’ in Sokoto, Nigeria. Scientific Research and Essays. 2012;7(3):410-18.

56. Parker AH, Smith JA, Verdemato T, Cooke J, Webster J, Carter RC. Menstrual management: A neglected aspect of hygiene interventions. Disaster Prevention and Management. 2014;23(4):437-54.

57. Rajagopal S, Mathur K. ‘Breaking the silence around menstruation’: experiences of adolescent girls in an urban setting in India. Gender and Development. 2017;25(2):303-17.

58. Rajaraman D, Travasso SM, Heymann SJ. A qualitative study of access to sanitation amongst low-income working women in Bangalore, India. Journal of Water Sanitation and Hygiene for Development. 2013;3(3):432-40.

59. Ramathuba DU. Menstrual knowledge and practices of female adolescents in Vhembe district, Limpopo Province, South Africa. Curationis. 2015;38(1).

60. Rastogi S, Khanna A, Mathur P. Uncovering the challenges to menstrual health: Knowledge, attitudes and practices of adolescent girls in government schools of Delhi. Health Education Journal. 2019;78(7):839-50.

61. Rheinländer T, Gyapong M, Akpakli DE, Konradsen F. Secrets, shame and discipline: School girls' experiences of sanitation and menstrual hygiene management in a peri-urban community in Ghana. Health Care for Women International. 2019;40(1):13-32.

62. Rizvi N, Ali TS. Misconceptions and Mismanagement of Menstruation among Adolescents Girls who do not attend School in Pakistan. Journal of Asian Midwives. 2016;3(1):46–62.

63. Roxburgh H, Hampshire K, Kaliwo T, Tilley EA, Tilley EA, Oliver DM, et al. Power, danger, and secrecy-A socio-cultural examination of menstrual waste management in urban Malawi. PLoS One. 2020;15(6 June).

64. Schmitt ML, Wood OR, Clatworthy D, Rashid SF, Sommer M. Innovative strategies for providing menstruation-supportive water, sanitation and hygiene (WASH) facilities: learning from refugee camps in Cox's bazar, Bangladesh. Conflict and health. 2021;15(1):10.

65. Schmitt ML, Clatworthy D, Ratnayake R, Klaesener-Metzner N, Roesch E, Wheeler E, et al. Understanding the menstrual hygiene management challenges facing displaced girls and women: Findings from qualitative assessments in Myanmar and Lebanon. Conflict and Health. 2017;11(1).

66. Scorgie F, Foster J, Stadler J, Phiri T, Hoppenjans L, Rees H, et al. “Bitten By Shyness”: Menstrual Hygiene Management, Sanitation, and the Quest for Privacy in South Africa. Medical Anthropology. 2016;35(2):161-76.

67. Shah V, Nabwera HM, Sosseh F, Jallow Y, Comma E, Keita O, et al. A rite of passage: A mixed methodology study about knowledge, perceptions and practices of menstrual hygiene management in rural Gambia. BMC Public Health. 2019;19(1).

68. Sheoran P, Kaur S, Lata H, Sarin J. A descriptive study of menstrual hygiene practices among women at the rural area of Haryana. Journal of Nursing & Midwifery Sciences. 2020;7(4):269-73.

69. Sivakami M, van Eijk AM, Thakur H, Kakade N, Patil C, Shinde S, et al. Effect of menstruation on girls and their schooling, and facilitators of menstrual hygiene management in schools: Surveys in government schools in three states in India, 2015. Journal of Global Health. 2019;9(1).

70. Sommer M, Gruer C, Smith RC, Maroko A, Hopper K. Menstruation and homelessness: Challenges faced living in shelters and on the street in New York City. Health & Place. 2020;66.

71. Sommer M, Ackatia-Armah N, Connolly S, Smiles D. A comparison of the menstruation and education experiences of girls in Tanzania, Ghana, Cambodia and Ethiopia. Compare. 2015;45(4):589-609.

72. Sommer M. Ideologies of sexuality, menstruation and risk: girls' experiences of puberty and schooling in northern Tanzania. Culture, Health & Sexuality. 2009;11(4):383-98.

73. Tamiru S, Mamo K, Acidria P, Mushi R, Ali CS, Ndebele L. Towards a sustainable solution for school menstrual hygiene management: Cases of Ethiopia, Uganda, South-Sudan, Tanzania, and Zimbabwe. Waterlines. 2015;34(1):92-102.

74. Tegegne TK, Sisay MM. Menstrual hygiene management and school absenteeism among female adolescent students in Northeast Ethiopia. BMC Public Health. 2014;14(1).

75. Trinies V, Caruso BA, Sogoré A, Toubkiss J, Freeman MC. Uncovering the challenges to menstrual hygiene management in schools in Mali. Waterlines. 2015;34(1):31-40.

76. Umeora OU, Egwuatu VE. Menstruation in rural Igbo women of south east Nigeria: attitudes, beliefs and practices. African journal of reproductive health. 2008;12(1):109-15.

77. Visaria L, Mishra RN. Health Training Programme for Adolescent Girls: Some Lessons from India’s NGO Initiative. Journal of Health Management. 2017;19(1):97-108.

78. Wardell DW, Czerwinski B. A military challenge to managing feminine and personal hygiene. Journal of the American Academy of Nurse Practitioners. 2001;13(4):187-93.

79. WaterAid Nepal. Is Menstrual Hygiene and Management an Issue for Adolescent School Girls? A Comparative Study of Four Schools in Different Settings of Nepal. Kathmandu: WaterAid Nepal; 2009.

80. Wilbur J, Kayastha S, Mahon T, Torondel B, Hameed S, Sigdel A, et al. Qualitative study exploring the barriers to menstrual hygiene management faced by adolescents and young people with a disability, and their carers in the Kavrepalanchok district, Nepal. BMC Public Health. 2021;21(1):1-15.

81. Wilson E, Reeve J, Pitt A. Education. Period. Developing an acceptable and replicable menstrual hygiene intervention. Development in Practice. 2014;24(1):63-80.

82. Yeasmin F, Luby SP, Saxton RE, Nizame FA, Alam MU, Dutta NC, et al. Piloting a low-cost hardware intervention to reduce improper disposal of solid waste in communal toilets in low-income settlements in Dhaka, Bangladesh. BMC Public Health. 2017;17(1).
