# Supplementary material for: Drivers of menstrual material disposal and washing practices: A systematic review
Source: PLoS One. 2021 Dec 3;16(12):e0260472. doi: 10.1371/journal.pone.0260472 (PMC8641861; doi:10.1371/journal.pone.0260472)
Supplement: S2 Table — (DOCX) [file pone.0260472.s003.docx]

S2 Table: Quality appraisal of included studies.

Adapted from the EPPI-Centre Checklist detailed in Rees, R., Oliver, K., Woodman, J., Thomas, J. 2009. *Children’s views about obesity, body size, shape and weight: A systematic review*. EPPI-Centre, London: UK.

| **Colour Key** | **1. Rigor in sampling** | **2.Rigor in data collection** | **3.Rigor in analysis** | **4.Findings supported by the data** | **5.Breadth and depth of findings** | **6. Privileges perspectives of those who menstruate** |
| --- | --- | --- | --- | --- | --- | --- |
| **High** | More than 50 participants explicitly stated (per data collection activity)  ----AND----  Explicit recruitment strategy aligned to research question | Multiple methods of data collection AND evidence of pre-testing/iterating questions OR training of data collectors  ----OR----  One method of data collection AND evidence of pre-testing/iterating questions AND training of data collectors | Clear description of analysis | All/most author inferences supported AND quotes are clearly/moderately distinguishable between participants is used | A large proportion of the study refers to washing, reuse and/or disposal of menstrual materials | Balance between open and close ended responses OR clear illustrative quotes (both from menstruators) AND consent stated AND confidentiality measures stated |
| **Medium** | Between 20-49 participants explicitly stated (per data collection activity)  ----OR----  More than 50 participants implied (total figure not explicitly given)  ----OR----  20+ participants but recruitment strategy is only moderately aligned to research question | Multiple methods of data collection  ----OR----  One method of data collection and evidence of pre-testing/iterating questions OR training of data collectors | Some description of analysis | All author inferences supported, BUT quotes are not distinguishable between participants if used  ----OR----  Most author inferences supported AND quotes are clearly/moderately distinguishable between participants if used | A medium proportion of the study refers to washing, reuse and/or disposal of menstrual materials | Balance between open and close ended responses OR clear illustrative quotes OR solely quantitative data (all from menstruators) AND consent stated OR confidentiality measures stated |
| **Low** | Less than 20 participants (per data collection activity)  ----OR----  No clear recruitment strategy | Only one method of data collection AND no evidence of pre-testing/iterating questions, or training of data collectors | Little description of analysis | Most/some Author’s inferences supported BUT quotes are not clearly distinguishable between participants if used  ----OR----  Author’s inferences are not supported | A small proportion of the study refers to washing, reuse and/or disposal of menstrual materials | Few responses from menstruators  ----OR----  No responses from menstruators, but detailed observations  ----OR----  Neither consent nor confidentiality measures stated |
| **Inconclusive** | No sample explicitly stated | No data collection method stated | No analysis method stated | N/A | N/A | No responses from menstruators |
|  | **Overall trustworthiness, consider questions 1 to 4** | | | | **Overall usefulness/relevance, consider the review question and questions 5 and 6** | |
|  | High | | | | High | |
|  | Medium | | | | Medium | |
|  | Low | | | | Low | |

| Study ID | 1. Rigor in sampling | 2.Rigor in data collection | 3.Rigor in analysis | 4.Findings supported by the data | 5.Breadth and depth of findings | .6. Privileges perspectives of those who menstruate |
| --- | --- | --- | --- | --- | --- | --- |
| Abera, 2004 [1] | Large random sample (863 school students, unspecified number of school authorities), across 8 school settings (covering non-governmental and governmental) by stratified and random sampling | Training given to all data collectors, data was collected in various ways including focus groups discussions, questionnaires that were pre-tested and mixed-methods, and key-informant interviews lead by an interview guide. Questionnaire and interview guides provided in annex. | Method described to code and analyse questionnaire data, and qualitative findings were ‘discussed’ – no further explanation on deductive/inductive analysis. | Most author inferences supported by quantitative data No qualitative quotes referring to washing, reuse and/or disposal of menstrual material are used. | Range of questions were asked regarding menstruation, but few related to washing, reuse and/or disposal of menstrual materials | Balance between open and closed ended responses, students were put at ease through set times to ask questions about anything they did not understand, confidentiality was observed, Verbal informed consent given by participants, and several steps were taken to observe confidentiality. |
|  | Trustworthiness: Medium | | | | Relevance: Medium | |
| Ahmmed et al, 2021 [2] | Large sample size (89 married women, 42 adolescent girls, 18 elderly women, 3 traditional birth attendants, 3 medicine vendors) by purposive sampling | All data collectors had prior experience of collecting qualitative research. Multiple data collections methods: in-depth and key informant interviews, and focus groups discussions. | Analytical framework is described in detail, explanation of how analysis differed for qualitative and quantitative data. | Most author inferences supported by quantitative or qualitative data, but quotes are not identified as separate participants. | A small proportion of the study refers to washing, reuse and/or disposal of menstrual materials | Balance between open and close ended responses, all quotes anonymised, and oral consent given from all participants (and parental consent from those under 18), |
|  | Trustworthiness: High | | | | Relevance: Medium | |
| Alda-Vidal and Browne, 2021 [3] | Medium sample size (40 Women), by snowball sampling via the networks of field assistants | Data collectors were part of a network of local connections with previous experience supporting ethnographic research, and multiple data collections methods: semi-structured interviews and focus groups discussions | The project was broadly inspired by grounded theory approaches, and analysis was not led by a specific theoretical framework | All author inferences supported by quotes. All quotes identified as separate participants. | A large proportion of the study refers to washing, reuse and/or disposal of menstrual materials | Clear illustrative quotes, all quotes anonymised, and informed consent from participants given |
|  | Trustworthiness: High | | | | Relevance: High | |
| Alexander et al, 2014 [4] | Large sample size (62 headteachers/schools, 798 school facilities), by purposive sampling | Training given to all filed staff. Clear definitions of indicators used for the observation of facilities, and unannounced visits by field staff to get a representative view of facilities. No description of cross-sectional surveys given to head teachers. | Method describes how observational data was analysed, no methods for analysing survey data was given. | Most author inferences supported by observations or survey responses. But as all survey data was collected from head teachers only, no data is given from menstruators at the school. | Deep understanding of facilities and their current state, but thoughts/feelings not backed up by those using the facilities. | Both observational and qualitative data collected, but information was not given by those who menstruate. Consent given by all headteachers taking part. No information on confidentiality. |
|  | Trustworthiness: Medium | | | | Relevance: Low | |
| Asimah, et al, 2017 [5] | Large sample size (319 school students and 333 household heads), minimal detail on sampling methods | Multiple data collections methods: surveys, interviews, and focus groups discussions. Questions unlisted. | No detail on analysis of data. | All author inferences supported by paraphrased quotes or quantitative data. Paraphrased quotes are not identified as separate participants. | A large proportion of the study refers to washing, reuse and/or disposal of menstrual materials | Should be a balance of open and closed ended responses, but limited knowledge on methods prohibits confirmation of this. Both school pupils and households where sampled to gain deeper understanding. No information on consent. Quotes anonymised* to preserve confidentiality. |
|  | Trustworthiness: Medium | | | | Relevance: Medium | |
| Averbach, et al, 2009 [6] | Medium sample size (43 women), by convenience sampling | Multiple data collection methods: surveys and focus group discussions. FDGs lead by trained moderator with a discussion guide. All translations were read by multiple team members to ensure comprehension across languages. | All responses coded by two different coders to ensure consensus. Analysis lead by data. | All author inferences supported by quotes or quantitative data. All quotes identified as separate participants. | Study to primarily gauge interest in a product (Duet), but participants did not physically try the device: only acceptability was gauged. Participants did discuss multiple issues around management of menstruation generally. | Balance of open and closed ended responses. Informed consent given from all participants, no information on confidentiality. |
|  | Trustworthiness: High | | | | Relevance: Medium | |
| Behera et al, 2015 [7] | Medium sample size (32 adolescent girls), selected through convenience sampling | Data collected through focus group discussions. Questions to be asked were validated prior to study. | Qualitative data was transcribed, translated, and analysed through thematic analysis | All author inferences supported by quotes. Multiple illustrative quotes, all identified as separate participants | Large breadth of questions asked. Clear definition between findings. Only a small fraction of the study relates to washing, reuse and/or disposal of menstrual materials. | Clear illustrative quotes from menstruators throughout. Informed written consent given from all participants, no information on confidentiality. |
|  | Trustworthiness: High | | | | Relevance: Medium | |
| Bhattacharjee, 2019 [8] | Large sample size (84 Women and adolescent girls), but no information on sampling | Multiple data collection methods: interviews and focus group discussions. Questions unlisted. | No detail on analysis of data. | Most author inferences supported by quotes or quantitative data. All quotes identified as separate participants. | Several sections explicitly referred to washing, reuse and/or disposal of menstrual materials | Should be a balance of open and closed ended responses, but limited knowledge on methods prohibits confirmation of this. No information on confidentiality or consent. |
|  | Trustworthiness: Medium | | | | Relevance: Medium | |
| Caruso et al, 2017 [9] | Large sample size  (69 women for interviews and 46 women for discussions, by purposive sampling (chose a previous cluster randomised trial location)  *(unclear if there is overlap of women in interviews and FGDs)* | Multiple data collection methods: interviews and focus group discussions covering both close ended questions and open ended discussions. | Clear method of data analysis through thematic coding. | Most author inferences supported by quotes. Multiple illustrative quotes, moderately identifiable (they are unnamed, but labelled e.g. “married, FGD, no toilet” ) | Large proportion of the study refers to washing, reuse and/or disposal of menstrual materials, and does so with clear quotations and quantitative data. | Balance of open and closed ended responses. Oral consent given from all participants, confidentiality though unnamed quotes*. |
|  | Trustworthiness: High | | | | Relevance: High | |
| Caruso et al, 2014 [10] | Medium sample size (36 school students, 2 teachers) across 3 different schools), by purposive sampling (chose a previous cluster randomised trial location) | Multiple data collection methods: facility observation, interviews (with staff), and focus group discussions (with students). Author specified data saturation had been met regardless of Small sample size. | All data was transcribed for thematic analysis by primary author, two further authors then wrote memos of the textural description to inform overall analysis and results. | Most author inferences supported by quotes. Multiple illustrative quotes, all identified as separate participants. | Only a small proportion of the study is specifically related to washing, reuse and/or disposal of menstrual materials. | Head teachers provided overall consent for study ‘in loco parentis’, students gave oral consent. All quotes are unnamed, but highly detailed, so may be able to be identifiable (e.g. Girl Pupil 4, grade 7, age 14, School B FGD). |
|  | Trustworthiness: High | | | | Relevance: Medium | |
| Chakravarthy et al, 2019 [11] | Approximate Large sample size (45 focus groups with adolescent girl and women, and 20 NGO/Government officials), minimal detail on sampling methods | Multiple data collection methods: in-depth interviews and focus group discussions. | No detail on analysis of data. | Several author inferences are back up with quotations. Multiple illustrative quotes, all identified as separate participants. | A large proportion of the study refers to washing, reuse and/or disposal of menstrual materials. | Illustrative quotes from menstruators throughout. No details of participants consent. Confidentiality reached through anonymising participants named in quotes through pseudonyms. |
|  |  | Data draws from 3 studies (1 available report and 2 unpublished documents). There is therefore limited information available to understand the collection/analysis methods for all data used | |  |  |  |
|  | Trustworthiness: Low | | | | Relevance: Medium | |
| Chinyama et al, 2019 [12] | Large sample size (64 students for 8 FGDs), 12 students (for in-depth interviews), 7 teachers, 7 guardians, and 11 leaders, across 6 schools, by purposive sampling.  *(unclear if there is overlap of students in interviews and FGDs)* | Training given to all data collectors. Multiple data collection methods: key informant interview, in-depth interviews, and focus group discussions, (all conducted with field guides which are provided for the reader). All translations were checked by analysts to ensure accurate transcriptions. | All qualitative data was thematically analysed to identify inductive codes. | Most author inferences supported by quotes. Multiple illustrative quotes, all identified as separate participants. | Only a small proportion of the study refers to washing, reuse and/or disposal of menstrual materials. | Illustrative quotes from menstruators throughout. Written informed consent was gained from key informants and guardians, students under 18 gave assent. Several measures of confidentiality were used to secure data. |
|  | Trustworthiness: High | | | | Relevance: Medium | |
| Chothe et al, 2014 [13] | Large sample size (381 students) by convenience sampling. | Data collected through qualitative ethnographic research – students posed open-ended questions about MHH to paediatricians. | Questions were analysed descriptively and arranged into broad themes, were coded, and inferences were drawn by 3 investigators to ensure reproducibility. | Most author inferences supported by quotes. Multiple quotes, all identified as separate participants. | Only a small proportion of the study refers to washing, reuse and/or disposal of menstrual materials. | Clearly presented quotes from participants. ‘permission gained’ from school administration, but no specific mention of consent given. Confidentiality reached by not collecting participants’ names when collecting data. |
|  | Trustworthiness: High | | | | Relevance: Low | |
| Connolly and Sommer, 2013 [14] | Large sample size (146 students and 15 parents /teachers), by purposive sampling | Multiple data collection methods: ethnographic observation, key informant interviews, and participatory activities | Open coding used to develop themes and categories. Senior experts fed back on provisional findings before further coding was undertaken. | Some of author inferences supported by quotes/ observation, but quotes are not easily separated between participants (e.g. urban, grade 12) | Only a small proportion of the study refers to washing, reuse and/or disposal of menstrual materials. | Illustrative quotes from menstruators throughout Informed consent gained for all participants. Quotes anonymised to preserve confidentiality. |
|  | Trustworthiness: Medium | | | | Relevance: Medium | |
| Coswosk et al, 2019 [15] | Medium sample size (39 students, school principal and vice principal), by purposive sampling | Multiple data collection methods: participant observation, structured focus groups, and semi-structured interviews. Interview scripts in additional files for reader. | Data was systematised “using the normative content and the dimensions of the HRTW” (human rights to water and sanitation) | All author inferences supported by quotes. Multiple illustrative quotes, semi-separated participants (e.g. FG2 (focus group 2)) | Only a small proportion of the study refers to washing, reuse and/or disposal of menstrual materials | Illustrative quotes from menstruators throughout. Written informed consent was given by all participants. Anonymised quotes for confidentiality. |
|  | Trustworthiness: Medium | | | | Relevance: Medium | |
| Crankshaw et al, 2020 [16] | Large sample size (505+ students, 8 teachers, 9 mothers of students), across 10 schools, by purposive and convenience sampling | Multiple data collection methods: semi-structures interviews and quantitative self-administered surveys. | Transcripts were thematically analysed into themes and sub-themes, results were then coded and checked through comparison of multiple researchers to allow for accurate analysis. | Most author inferences supported by quotes or quantitative data. All quotes identified as separate participants. | Several sections explicitly referred to washing, reuse and/or disposal of menstrual materials | Balance of open and closed ended responses. All participants were given a consent form prior to beginning the research. All names anonymised when transcribing conversations, |
|  | Trustworthiness: High | | | | Relevance: High | |
| Crichton et al, 2013 [17] | Large sample size (87 students, 69 women, 5 teachers, 1 nurse), by purposive quota sampling | Training given to all data collectors. Multiple data collection methods: open-ended in-depth interviews*,* focus groups discussions, and key informant interviews. Guides for interviews were pre-tested and revised prior to this study. | Transcripts were thematically analysed into themes and sub-themes. Multiple authors involved in coding to ensure reproducibility. | All author inferences supported by quotes. Multiple illustrative quotes, all identified as separate participants | Only a small proportion of the study refers to washing, reuse and/or disposal of menstrual materials | Illustrative quotes from menstruators throughout. All participants gave informed consent. Several measures were taken to ensure confidentiality. |
|  | Trustworthiness: High | | | | Relevance: Medium | |
| Crofts and Fisher, 2011 & 2012 [18, 19] | Large sample size (134 students, 9 business leaders, 12 school staff), across 18 schools, by random and purposive sampling | Multiple data collection methods: Participatory activities, focus group discussions, key informant interviews, and observations of facilities | Qualitative data was filed according to themes, and graphically represented to observe patterns visually. Quantitative data was derived from participatory activities. | Some author inferences supported by quotes. Few quotes, but each is identified as separate participant. | A large proportion of the study refers to washing, reuse and/or disposal of menstrual materials | Clearly presented quotes from participants. No details of consent are stated. Participants assured of anonymity for confidentiality purposes. |
|  | Trustworthiness: Medium | | | | Relevance: High | |
| Daniels, 2016 [20] | Large sample size (165 participants (for interviews), 181 participants (for FDGs), including: girls, boys, Mothers, Fathers, and teachers , by purposive, random and convenience sampling  *(unclear if there is overlap of participants in interviews and FGDs – also breakdown of demographics is not given)* | Training given to all data collectors. Multiple data collection methods: structured focus groups and structured interviews | For qualitative analysis two coders worked collaboratively on a grounded theory by thematically coding transcripts inductively and deductively. | All author inferences supported by quotes. Multiple illustrative quotes, all identified as separate participants | Only a small proportion of the study refers to washing, reuse and/or disposal of menstrual materials | Illustrative quotes from menstruators throughout. Informed consent was given either by participants or by guardians. Anonymised quotes for confidentiality. |
|  | Trustworthiness: High | | | | Relevance: Medium | |
| Dhingra et al, 2009 [21] | Large sample size (200 adolescent girls), by random and snowball sampling | Multiple data collection methods: in-depth interviews, focus group discussions, and clinical observations. | Qualitative data analysed through content analysis. Quantitative data analysed through computing percentages and frequencies. | All author inferences supported by quotes. Multiple quotes, lack of identification of separate participants. | Only a small proportion of the study refers to washing, reuse and/or disposal of menstrual materials | Short quotes and figures used throughout. No information given on consent or confidentiality. |
|  | Trustworthiness: Medium | | | | Relevance: Low | |
| Dolan et al, 2014 [22] | Large sample size (99 adolescent girls (for interviews), 136 adolescent girls (for FGDs), 246 parents, 12 school staff (for interviews), 156 school staff (for FGDs/meetings), by purposive sampling  *(unclear if there is overlap of participants in interviews and FGDs* | Multiple data collection methods: semi-structured interviews, meetings, and focus group discussions before and after a non-randomised trial. | No detail on analysis of data. | Most author inferences supported by quotes or quantitative data. Few quotes, moderate identification as separate participants. | Only a small proportion of the study refers to washing, reuse and/or disposal of menstrual materials | Short quotes and figures used throughout. No information given on consent or confidentiality. |
|  | Trustworthiness: Medium | | | | Relevance: Low | |
| Enoch et al, 2020 [23] | Small sample size (18 adolescent girls), by purposive sampling | Data collectors had 2 weeks of training in qualitative data collection. Multiple data collection methods: in-depth interviews (with a guide) and focus group discussions | Qualitative data was thematically coded by three authors independently, then compared and resolved together | All author inferences supported by quotes. Multiple illustrative quotes, lack of identification of separate participants | Only a small proportion of the study refers to washing, reuse and/or disposal of menstrual materials | Illustrative quotes from menstruators throughout. Verbal consent from caregivers (for those under 18), no consent stated for those over 18. Anonymised quotes for confidentiality. |
|  | Trustworthiness: Medium | | | | Relevance: Medium | |
| Ellis et al, 2016 [24] | Large sample size (79 students), across 13 schools, by purposive sampling | Training given to all data collectors. Data collected through focus group discussions. Guide for the discussions was adapted for each differing context, piloted, and revised. | Qualitative data was coded by multiple authors to develop a codebook, and was then compared to observations of facilities to inform results. | All author inferences supported by quotes. Multiple illustrative quotes, all identified as separate participants | Several sections explicitly referred to washing, reuse and/or disposal of menstrual materials | Illustrative quotes from menstruators throughout. Informed consent was given from all parents/guardians, students also gave oral consent. Anonymised quotes for confidentiality. |
|  | Trustworthiness: High | | | | Relevance: High | |
| Garikipati and Boudot, 2017 [25] | Large sample size (150 women and adolescent girls), across 3 slum locations, by proportional and simple random sampling | Training given to all data collectors. Multiple data collection methods: semi structured interviews (questionnaires with categorical and open-ended answers), and observation of local shops (that sold menstrual materials) | No detail on analysis of data. | Most author inferences supported by quotes. Multiple illustrative quotes, lack of identification of separate participants. | Several sections explicitly referred to washing, reuse and/or disposal of menstrual materials | Balance of open and closed ended responses. Consent given from all participants. Unnamed quotes for confidentiality. |
|  | Trustworthiness: Medium | | | | Relevance: High | |
| George and Leena, 2020 [26] | Medium sample size (22 women), by snowball sampling | Data collected through open-ended interviews, the questions had been pre-tested with 2 women before beginning the full research | Six-phase inductive thematic analysis was conducted on the qualitative transcripts. | All author inferences supported by quotes. Multiple illustrative quotes, all identified as separate participants | Only a small proportion of the study refers to washing, reuse and/or disposal of menstrual materials | Illustrative quotes from menstruators throughout. Written informed consent was obtained from the participants. Anonymised quotes for confidentiality. |
|  | Trustworthiness: Medium | | | | Relevance: Medium | |
| Girod et al, 2017 [27] | Large sample size (51 Students approximately and 6 Headteachers) across 6 schools, by purposive sampling  *(total number of students not explicitly stated)* | Multiple data collection methods: focus group discussions, key informant interviews, observation of school facilities, and an anonymous question session | All transcripts were thematically analysed to create codes and subsequent webs to understand overlap and association amongst the data. | All author inferences supported by quotes. Multiple illustrative quotes, all identified as separate participants | Several sections explicitly referred to washing, reuse and/or disposal of menstrual materials | Illustrative quotes from menstruators throughout. Head teachers provided overall consent for study ‘in loco parentis’, all students and adults also provided verbal informed consent. Anonymised quotes for confidentiality. |
|  | Trustworthiness: High | | | | Relevance: High | |
| Gultie et al, 2014 [28] | Large sample size (492 students), identified though multistage and simple random sampling. | Training given to all data collectors. Data collected through structured self-administered questionnaire. The questionnaire was pre-tested on 50 students in a neighbouring district, then modified accordingly. | Data was coded and descriptive analysis was performed according to each deductive variable, and a bi-variate analysis was performed to further understand association of variable, multiple statistics were then drawn from the data. | All author inferences supported by quantitative data. No direct quotes. | Several sections explicitly referred to washing, reuse and/or disposal of menstrual materials | Solely quantitative data shown. Written informed consent from all participants. Confidentiality stated to be ‘maintained’. |
|  | Trustworthiness: High | | | | Relevance: Medium | |
| Habtegiorgis et al, 2021 [29] | Large sample size (536 students), across 5 schools, by proportional, and then simple random sampling | Data collected through a pre-tested survey. | Descriptive analysis and analytical statistics described in detail. | Most author inferences supported by quantitative data. No direct quotes. | Only a small proportion of the study refers to washing, reuse and/or disposal of menstrual materials | Solely quantitative data shown. Written consent given from all 18+ participants, and for those under 18 it was given by parents and guardians. Personal identifiers were not given to ensure confidentiality. |
|  | Trustworthiness: High | | | | Relevance: Medium | |
| Hawkins et al, 2019 [30] | Small sample size (10 women), by purposive snowballing | Data collected through semi-structured interviews. | Interviews were transcribed then underwent interpretative phenomenological analysis. | All author inferences supported by quotes or quantitative data. Multiple illustrative quotes, all identified as separate participants | A large proportion of the study refers to washing, reuse and/or disposal of menstrual materials | Balance of open and closed ended responses. No information on consent, but ethical approval obtained from the research ethics review panel at Sheffield Hallam University. Anonymised quotes for confidentiality. |
|  | Trustworthiness: Medium | | | | Relevance: High | |
| Hennegan et al, 2020 [31] | Medium sample size (35 women), by purposive sampling | Data collectors underwent 4 days of training. Data collected through interviews (with questions being piloted prior to research) | All transcripts underwent open, axial and selective coding, and constant comparison was facilitated. | Most author inferences supported by quotes or quantitative data. Multiple illustrative quotes, all identified as separate participants | Several sections explicitly referred to washing, reuse and/or disposal of menstrual materials | Balance of open and closed ended responses. All participants provided written informed consent. Anonymised quotes for confidentiality. |
|  | Trustworthiness: High | | | | Relevance: High | |
| Hennegan and Sol, 2020 [32] | Large sample size (1359 students), across 149 schools, by random sampling | Data collectors underwent 7 days of training. Data collected through surveys (full question list available). | Descriptive analysis and statistics, and sensitivity analysis described in detail. | Most author inferences supported by quantitative data. | Only a small proportion of the study refers to washing, reuse and/or disposal of menstrual materials | Solely quantitative data shown. Written consent from schools and participants. No information given on confidentiality. |
|  | Trustworthiness: High | | | | Relevance: Low | |
| Hennegan et al, 2017 [33] | Medium sample size (27 students) across 8 schools, by purposive sampling | Training given to all data collectors. Data collected through in-depth semi-structured interviews after a trial using a topic guide (detailed). Field notes and debrief sessions were also used to collect data alongside transcripts for contextualisation. | Transcripts were analysed using a framework approach, employing a thematic analytic approach. Framework analysis detailed. | All author inferences supported by quotes. Multiple illustrative quotes, all identified as separate participants | Several sections explicitly referred to washing, reuse and/or disposal of menstrual materials | Illustrative quotes from menstruators throughout. Participants and parent/guardians provided written consent for the trial, and verbal consent for the interviews. |
|  | Trustworthiness: High | | | | Relevance: High | |
| Hennegan et al, 2016 [34] | Large sample size (205 Students) across 8 schools, by purposive sampling | Training given to all data collectors. Data collected through a survey (following up a control trial on MHM), that had been pre-tested and adapted. Questions used in survey stated in paper. | Characteristics of data were detailed using descriptive statistics. Univariate logistic regressions assessed the relationships between several variables. | Most author inferences supported by quantitative data. | Several sections explicitly referred to washing, reuse and/or disposal of menstrual materials | Solely quantitative data shown. All participants and their parents/guardians provided written consent. No information given on confidentiality. |
|  | Trustworthiness: High | | | | Relevance: Medium | |
| Htun et al, 2021 [35] | Large sample size (410 adolescent girls), across 4 wards and 38 villages , by multi-stage sampling | Data collected through interviews, with a pilot study having been conducted with 30 participants prior. | Several descriptive statistics are identified. | Most author inferences supported by quantitative data. | Only a small proportion of the study refers to washing, reuse and/or disposal of menstrual materials | Solely quantitative data shown. Consent gained from parents and girls. Several confidentiality measures in place. |
|  | Trustworthiness: Medium | | | | Relevance: Low | |
| Jahan et al, 2020 [36] | Large sample size (419 students, 21 teachers, 28 janitors) by random sampling | Multiple data collection methods: focus group discussions, key informant interviews, in-depth interviews and observation of school facilities. | Deductive and inductive thematic analysis of qualitative data performed by several researchers to ensure validity of conclusions. | Most author inferences supported by quotes or quantitative data. Multiple illustrative quotes, all moderately identified as separate participants | Several sections explicitly referred to washing, reuse and/or disposal of menstrual materials | Balance of open and closed ended responses. Informed written consent from all participants (and parents where under 16). Anonymised quotes for confidentiality. |
|  | Trustworthiness: High | | | | Relevance: High | |
| Kambala et al, 2020 [37] | Large sample size (80 students, 61 women, 12 school staff, 6 community leaders, 8 community health workers, and 9 MHM service providers) by purposive sampling | Multiple data collection methods: focus group discussions and in-depth interviews | Content analysis performed on written transcripts, then conclusions supported through triangulation of data. | Most author inferences supported by quotes. Multiple illustrative quotes, all moderately identified as separate participants | Several sections explicitly referred to washing, reuse and/or disposal of menstrual materials | Illustrative quotes from menstruators throughout. All participants and their parents/guardians provided written consent. Anonymised quotes for confidentiality. |
|  | Trustworthiness: High | | | | Relevance: High | |
| Karibu et al, 2019 [38] | Large sample size (492 adolescent girls), by multistage and convenience sampling | Training given to all data collectors. Data collected through structured interviews (69-item questionnaire stated, but not detailed) | Data was coded and analysed at univariate and bivariate levels to create descriptive statistics. | Most author inferences supported by quantitative data. No direct quotes. | A large proportion of the study refers to washing, reuse and/or disposal of menstrual materials | Solely quantitative data shown. Consent was given by all participants. No information given on confidentiality. |
|  | Trustworthiness: High | | | | Relevance: Medium | |
| Kemigisha et al, 2020 [39] | Medium sample size (28 adolescent girls) by purposive sampling | Data collectors underwent 2 days of training Multiple data collection methods: focus group discussions and in-depth interviews | Thematic analysis was conducted on the qualitative data by 2 researchers to ensure validity of conclusions. | All author inferences supported by quotes. Multiple illustrative quotes, all moderately identified as separate participants | Only a small proportion of the study refers to washing, reuse and/or disposal of menstrual materials | Illustrative quotes from menstruators throughout. Informed consent given by those over 18, and by parents for those under 18. Anonymised quotes for confidentiality. |
|  | Trustworthiness: High | | | | Relevance: Medium | |
| Kohler et al, 2019 [40] | Large sample size (50 Indian participants and 40 Ugandan participants), across 4 hospitals, by purposive sampling  *(Both samples included in patients and staff)* | Multiple data collection methods: GALS Workshops, semi-structured interviews*,* and observation of facilities. The technical assessment for facility checks were pre-tested. | Analysed data collected in interviews, and used observation data to triangulate the user perception identified in qualitative work. | Most author inferences supported by paraphrased quotes. No direct quotes. | A large proportion of the study refers to washing, reuse and/or disposal of menstrual materials | Balance of open and closed ended responses recorded, but direct quotes not given in the paper. No information given on consent or confidentiality. |
|  | Trustworthiness: High | | | | Relevance: Medium | |
| Kumbeni et al, 2020 [41] | Large sample size (730 students) by simple random sampling | Training given to all data collectors. Multiple data collection methods: cross-sectional study (with pre-tested questions) and a WASH facility checklist | Bivariate analysis was performed on the quantitative data, and the scale system used for assessing levels of good practise are clearly explained. | Most author inferences supported by quantitative data. | Only a small proportion of the study refers to washing, reuse and/or disposal of menstrual materials | Solely quantitative data shown. Written informed consent was sought from all the respondents. Several steps were taken to ensure confidentiality. |
|  | Trustworthiness: High | | | | Relevance: Medium | |
| Lahme et al, 2018 [42] | Large sample size (51 students), across 3 schools, selected through purposive sampling | Data collected through focus group discussions | Thematic content analysis to code the data into 26 themes. | All author inferences supported by quotes. Multiple illustrative quotes, lack of identification of separate participants | Several sections explicitly referred to washing, reuse and/or disposal of menstrual materials | Illustrative quotes from menstruators throughout. Informed consent was given by all participants and parents/guardians. Participants assured of confidentiality. |
|  | Trustworthiness: Medium | | | | Relevance: High | |
| MacRae et al, 2019 [43] | Large sample size (114 women) across 14 communities by purposive sampling | Both data collectors had prior experience of qualitative data collection. Multiple data collection methods: focus group discussions and in-depth interviews | Transcripts were coded inductively and deductively, then underwent thematic analysis before being presented according to the JMP framework. | Most author inferences supported by quotes or quantitative data. Multiple illustrative quotes, all moderately identified as separate participants | Several sections explicitly referred to washing, reuse and/or disposal of menstrual materials | Balance of open and closed ended responses. Verbal consent given from all participants (as not to increase vulnerability of illiterate persons). Anonymised quotes for confidentiality. |
|  | Trustworthiness: High | | | | Relevance: High | |
| Mason et al, 2013 [44] | Large sample size (120 Students) across 6 schools, by random and proportionate sampling | Data collected through focus group discussions, using a guide with semi-structured topics (stated in paper). Transcripts were reviewed by the moderator after transcription to assess accuracy of translation. | Thematic content analysis was used by two authors to create the initial code frame. Each transcript was then coded by the lead researcher, who added inductive codes as they progressed. Another senior researcher then checked the coding frame for consistency and interpretation. | All author inferences supported by quotes. Multiple illustrative quotes, all identified as separate participants. | Only a small proportion of the study refers to washing, reuse and/or disposal of menstrual materials | Illustrative quotes from menstruators throughout. Written consent was given by all participants and parents/guardians. Anonymised quotes for confidentiality. |
|  | Trustworthiness: High | | | | Relevance: Medium | |
| Maulingin-Gumbaketi et al, 2021 [45] | Large sample size (98 women) across 4 provinces, by purposive and theoretical sampling | Multiple data collection methods: focus group discussions and semi-structured interviews (interview guide available). | Data was inductively analysed using initial, intermediate and advanced coding, then iterative and constant comparative methods were used to ensure validity of conclusions. | All author inferences supported by quotes. Multiple illustrative quotes, all identified as separate participants | Only a small proportion of the study refers to washing, reuse and/or disposal of menstrual materials | Illustrative quotes from menstruators throughout. Consent was given by all participants (literate participants gave written consent, and illiterate participants provided fingerprints). Anonymised quotes for confidentiality. |
|  | Trustworthiness: High | | | | Relevance: Medium | |
| McHenga et al, 2020 [46] | Large sample size (228 students and 22 school staff), by systematic Random Sampling | Multiple data collection methods: in-depth interviews, focus group discussions, key-informant interviews, and WASH facility observation. | Clear description of ratings for the state of WASH facilities. Descriptive statistics used for quantitative data and content analysis was used for qualitative data. | All author inferences supported by quotes or quantitative data. Multiple illustrative quotes, moderate identification as separate participants. | A large proportion of the study refers to washing, reuse and/or disposal of menstrual materials | Balance of open and closed ended responses. Informed and written consent was obtained from all participants. Anonymised quotes for confidentiality. |
|  | Trustworthiness: High | | | | Relevance: High | |
| Miiro et al, 2018 [47] | Large sample size (562 students, 11 teachers, 2 municipality officials, 10 parents), across 4 schools, by purposive sampling | Training given to all data collectors. Multiple data collection methods: in-depth interviews, group interviews, key-informant interviews, WASH facility observation, written diary entries and a questionnaire | Thematic content analysis was used by 5 individuals to test inter-rater reliability between transcripts. Themes were then discussed iteratively to ensure accuracy between researchers. Several statistical tests are detailed that were performed on the qualitative data. | All author inferences supported by quotes or quantitative data. Multiple illustrative quotes, all identified as separate participants. | Several sections explicitly referred to washing, reuse and/or disposal of menstrual materials | Balance of open and closed ended responses. Written content was given by all participants and their parents/guardians. Multiple steps to ensure confidentiality taken. |
|  | Trustworthiness: High | | | | Relevance: Medium | |
| Mohamed et al, 2018 [48] | Large sample size (54 Girls in school, 43 Adolescent girls, 118 women, 51 men, 8 school staff, and 34 community members) across 3 countries by purposive sampling | Training given to all data collectors. Multiple data collection methods: focus group discussions, in-depth interviews and key informant interviews. Question guides were reviewed and finalised in country before being used. | Authors used field notes alongside data collected to inform context. One author devised the coding frame for analysis, but this was then tested by two other authors to ensure reliability. Thematic analysis was then carried out on all the qualitative data. | All author inferences supported by quotes or quantitative data. Multiple illustrative quotes, moderate identification as separate participants. | Several sections explicitly referred to washing, reuse and/or disposal of menstrual materials | Illustrative quotes from menstruators throughout. Both written consent from parents/guardians as well as verbal assent was given from all participants. Multiple steps to ensure confidentiality taken. |
|  | Trustworthiness: High | | | | Relevance: High | |
| Mohammed et al, 2020 [49] and Mohammed and Larsen-Reindorf, 2020 [50] | Large sample size (280 Students and 5 head teachers) across 3 schools by simple random sampling | Training given to all data collectors. Multiple data collection methods: quantitative questionnaires (pre-tested on 30 adolescents), focus group discussions, and key informant interviews. | Clear description of point-system allocated to answers in the questionnaires. Binary logistic regression was used on data, followed by univariable analysis. Qualitative data was coded then discussed by several individuals to ensure understanding of the findings. | All author inferences supported by quotes or quantitative data. Multiple illustrative quotes, moderate identification as separate participants. | Only a small proportion of the study refers to washing, reuse and/or disposal of menstrual materials | Balance of open and closed ended responses. Written consent was given for those over the age of consent, for those under schools signed on their behalf. Anonymised quotes for confidentiality. |
|  | Trustworthiness: High | | | | Relevance: Medium | |
| Mumtaz et al, 2019 [51] | Large sample size (312 students, 15 mothers, 11 female school teachers, 9 health care providers, 5 local religious leaders and 1 scholar) by convenience sampling. | Multiple data collection methods: participatory activities and informal discussions, observations of School WASH facilities, and key informant interviews | Qualitative data was analysed using a latent content analysis approach, and was coded by 2 researchers to ensure accurate categorisation of themes. | All author inferences supported by quotes. Multiple illustrative quotes, all identified as separate participants. | A large proportion of the study refers to washing, reuse and/or disposal of menstrual materials | Illustrative quotes from menstruators throughout. Verbal assent given from participants. No names recorded for confidentiality. |
|  | Trustworthiness: High | | | | Relevance: High | |
| Muralidharan, 2019 [52] | Large sample size (up to 72), by purposive sampling  *(total number of participants not explicitly stated)* | Multiple data collection methods: focus group discussions and in-depth interviews. Focus group discussions informed the design of the interview questions. | Thematic content analysis was carried out alongside analytic memos were maintained. | All author inferences supported by quotes. Multiple illustrative quotes, moderate identification as separate participants. | Only a small proportion of the study refers to washing, reuse and/or disposal of menstrual materials | Illustrative quotes from menstruators throughout. Consent given from all parents/guardians and participants. Anonymised quotes for confidentiality. |
|  | Trustworthiness: Medium | | | | Relevance: Medium | |
| Nalugya et al, 2020 [53] | Large sample size (450 Students, 10 parents, 10 teachers) across 2 schools by purposive sampling | Independent educational consultant led the training of staff members. Multiple data collection methods: baseline and endline surveys, in-depth interviews, focus group discussions and WASH facility observation | All transcripts were reviewed for translation accuracy prior to analysis. A coding framework was developed for qualitative data, and findings were checked for consistency across several researchers. | All author inferences supported by quotes or quantitative data. Multiple illustrative quotes, all identified as separate participants. | Only a small proportion of the study refers to washing, reuse and/or disposal of menstrual materials | Balance of open and closed ended responses. Written informed consent was sought from students aged over 18 years, and from parents/ caretakers of those aged under 18 years. Multiple steps to ensure confidentiality taken. |
|  | Trustworthiness: High | | | | Relevance: Medium | |
| Ndlovu and Bhala, 2016 [54] | Medium sample size (40 women, 30 key informants) by purposive sampling | Multiple data collection methods: focus group discussions and structured survey responses | Thematic analysis was carried out on qualitative data as well as graphical information presented for ease of interpretation. | All author inferences supported by quantitative data. No direct quotes. | Several sections explicitly referred to washing, reuse and/or disposal of menstrual materials | Solely quantitative data shown. Informed verbal consent was obtained from all participants. Multiple steps to ensure confidentiality taken. |
|  | Trustworthiness: Medium | | | | Relevance: Medium | |
| Oche et al, 2012 [55] | Large sample size (122 Adolescent girls) across 4 schools, by simple random and systematic sampling | Data collected through a cross-sectional descriptive study through self-administered questionnaires. Questionnaires were pre-tested. | Questionnaire responses were marked corresponding to accuracy of knowledge on specified topics. Assessment of levels of knowledge specified. Several statistical tests carried out on data. | Some author inferences supported by quantitative data. No direct quotes. | Only a small proportion of the study refers to washing, reuse and/or disposal of menstrual materials | Solely quantitative data shown. Verbal consent by participants. . No information given on confidentiality. |
|  | Trustworthiness: High | | | | Relevance: Low | |
| Parker et al, 2014 [56] | Large sample size (up to 240 students, up to 75 women, up to 450 women in IDP camps, 8 Senior/head teachers, 9 health workers) across 14 schools, 4 villages and 13 IDP camps, minimal detail on sampling methods of specific participants (purposive sampling for specifying schools, villages and camps)  *(total number of participants not explicitly stated)* | Multiple data collection methods: focus group discussions and in-depth interviews | No detail on analysis of data. | Most author inferences supported by quotes. Multiple illustrative quotes, all identified as separate participants. | Several sections explicitly referred to washing, reuse and/or disposal of menstrual materials | Illustrative quotes from menstruators throughout. Consent was “sought”. No names were recorded to ensure confidentiality. |
|  | Trustworthiness: Medium | | | | Relevance: Medium | |
| Rajagopal and Mathur, 2017 [57] | Large sample size (270 adolescent girls) across 5 schools, minimal detail on sampling methods of specific participants (purposive sampling for specifying schools) | Multiple data collection methods: surveys, focus group discussions and in-depth interviews | No detail on analysis of data. | Most author inferences supported by quotes. Multiple illustrative quotes, all identified as separate participants. | Several sections explicitly referred to washing, reuse and/or disposal of menstrual materials | Illustrative quotes from menstruators throughout. No information given on consent or confidentiality (names used to identify participants, no information on whether these are real names or aliases). |
|  | Trustworthiness: Medium | | | | Relevance: Medium | |
| Rajaraman et al, 2013 [58] | Medium sample size (48 women), by quota sampling | Multiple data collection methods: short socio-economic questionnaire and in-depth interview with each participant | Interview transcripts coded thematically. | All author inferences supported by quotes. Multiple illustrative quotes, all identified as separate participants. | A large proportion of the study refers to washing, reuse and/or disposal of menstrual materials | Illustrative quotes from menstruators throughout. Written consent given by all participants. Aliases assigned to participants for confidentiality. |
|  | Trustworthiness: Medium | | | | Relevance: High | |
| Ramathuba, 2015 [59] | Large sample size (273 students), across 6 schools, by systemic and convenience sampling | Data collected through self-reported questionnaires | Data was analysed by computing frequencies and percentages on statistical packages | Most author inferences supported by quantitative data. No direct quotes. | A large proportion of the study refers to washing, reuse and/or disposal of menstrual materials | Solely quantitative data shown. Verbal and written consent given by all participants. Confidentiality achieved through assigning numbers (not names) to survey transcripts. |
|  | Trustworthiness: Medium | | | | Relevance: Medium | |
| Rastogi et al, 2019 [60] | Large sample size (187 students*,* Parents and School Staff), across 4 schools, by purposive and random sampling | Multiple data collection methods: questionnaires, focus group discussions*,* and observation of facilities. Questionnaire was pre-tested. | Little description of analysis | Most author inferences supported by quotes. Multiple illustrative quotes, lack of identification of separate participants | Several sections explicitly referred to washing, reuse and/or disposal of menstrual materials | Balance of open and closed ended responses. Parental/guardian consent was obtained. Confidentiality through unnamed quotes “one girl said…” |
|  | Trustworthiness: Medium | | | | Relevance: Medium | |
| Rheinländer et al, 2019 [61] | Medium sample size (33 students, 4 school staff), across 2 schools, by purposive and convenience sampling | Training given to all data collectors. Multiple data collection methods: focus group discussions, observation of facilities *(through transect walks)*, semi-structured in-depth interviews | Four step systematic content analysis (themes, codes, re-contextualising, triangulating data). | All author inferences supported by quotes. Multiple illustrative quotes, lack of identification of separate participants | A large proportion of the study refers to washing, reuse and/or disposal of menstrual materials | Illustrative quotes from menstruators throughout. Written consent from all 18+, written assent by all under 18 with adult witnesses. Anonymised quotes for confidentiality. |
|  | Trustworthiness: High | | | | Relevance: High | |
| Rizvi and Ali, 2016 [62] | Medium sample size (20 adolescent girls), by purposive sampling | Multiple data collection methods: focus group discussions and in-depth interviews | Categories shared with participants to confirm meaning. Qualitative analysis of data. | All author inferences supported by quotes. Multiple illustrative quotes, moderate identification as separate participants. | Only a small proportion of the study refers to washing, reuse and/or disposal of menstrual materials | Illustrative quotes from menstruators throughout. Verbal consent given from participants and mothers. Anonymised quotes for confidentiality. |
|  | Trustworthiness: Medium | | | | Relevance: Medium | |
| Roxburgh et al, 2020 [63] | Medium sample (31 women 2 university staff) by stratified and snowball sampling | Multiple data collection methods: in-depth interviews, focus groups and key informant interviews | Analysis was conducted by inductive thematic coding, and several researchers compared emerging themes to ensure validity across conclusions. | Most author inferences supported by quotes. Multiple illustrative quotes, all identified as separate participants. | Several sections explicitly referred to washing, reuse and/or disposal of menstrual materials | Illustrative quotes from menstruators throughout. All participants provided informed, written consent. Pseudonyms are used to ensure anonymity. |
|  | Trustworthiness: Medium | | | | Relevance: Medium | |
| Schmitt et al, 2021 [64] | Large sample size (47 adolescent girls and women and 19 humanitarian response staff) by purposive sampling | Multiple data collection methods: key informant interviews, focus group discussions and direct observations of wash facilities | Qualitative data underwent thematic analysis, which was cross referenced between researchers to ensure valid conclusions. | All author inferences supported by quotes. Multiple illustrative quotes, lack of identification of separate participants | Only a small proportion of the study refers to washing, reuse and/or disposal of menstrual materials | Illustrative quotes from menstruators throughout. All participants provided oral informed consent. Confidentiality through unnamed quotes “one girl said…”one Rohingya woman”. |
|  | Trustworthiness: High | | | | Relevance: Medium | |
| Schmitt et al, 2017 [65] | Large sample size (117 women, 71 adolescent girls, 17 emergency response staff) by purposive sampling | Training given to all data collectors. Multiple data collection methods: key-informant interviews, focus group discussions and participatory mapping | Two researchers reviewed all qualitative data through 4 steps of systematic text condensation (explained in text). Themes shared amongst whole research team for additional validation. | All author inferences supported by quotes. Multiple illustrative quotes, moderate identification as separate participants. | Several sections explicitly referred to washing, reuse and/or disposal of menstrual materials | Illustrative quotes from menstruators throughout. Oral informed consent given by all participants. Anonymised quotes for confidentiality. |
|  | Trustworthiness: High | | | | Relevance: High | |
| Scorgie et al, 2016 [66] | Medium sample size (21 women), by purposive and quota sampling | Training given to all data collectors (participants). Multiple data collection methods: workshops on photovoice, focus group discussions and in-depth interviews with photo-elicitation | Interview transcripts were manually coded using a grounded theory approach. Data extraction sheets developed using key themes, then reviewed by three researchers to validate analysis. | All author inferences supported by quotes. Multiple illustrative quotes, moderate identification as separate participants | A large proportion of the study refers to washing, reuse and/or disposal of menstrual materials | Illustrative quotes from menstruators throughout. Written informed consent by all participants. No information given on confidentiality (names used to identify participants, no information on whether these are real names or aliases). |
|  | Trustworthiness: High | | | | Relevance: High | |
| Shah et al, 2019 [67] | Large sample size (470 students, 3 school staff, 5 mothers), by convenience and purposive sampling | Training given to all data collectors. Multiple data collection methods: survey responses, focus group discussions and in-depth interviews. Survey modelled from the qualitative data. Qualitative and quantitative tools were pretested. | Section of transcripts sent to independent translator to ensure accuracy. Inductive content analysis was conducted on qualitative data – codes then compared between researchers to validate analysis. Several statistics tests were conducted on quantitative data. | All author inferences supported by quotes and quantitative data. Multiple illustrative quotes, moderate identification as separate participants | Several sections explicitly referred to washing, reuse and/or disposal of menstrual materials | Illustrative quotes from menstruators throughout. Written consent from participants and parents/guardians. Anonymised quotes for confidentiality. |
|  | Trustworthiness: High | | | | Relevance: High | |
| Sheoran et al, 2020 [68] | Large sample size (800 Women & girls) by convenience sampling. | Data collected through structured questionnaires (which were tested for reliability of conclusions by several sector experts). | Frequency percentage distribution was used to analyse the practices. | All author inferences supported by quantitative data. No direct quotes. | Only a small proportion of the study refers to washing, reuse and/or disposal of menstrual materials | Solely quantitative data shown. Written informed consent was obtained from the participants. Confidentiality was “maintained”. |
|  | Trustworthiness: High | | | | Relevance: Medium | |
| Sivakami et al, 2019[69] | Large sample size (2564 students), across 43 schools, by multi-level stratified, random and purposive sampling | Training given to all data collectors. Data collected through close-ended surveys. Surveys were pre-tested. | Significant differences explored at state level and within states. Several statistical tests were carried out, and exclusion criteria is listed. | All author inferences supported by quantitative data. . No direct quotes. | Several sections explicitly referred to washing, reuse and/or disposal of menstrual materials | Solely quantitative data shown. Written informed consent from parents/guardians and assent from participants gained. No information given on confidentiality |
|  | Trustworthiness: High | | | | Relevance: Medium | |
| Sommer et al, 2020 [70] | Medium sample size (22 women, 3 government staff and 12 shelter staff) by purposive and convenience sampling | Training given to all data collectors. Multiple data collection methods: in-depth interviews, key informant interviews and field audits of public toilets. | Thematic cross-case analysis was performed by 2 researchers, then shared for discussion with the full team to ensure validity of conclusions. | Most author inferences supported by quotes. Multiple illustrative quotes, all identified as separate participants. | Only a small proportion of the study refers to washing, reuse and/or disposal of menstrual materials | Illustrative quotes from menstruators throughout. Informed consent was provided by all participants. Multiple steps to ensure confidentiality taken. |
|  | Trustworthiness: High | | | | Relevance: Medium | |
| Sommer et al, 2015 [71] | Large sample size (approximately 450 Adolescent girls, School Staff, Parents, Health Staff) across 3 countries, minimal detail on sampling methods (probable purposive)  *(total number of participants not explicitly stated)* | Multiple data collection methods: participatory activities, observation of facilities, and key informant interviews | Constant comparative analysis to analyse multiple data sources. Feedback on preliminary findings to integrate local expert insight into research. | Some author inferences supported by quotes. Multiple illustrative quotes, lack of identification of separate participants | Several sections explicitly referred to washing, reuse and/or disposal of menstrual materials | Illustrative quotes from menstruators throughout. Informed consent from all participants. No names were recorded to ensure confidentiality. |
|  | Trustworthiness: Medium | | | | Relevance: High | |
| Sommer, 2009 [72] | Large sample size (approximately 140 Adolescent girls), minimal detail on sampling methods (probable purposive)  *(total number of participants not explicitly stated)* | Multiple data collection methods: participatory activities and in-depth interviews | Systematic analysis through generating hypothesis through fieldnotes, then coding of narratives. Three main themes emerged (detailed in text). | All author inferences supported by quotes. Multiple illustrative quotes, all identified as separate participants. | Only a small proportion of the study refers to washing, reuse and/or disposal of menstrual materials | Illustrative quotes from menstruators throughout. Informed consent given by all participants. No names were recorded to ensure confidentiality. |
|  | Trustworthiness: High | | | | Relevance: Medium | |
| Tamiru et al, 2015 [73] | Presumed medium/Large sample size, cross 5 countries, by purposive and convenience sampling  *(total number of participants not explicitly stated)* | Training given to all data collectors. Multiple data collection methods: structured interviews, in-depth interviews, key informant interviews, focus group discussions, and observation of facilities | Little description of analysis | Most author inferences supported by quantitative data. . No direct quotes. | Several sections explicitly referred to washing, reuse and/or disposal of menstrual materials | Solely quantitative data shown. Informed verbal consent by head of school and all participants. Anonymised quotes for confidentiality. |
|  | Trustworthiness: Medium | | | | Relevance: Medium | |
| Tegegne and Sisay, 2014 [74] | Large sample size (At least 595 students, 5 girls who had dropped out of school, 4 teachers), across 7 schools by multistage (stratified random and simple random) sampling | Training given to all data collectors. Multiple data collection methods: in-depth interviews*,* focus group discussions*,* and close-ended survey responses | Bivariate and multivariate models were run on quantitative data to assess relationships between all variables. Specification of how student’s menstrual knowledge was scored. Qualitative data was analysed by two researchers to discuss discrepancies and ensure accurate interpretation. | All author inferences supported by quantitative data. . No direct quotes. | Several sections explicitly referred to washing, reuse and/or disposal of menstrual materials | Solely quantitative data shown. Verbal consent given by participants’ parents if under 18. Confidentiality maintained by removing personal identifiers from questionnaires. |
|  | Trustworthiness: High | | | | Relevance: Medium | |
| Trinies et al, 2015 [75] | Medium sample size (26 students and 14 school staff), across 8 schools, by purposive sampling | Training given to all data collectors. Multiple data collection methods: in-depth interviews and key informant interviews | Interviews were analysed using thematic analysis. Codes were modified and applied from an existing framework (detailed). | Most author inferences supported by quotes. Multiple illustrative quotes, moderate identification as separate participants | Several sections explicitly referred to washing, reuse and/or disposal of menstrual materials | Illustrative quotes from menstruators throughout. Written consent from mothers, oral consent from all participants. Anonymised quotes for confidentiality. |
|  | Trustworthiness: High | | | | Relevance: High | |
| Umeora and Egwuatu, 2008 [76] | Large sample size (1692 women), by random sampling | Training given to all data collectors. Multiple data collection methods: open-ended questionnaire and in-depth interviews | Little description of analysis | All author inferences supported by quotes or quantitative data. Multiple illustrative quotes, lack of identification of separate participants | Several sections explicitly referred to washing, reuse and/or disposal of menstrual materials | Balance of open and closed ended responses. Consent sated for interviews, no information on consent for questionaries. Unnamed quotes for confidentiality. |
|  | Trustworthiness: Medium | | | | Relevance: Medium | |
| Visaria and Mishra, 2017 [77] | Large sample size (585 adolescent girls) by random stratified sampling  *(total number of participants not explicitly stated)* | Multiple data collection methods: MHM Training Program, followed by a semi-structured questionnaire, interviews, and focus group discussions to assess relative knowledge. Questions were pre-tested and canvassed before use. | Little description of analysis | Some of author inferences supported by quotes or quantitative data. few quotes, moderate identification as separate participants | Only a small proportion of the study refers to washing, reuse and/or disposal of menstrual materials | Balance of open and closed ended responses. Consent stated by participants. Anonymised quotes for confidentiality. |
|  | Trustworthiness: Medium | | | | Relevance: Medium | |
| Wardell and Czerwinski, 2001 [78] | Medium sample size (33 women), by convenience sampling | Data collected through in-depth interviews | The main researcher performed a content analysis, and then a co-researcher reviewed and validated transcripts to concur with developed themes. Several descriptive statistics are also listed (and detailed). | Most author inferences supported by quotes. Multiple illustrative quotes, lack of identification of separate participants | Several sections explicitly referred to washing, reuse and/or disposal of menstrual materials | Illustrative quotes from menstruators throughout. Written consent given by all participants. Several steps to ensure confidentiality. |
|  | Trustworthiness: Medium | | | | Relevance: High | |
| WaterAid Nepal, 2009 [79] | Large sample size (204 students), across 4 schools, by purposive sampling | Multiple data collection methods: self-administered structured close-ended questionnaire survey, focus group discussions and in-depth interviews. All three tools were pre-tested and revised for study population | Descriptive statistics were generated from quantitative survey data. Qualitative data was analysed manually based on recurrent themes and patterns. | All author inferences supported by quotes or quantitative data. Multiple illustrative quotes, moderate identification as separate participants | Several sections explicitly referred to washing, reuse and/or disposal of menstrual materials | Balance of open and closed ended responses. Verbal consent given by school principals. Little information given on consent of participants or confidentiality. |
|  | Trustworthiness: High | | | | Relevance: Medium | |
| Wilbur et al, 2021 [80] | Medium sample size (20 women and girls, and 13 carers) by purposive sampling. | Week-long training course given to all whole research team. Multiple data collection methods: in-depth interviews, photovoice and observation of accessibility issues. | Transcripts were thematically analysed by the lead author after research team had discussed all transcripts together. | All author inferences supported by quotes. Multiple illustrative quotes, moderate identification as separate participants | Only a small proportion of the study refers to washing, reuse and/or disposal of menstrual materials | Illustrative quotes from menstruators throughout. Consent given by participants where possible, and by carers if not possible by intended participant. Anonymised quotes for confidentiality. |
|  | Trustworthiness: High | | | | Relevance: Medium | |
| Wilson et al, 2014 [81] | Large sample size (302 students), across 10 schools, by purposive sampling after a cluster randomised control pilot study | Multiple data collection methods: questionnaire, training session on reusable materials, follow-up questionnaire | Covariate adjusted t-tests assessed the projected hypothesis. Sensitivity analysis was performed to assess contamination between data clusters. | Most author inferences supported by quantitative data. . No direct quotes. | Only a small proportion of the study refers to washing, reuse and/or disposal of menstrual materials | Solely quantitative data shown. No information given on consent or confidentiality. |
|  | Trustworthiness: Medium | | | | Relevance: Low | |
| Yeasmin et al, 2017 [82] | Medium sample size (43 Women, 25 Men, 14 children, 5 Feacal Sludge Emptying Operators, 4 Waste Bin Emptiers), by purposive sampling | Multiple data collection methods: in-depth interviews, focus group discussions, Pilot-tested intervention package, in-depth interviews, and focus groups | Both deductive and inductive coding was used to thematically analyse data. Transcripts were coded and categorised individually, but the team drew inferences from findings collectively. | All author inferences supported by quotes. Multiple illustrative quotes, all identified as separate participants. | A large proportion of the study refers to washing, reuse and/or disposal of menstrual materials | Balance of open and closed ended responses. Written informed consent and verbal permission was gained from all participants. Anonymised quotes for confidentiality. |
|  | Trustworthiness: High | | | | Relevance: High | |

| Number of Final Studies: | Trustworthiness | High |  | Relevance | High |  |
| --- | --- | --- | --- | --- | --- | --- |
|  |  | Medium |  |  | Medium |  |
|  |  | Low |  |  | Low |  |

*Unnamed quotes = Quotes are detailed without any identifying information (e.g. “one participant said…”)

** Anonymised Quotes = a pseudonym or alias is given for quote (e.g. FGD2 school girl / 12 year old adolescent / community worker…)

References:

1. Abera Y. Menarche, Menstruation related Problems and Practices among Adolescent High School Girls in Addis Ababa, 2003/04: Addis Ababa University; 2004.

2. Ahmmed F, Chowdhury MS, Helal SM. Sexual and reproductive health experiences of adolescent girls and women in marginalised communities in Bangladesh.Cult Health Sex 2021:1-16. doi: h<ttps://dx.doi.org/10.1080/13691058.2021.1909749.>

3. Alda-Vidal C, Browne AL. Absorbents, practices, and infrastructures: Changing socio-material landscapes of menstrual waste in Lilongwe, Malawi.Soc Cult Geogr 2021:1-21. doi: 10.1080/14649365.2021.1901974.

4. Alexander KT, Oduor C, Nyothach E, Laserson KF, Amek N, Eleveld A, et al. Water, sanitation and hygiene conditions in kenyan rural schools: Are schools meeting the needs of menstruating girls?Water, sanitation and hygiene conditions in kenyan rural schools: Are schools meeting the needs of menstruating girls? 2014;6(5):1453-66. doi: 10.3390/w6051453.

5. Asimah SA, Diabene PY, Wellington SNL. Menstrual hygiene management in Ghana: understanding the socio-cultural, economic, political factors, challenges and opportunities. Local action with international cooperation to improve and sustain water, sanitation and hygiene (WASH) services; Loughborough, UK. Loughborough: Loughborough University; 2017.

6. Averbach S, Sahin-Hodoglugil N, Musara P, Chipato T, van der Straten A. Duet® for menstrual protection: a feasibility study in Zimbabwe.Contraception 2009;79(6):463-8. doi: 10.1016/j.contraception.2008.12.002.

7. Behera D, Sivakami M, Behera MR. Menarche and Menstruation in Rural Adolescent Girls in Maharashtra, India: A Qualitative Study.J Health Manag 2015;17(4):510-9. doi: 10.1177/0972063415612581.

8. Bhattacharjee M. Menstrual Hygiene Management During Emergencies: A Study of Challenges Faced by Women and Adolescent Girls Living in Flood-prone Districts in Assam.Indian J Gend Stud 2019;26(1-2):96-107. doi: 10.1177/0971521518811172.

9. Caruso BA, Clasen TF, Hadley C, Yount KM, Haardörfer R, Rout M, et al. Understanding and defining sanitation insecurity: Women's gendered experiences of urination, defecation and menstruation in rural Odisha, India.BMJ Glob Health 2017;2(4). doi: 10.1136/bmjgh-2017-000414.

10. Caruso BA, Dreibelbis R, Ogutu EA, Rheingans R. If you build it will they come? Factors influencing rural primary pupils' urination and defecation practices at school in western Kenya.J Water Sanit Hyg Dev 2014;4(4):642-53. doi: 10.2166/washdev.2014.028.

11. Chakravarthy V, Rajagopal S, Joshi B. Does Menstrual Hygiene Management in Urban Slums Need a Different Lens? Challenges Faced by Women and Girls in Jaipur and Delhi.Indian J Gend Stud 2019;26(1-2):138-59. doi: 10.1177/0971521518811174.

12. Chinyama J, Chipungu J, Rudd C, Mwale M, Verstraete L, Sikamo C, et al. Menstrual hygiene management in rural schools of Zambia: A descriptive study of knowledge, experiences and challenges faced by schoolgirls.BMC Public Health 2019;19(1). doi: 10.1186/s12889-018-6360-2.

13. Chothe V, Khubchandani J, Seabert D, Asalkar M, Rakshe S, Firke A, et al. Students' Perceptions and Doubts About Menstruation in Developing Countries: A Case Study From India.Health Educ Res 2014;15(3):319-26. doi: 10.1177/1524839914525175.

14. Connolly S, Sommer M. Cambodian girls' recommendations for facilitating menstrual hygiene management in school.J Water Sanit Hyg Dev 2013;3(4):612-22. doi: 10.2166/washdev.2013.168.

15. Coswosk ÉD, Neves-Silva P, Modena CM, Heller L. Having a toilet is not enough: The limitations in fulfilling the human rights to water and sanitation in a municipal school in Bahia, Brazil.BMC Public Health 2019;19(1). doi: 10.1186/s12889-019-6469-y.

16. Crankshaw TL, Strauss M, Gumede B. Menstrual health management and schooling experience amongst female learners in Gauteng, South Africa: a mixed method study.Reprod Health 2020;17(1):1-15. doi: 10.1186/s12978-020-0896-1. PubMed PMID: 142737882. Language: English. Entry Date: 20200420. Revision Date: 20200429. Publication Type: Article.

17. Crichton J, Okal J, Kabiru CW, Zulu EM. Emotional and Psychosocial Aspects of Menstrual Poverty in Resource-Poor Settings: A Qualitative Study of the Experiences of Adolescent Girls in an Informal Settlement in Nairobi.Health Care Women Int 2013;34(10):891-916. doi: 10.1080/07399332.2012.740112.

18. Crofts T, J., Fisher J, editors. Schoolgirls' experiences of managing menstrual hygiene in Uganda. 2011 35th WEDC International Conference - The Future of Water, Sanitation and Hygiene in Low-Income Countries: Innovation, Adaptation and Engagement in a Changing World; 2011; Loughborough.

19. Crofts T, Fisher J. Menstrual hygiene in Ugandan schools: An investigation of low-cost sanitary pads.J Water Sanit Hyg Dev 2012;2(1):50-8. doi: 10.2166/washdev.2012.067.

20. Daniels GJ. Investigating Fear, Shyness, And Discomfort Related To Menstrual Hygiene Management In Rural Cambodia: Yale University; 2016.

21. Dhingra R, Kumar A, Kour M. Knowledge and practices related to menstruation among Tribal (Gujjar) adolescent girls.Studies on Ethno-Medicine 2009;3(1):43-8. doi: 10.1080/09735070.2009.11886336.

22. Dolan CS, Ryus CR, Dopson S, Montgomery P, Scott L. A blind spot in girls' education: Menarche and its webs of exclusion in Ghana.J Int Dev 2014;26(5):643-57. doi: 10.1002/jid.2917.

23. Enoch A, Nadutey A, Afful BF, Anokye R. Menstrual Hygiene Management: Challenges and Coping Strategies for Adolescents with Disabilities in the Kumasi Metro of Ghana.Disability, CBR & Inclusive Development 2020;31(2):77-91. doi: 10.47985/dcidj.364. PubMed PMID: 147075516. Language: English. Entry Date: 20201126. Revision Date: 20201126. Publication Type: Article.

24. Ellis A, Haver J, Villasenor J, Parawan A, Venkatesh M, Freeman MC, et al. WASH challenges to girls' menstrual hygiene management in Metro Manila, Masbate, and South Central Mindanao, Philippines.Waterlines 2016;35(3):306-23. doi: 10.3362/1756-3488.2016.022.

25. Garikipati S, Boudot C. To Pad or Not to Pad: Towards Better Sanitary Care for Women in Indian Slums.J Int Dev 2017;29(1):32-51. doi: 10.1002/jid.3266.

26. George AM, Leena KC. Experiences of the Women Using Menstrual Cup on Free Will - A Qualitative Inquiry.Online J Health Allied Sci 2020;19(3):1-4. PubMed PMID: 148350824. Language: English. Entry Date: 20210202. Revision Date: 20210203. Publication Type: Article.

27. Girod C, Ellis A, Andes KL, Freeman MC, Caruso BA. Physical, Social, and Political Inequities Constraining Girls’ Menstrual Management at Schools in Informal Settlements of Nairobi, Kenya.J Urban Health 2017;94(6):835-46. doi: 10.1007/s11524-017-0189-3.

28. Gultie T, Hailu D, Workineh Y. Age of menarche and knowledge about menstrual hygiene management among adolescent school girls in amhara province, Ethiopia: Implication to health care workers & school teachers.PLoS One 2014;9(9). doi: 10.1371/journal.pone.0108644.

29. Habtegiorgis Y, Sisay T, Kloos H, Malede A, Yalew M, Arefaynie M, et al. Menstrual hygiene practices among high school girls in urban areas in Northeastern Ethiopia: A neglected issue in water, sanitation, and hygiene research.PLoS One 2021;16(6):e0248825. doi: h<ttps://dx.doi.org/10.1371/journal.pone.0248825.>

30. Hawkins A, Sharpe R, Spence K, Holmes N. Inappropriate flushing of menstrual sanitary products.Proceedings of the Institution of Civil Engineers-Water Management 2019;172(4):163-9. doi: 10.1680/jwama.17.00050. PubMed PMID: WOS:000475712900001.

31. Hennegan J, Kibira SPS, Exum NG, Schwab KJ, Makumbi FE, Bukenya J. 'I do what a woman should do': a grounded theory study of women's menstrual experiences at work in Mukono District, Uganda.BMJ Glob Health 2020;5(11). doi: h<ttps://dx.doi.org/10.1136/bmjgh-2020-003433.>

32. Hennegan J, Sol L. Confidence to manage menstruation at home and at school: findings from a cross-sectional survey of schoolgirls in rural Bangladesh.Cult Health Sex 2020;22(2):146-65. doi: h<ttps://dx.doi.org/10.1080/13691058.2019.1580768.>

33. Hennegan J, Dolan C, Steinfield L, Montgomery P. A qualitative understanding of the effects of reusable sanitary pads and puberty education: Implications for future research and practice.Reprod Health 2017;14(1). doi: 10.1186/s12978-017-0339-9.

34. Hennegan J, Dolan C, Wu M, Scott L, Montgomery P. Measuring the prevalence and impact of poor menstrual hygiene management: A quantitative survey of schoolgirls in rural Uganda.BMJ Open 2016;6(12). doi: 10.1136/bmjopen-2016-012596.

35. Htun NN, Laosee O, Rattanapan C. Factors that influence menstrual hygiene management in adolescent girls in Mudon Township, Mon State, Myanmar.J Health Sci Med Res JHSMR 2021;39(3):207-17. doi: 10.31584/jhsmr.2021778.

36. Jahan F, Nuruzzaman M, Sultana F, Mahfuz MT, Rahman M, Akhand F, et al. Piloting an acceptable and feasible menstrual hygiene products disposal system in urban and rural schools in Bangladesh.BMC Public Health 2020;20(1):N.PAG-N.PAG. doi: 10.1186/s12889-020-09413-x. PubMed PMID: 145535939. Language: English. Entry Date: In Process. Revision Date: 20210103. Publication Type: journal article. Journal Subset: Biomedical.

37. Kambala C, Chinangwa A, Chipeta E, Torondel B, Morse T. Acceptability of menstrual products interventions for menstrual hygiene management among women and girls in Malawi.Reprod Health 2020;17(1):N.PAG-N.PAG. doi: 10.1186/s12978-020-01045-z. PubMed PMID: 147156425. Language: English. Entry Date: 20201127. Revision Date: 20201127. Publication Type: Article.

38. Karibu K, Salami JC, Azeez M, Azeez A. Onset of Menarche and Adolescent Menstrual Hygiene Practices in Semi-Urban Ibadan Community, Nigeria.Womens Reprod Health 2019;6(2):102-17.

39. Kemigisha E, Rai M, Mlahagwa W, Nyakato VN, Ivanova O. A qualitative study exploring menstruation experiences and practices among adolescent girls living in the nakivale refugee settlement, Uganda.Int J Environ Res Public Health 2020;17(18):1-11. doi: 10.3390/ijerph17186613.

40. Kohler P, Renggli S, Lüthi C. WASH and gender in health care facilities: The uncharted territory.WASH and gender in health care facilities: The uncharted territory 2019;40(1):3-12. doi: 10.1080/07399332.2017.1395440.

41. Kumbeni MT, Otupiri E, Ziba FA. Menstrual hygiene among adolescent girls in junior high schools in rural northern Ghana.Pan Afr Med J 2020;37:190. doi: h<ttps://dx.doi.org/10.11604/pamj.2020.37.190.19015.>

42. Lahme AM, Stern R, Cooper D. Factors impacting on menstrual hygiene and their implications for health promotion.Glob Health Promot 2018;25(1):54-62. doi: 10.1177/1757975916648301.

43. MacRae ER, Clasen T, Dasmohapatra M, Caruso BA. 'It's like a burden on the head': Redefining adequate menstrual hygiene management throughout women's varied life stages in Odisha, India.PLoS One 2019;14(8):e0220114. doi: h<ttps://dx.doi.org/10.1371/journal.pone.0220114.>

44. Mason L, Nyothach E, Alexander K, Odhiambo FO, Eleveld A, Vulule J, et al. 'We keep it secret so no one should know' - A qualitative study to explore young schoolgirls attitudes and experiences with menstruation in rural Western Kenya.PLoS One 2013;8(11). doi: 10.1371/journal.pone.0079132.

45. Maulingin-Gumbaketi E, Larkins S, Gunnarsson R, Rembeck G, Whittaker M, Redman-MacLaren M. 'Making of a Strong Woman': a constructivist grounded theory of the experiences of young women around menarche in Papua New Guinea.BMC Womens Health 2021;21(1):144. doi: h<ttps://dx.doi.org/10.1186/s12905-021-01229-0.>

46. McHenga J, Phuma-Ngaiyaye E, Kasulo V. Do sanitation facilities in primary and secondary schools address Menstrual Hygiene needs? A study from Mzuzu City, Malawi.Phys Chem Earth 2020;115. doi: 10.1016/j.pce.2020.102842.

47. Miiro G, Rutakumwa R, Nakiyingi-Miiro J, Nakuya K, Musoke S, Namakula J, et al. Menstrual health and school absenteeism among adolescent girls in Uganda (MENISCUS): A feasibility study.BMC Womens Health 2018;18(1). doi: 10.1186/s12905-017-0502-z.

48. Mohamed Y, Durrant K, Huggett C, Davis J, Macintyre A, Menu S, et al. A qualitative exploration of menstruation-related restrictive practices in Fiji, Solomon Islands and Papua New Guinea.PLoS One 2018;13(12). doi: 10.1371/journal.pone.0208224.

49. Mohammed S, Larsen-Reindorf RE, Awal I. Menstrual Hygiene Management and School Absenteeism among Adolescents in Ghana: Results from a School-Based Cross-Sectional Study in a Rural Community.Int J Reprod Med 2020;2020:6872491. doi: h<ttps://dx.doi.org/10.1155/2020/6872491.>

50. Mohammed S, Emil Larsen-Reindorf R. Menstrual knowledge, sociocultural restrictions, and barriers to menstrual hygiene management in Ghana: Evidence from a multi-method survey among adolescent schoolgirls and schoolboys.PLoS One 2020;15(10 October). doi: 10.1371/journal.pone.0241106.

51. Mumtaz Z, Sivananthajothy P, Bhatti A, Sommer M. "How can we leave the traditions of our Baab Daada" socio-cultural structures and values driving menstrual hygiene management challenges in schools in Pakistan.J Adolesc 2019;76:152-61. doi: 10.1016/j.adolescence.2019.07.008. PubMed PMID: 139192218.

52. Muralidharan A. Constrained Choices? Menstrual Health and Hygiene Needs Among Adolescents in Mumbai Slums.Indian J Gend Stud 2019;26(1-2):12-39. doi: 10.1177/0971521518808104.

53. Nalugya R, Tanton C, Hytti L, Kansiime C, Nakuya K, Namirembe P, et al. Assessing the effectiveness of a comprehensive menstrual health intervention program in Ugandan schools (MENISCUS): process evaluation of a pilot intervention study.Pilot Feasibility Stud 2020;6:51. doi: h<ttps://dx.doi.org/10.1186/s40814-020-00585-2.>

54. Ndlovu E, Bhala E. Menstrual hygiene - A salient hazard in rural schools: A case of Masvingo district of Zimbabwe.Jamba 2016;8(2):1-8. doi: 10.4102/jamba.v8i2.204.

55. Oche M, O., Umar A, S., Gana G, J., Ango J, T. Menstrual health: the unmet needs of adolescent girls’ in Sokoto, Nigeria.Scientific Research and Essays 2012;7(3):410-18.

56. Parker AH, Smith JA, Verdemato T, Cooke J, Webster J, Carter RC. Menstrual management: A neglected aspect of hygiene interventions.Disaster Prev Manag 2014;23(4):437-54. doi: 10.1108/DPM-04-2013-0070.

57. Rajagopal S, Mathur K. ‘Breaking the silence around menstruation’: experiences of adolescent girls in an urban setting in India.Gend Dev 2017;25(2):303-17. doi: 10.1080/13552074.2017.1335451.

58. Rajaraman D, Travasso SM, Heymann SJ. A qualitative study of access to sanitation amongst low-income working women in Bangalore, India.J Water Sanit Hyg Dev 2013;3(3):432-40. doi: 10.2166/washdev.2013.114.

59. Ramathuba DU. Menstrual knowledge and practices of female adolescents in Vhembe district, Limpopo Province, South Africa.Curationis 2015;38(1). doi: 10.4102/curationis.v38i1.1551.

60. Rastogi S, Khanna A, Mathur P. Uncovering the challenges to menstrual health: Knowledge, attitudes and practices of adolescent girls in government schools of Delhi.Health Educ J 2019;78(7):839-50. doi: 10.1177/0017896919850209.

61. Rheinländer T, Gyapong M, Akpakli DE, Konradsen F. Secrets, shame and discipline: School girls' experiences of sanitation and menstrual hygiene management in a peri-urban community in Ghana.Health Care Women Int 2019;40(1):13-32. doi: 10.1080/07399332.2018.1444041.

62. Rizvi N, Ali TS. Misconceptions and Mismanagement of Menstruation among Adolescents Girls who do not attend School in Pakistan.Journal of Asian Midwives 2016;3(1):46–62.

63. Roxburgh H, Hampshire K, Kaliwo T, Tilley EA, Tilley EA, Oliver DM, et al. Power, danger, and secrecy-A socio-cultural examination of menstrual waste management in urban Malawi.PLoS One 2020;15(6 June). doi: 10.1371/journal.pone.0235339.

64. Schmitt ML, Wood OR, Clatworthy D, Rashid SF, Sommer M. Innovative strategies for providing menstruation-supportive water, sanitation and hygiene (WASH) facilities: learning from refugee camps in Cox's bazar, Bangladesh.Confl Health 2021;15(1):10. doi: <https://dx.doi.org/10.1186/s13031-021-00346-9>.

65. Schmitt ML, Clatworthy D, Ratnayake R, Klaesener-Metzner N, Roesch E, Wheeler E, et al. Understanding the menstrual hygiene management challenges facing displaced girls and women: Findings from qualitative assessments in Myanmar and Lebanon.Confl Health 2017;11(1). doi: 10.1186/s13031-017-0121-1.

66. Scorgie F, Foster J, Stadler J, Phiri T, Hoppenjans L, Rees H, et al. “Bitten By Shyness”: Menstrual Hygiene Management, Sanitation, and the Quest for Privacy in South Africa.“Bitten By Shyness”: Menstrual Hygiene Management, Sanitation, and the Quest for Privacy in South Africa 2016;35(2):161-76. doi: 10.1080/01459740.2015.1094067.

67. Shah V, Nabwera HM, Sosseh F, Jallow Y, Comma E, Keita O, et al. A rite of passage: A mixed methodology study about knowledge, perceptions and practices of menstrual hygiene management in rural Gambia.BMC Public Health 2019;19(1). doi: 10.1186/s12889-019-6599-2.

68. Sheoran P, Kaur S, Lata H, Sarin J. A descriptive study of menstrual hygiene practices among women at the rural area of Haryana.Journal of Nursing & Midwifery Sciences 2020;7(4):269-73. doi: 10.4103/JNMS.JNMS_18_20. PubMed PMID: 146546537. Language: English. Entry Date: 20201103. Revision Date: 20201103. Publication Type: Article.

69. Sivakami M, van Eijk AM, Thakur H, Kakade N, Patil C, Shinde S, et al. Effect of menstruation on girls and their schooling, and facilitators of menstrual hygiene management in schools: Surveys in government schools in three states in India, 2015.J Glob Health 2019;9(1). doi: 10.7189/jogh.09.010408.

70. Sommer M, Gruer C, Smith RC, Maroko A, Hopper K. Menstruation and homelessness: Challenges faced living in shelters and on the street in New York City.Health Place 2020;66. doi: 10.1016/j.healthplace.2020.102431. PubMed PMID: WOS:000594147200014.

71. Sommer M, Ackatia-Armah N, Connolly S, Smiles D. A comparison of the menstruation and education experiences of girls in Tanzania, Ghana, Cambodia and Ethiopia.Compare 2015;45(4):589-609. doi: 10.1080/03057925.2013.871399.

72. Sommer M. Ideologies of sexuality, menstruation and risk: girls' experiences of puberty and schooling in northern Tanzania.Cult Health Sex 2009;11(4):383-98. doi: 10.1080/13691050902722372.

73. Tamiru S, Mamo K, Acidria P, Mushi R, Ali CS, Ndebele L. Towards a sustainable solution for school menstrual hygiene management: Cases of Ethiopia, Uganda, South-Sudan, Tanzania, and Zimbabwe.Waterlines 2015;34(1):92-102. doi: 10.3362/1756-3488.2015.009.

74. Tegegne TK, Sisay MM. Menstrual hygiene management and school absenteeism among female adolescent students in Northeast Ethiopia.BMC Public Health 2014;14(1). doi: 10.1186/1471-2458-14-1118.

75. Trinies V, Caruso BA, Sogoré A, Toubkiss J, Freeman MC. Uncovering the challenges to menstrual hygiene management in schools in Mali.Waterlines 2015;34(1):31-40. doi: 10.3362/1756-3488.2015.004.

76. Umeora OU, Egwuatu VE. Menstruation in rural Igbo women of south east Nigeria: attitudes, beliefs and practices.Afr J Reprod Health 2008;12(1):109-15. doi: 10.2307/25470641.

77. Visaria L, Mishra RN. Health Training Programme for Adolescent Girls: Some Lessons from India’s NGO Initiative.J Health Manag 2017;19(1):97-108. doi: 10.1177/0972063416682586.

78. Wardell DW, Czerwinski B. A military challenge to managing feminine and personal hygiene.J Am Acad Nurse Pract 2001;13(4):187-93.

79. WaterAid Nepal. Is Menstrual Hygiene and Management an Issue for Adolescent School Girls? A Comparative Study of Four Schools in Different Settings of Nepal. Kathmandu: WaterAid Nepal, 2009.

80. Wilbur J, Kayastha S, Mahon T, Torondel B, Hameed S, Sigdel A, et al. Qualitative study exploring the barriers to menstrual hygiene management faced by adolescents and young people with a disability, and their carers in the Kavrepalanchok district, Nepal.BMC Public Health 2021;21(1):1-15. doi: 10.1186/s12889-021-10439-y. PubMed PMID: 149171386. Language: English. Entry Date: In Process. Revision Date: 20210315. Publication Type: Article. Journal Subset: Biomedical.

81. Wilson E, Reeve J, Pitt A. Education. Period. Developing an acceptable and replicable menstrual hygiene intervention.Dev Pract 2014;24(1):63-80. doi: 10.1080/09614524.2014.867305.

82. Yeasmin F, Luby SP, Saxton RE, Nizame FA, Alam MU, Dutta NC, et al. Piloting a low-cost hardware intervention to reduce improper disposal of solid waste in communal toilets in low-income settlements in Dhaka, Bangladesh.BMC Public Health 2017;17(1). doi: 10.1186/s12889-017-4693-x.
