# Supplementary material for: Drivers of menstrual material disposal and washing practices: A systematic review
Source: PLoS One. 2021 Dec 3;16(12):e0260472. doi: 10.1371/journal.pone.0260472 (PMC8641861; doi:10.1371/journal.pone.0260472)
Supplement: S3 Table — (DOCX) [file pone.0260472.s004.docx]

S3 Table: Illustrative examples showing drivers of behaviours

| **Driver** | **Explanation** | **Examples** |
| --- | --- | --- |
| State of available facilities | Physical Infrastructure  *(Does the sanitation facility meet desired physical sanitation needs?)* | - Quantity of available working toilets/latrines   - Number of useable vs. dilapidated facilities (e.g., broken doors)   - Number of facilities desired by the user*   - Accessibility of toilets / latrines accessible when needed (e.g., facilities locked/unlocked) - Design of toilets/latrines   - Presence/absence of a lid   - Amount of space for washing and/or drying menstrual materials   - Presence/absence of toilet/latrine door   - Amount of space to dry reusable products - Quality / availability of running water in / around toilet / latrines - Availability of soap for washing - Availability of a disposal mechanism and/or service for used material (e.g. bins, incinerators, waste collection service)   **Self-defined by menstruators specifying there were too few facilities for the given sample population* |
|  | Social Perceptions  *(Does the sanitation facility meet desired social needs?)* | - Presence/absence of a private/safe space for managing menstruation (e.g., gaps in toilet stalls which people can peer through, and locks on individual cubicle doors) - Cleanliness and maintenance of the facility - Time available to change / wash / dispose of materials when using facilities - Availability of gender-segregated toilets / latrines |
| Knowledge | Lack of knowledge  *(Menstruators* ***have not*** ***been taught*** *how to dispose / wash / dry materials)* | - Explicit instances of menstruators explaining a lack of awareness of how to dispose, wash and or dry materials, e.g.,   - Male educators did not teach menstrual health practises   - Family members did not provide information to new menstruators due to stigmatisation and beliefs that it is a ‘private matter’   - Menstruators sought out information, but only from those who were also unaware of safe/appropriate practises (e.g. peers)   - General feelings of discomfort when discussing menstrual needs and practises, leading to less information being sought/relayed |
| Menstrual Taboos and Social Stigma | Cultural Beliefs  *(****General beliefs*** *discouraging / encouraging certain methods of disposal)* | - Cultural beliefs, e.g.,   - Hiding materials whilst drying so as not to let males see   - Wrapping used materials before disposal so as not to be perceived as careless, or cause ‘black visual disease’ to men who see openly disposed materials   - Avoiding burning used materials so as not to be destroying ‘part of the womb’ (*lest it cause cancer or infertility)*   - Washing used materials prior to disposal so that the blood cannot be used by others   - Hiding used materials to avoid witches / satanists / metaphysical forces / black magic using menstrual blood for harmful rituals to invoke in others:     - Stomach pains     - Infertility     - Blindness     - Fever     - Cancer |
|  | Embarrassment and Worry  (unpleasant emotions related to doing something considered by others to be wrong or shameful) | - Worrying about the possibility of, or embarrassment/shame as a result of, being seen by others when changing, disposing of, washing and/or drying menstrual materials, e.g.,   - Entering/leaving toilets/latrines   - Disposing/burying menstrual materials   - Carrying materials to/from toilets/latrines   - Others seeing used materials in toilets/latrines   - Staining of clothing |
|  | Fear  (unpleasant emotion caused by the threat of danger, pain or other harmful consequences) | - Fear of blood being seen by others when changing, disposing of, washing and/or drying menstrual materials, e.g.,   - By boys/other students/teachers at school (possibly leading to sexual advances)   - By fathers in the home - Fear of not having appropriate facilities/menstrual materials to ensure concealment of menstruation - Fear of becoming an adult/being able to get married - Fear of physical abuse from ‘sinning’ by someone seeing used material / blood - Fear of gender-based violence in sanitation facilities - Fear of becoming infertile due to a curse linked to an inadequate method of menstrual material disposal |
