# Supplementary material for: Drivers of menstrual material disposal and washing practices: A systematic review
Source: PLoS One. 2021 Dec 3;16(12):e0260472. doi: 10.1371/journal.pone.0260472 (PMC8641861; doi:10.1371/journal.pone.0260472)
Supplement: Attachment — Submitted filename: PONE-D-21-01105_reviewer corr-11.3.2021.docx [file pone.0260472.s005.docx]

PLOS ONE

Drivers of menstrual material disposal and washing practices: A systematic review

--Manuscript Draft--

| Manuscript Number: | PONE-D-21-01105 |
| --- | --- |
| Article Type: | Research Article |
| Full Title: | Drivers of menstrual material disposal and washing practices: A systematic review |
| Short Title: | Drivers of menstrual material disposal and washing practices: A systematic review |
| Corresponding Author: | Hannah Jayne Robinson, BEng  University of Leeds  Leeds, West Yorkshire UNITED KINGDOM |
| Keywords: | Menstruation; systematic review; Disposal; Washing; Behavioural drivers; Sanitation Facilities; Taboo |
| Abstract: | Background: Disposal and washing facilities and services for menstrual materials are often designed based upon technical specifications rather than an in-depth understanding of what drives peoples’ choices of practices.  Objectives and data sources: This systematic review identified and summarised the main behavioural drivers pertaining to the choice of disposal and washing methods of menstrual materials through the thematic content analysis and study appraisal of 55 publications (54 studies) on menstrual health and hygiene published since 1999, reporting the outcomes of primary research across 25 countries.  Results: Disposal and washing behaviours are primarily driven by the physical state of sanitation facilities; however, this is intrinsically linked to taboos surrounding and knowledge of menstruation.  Implications: Using reasons given for disposal and washing practices by menstruators or those who know them well, or inferred by authors, we identify the key considerations needed to design facilities and services which best suit the desired behaviours of both planners and those who menstruate.  Registration: The review protocol is registered on PROSPERO: 42019140029 Inclusivity: The term menstruators is used throughout to encompass all those mentioned in the studies reviewed (girls and women); although no studies explicitly stated including non-binary or transgender participants, this review uses inclusive language that represents the spectrum of genders that may experience menstruation. |
| Order of Authors: | Hannah Jayne Robinson, BEng |
|  | Dani Jennifer Barrington |
| Additional Information: |  |
| Question | Response |
| Financial Disclosure  Enter a financial disclosure statement that describes the sources of funding for the work included in this submission. Review the [submission guidelines](http://journals.plos.org/plosone/s/submission-guidelines#loc-financial-disclosure-statement) for detailed requirements. View published research articles from [PLOS ONE](http://journals.plos.org/plosone/) for specific examples.  This statement is required for submission and will appear in the published article if the submission is accepted. Please make sure it is accurate. | The lead author of this paper is supported by the Engineering and Physical Sciences Research Council Grant number EP/S022066/1 |

| Unfunded studies Enter: The author(s) received no specific funding for this work. Funded studies Enter a statement with the following details: - Initials of the authors who received each award - Grant numbers awarded to each author - The full name of each funder - URL of each funder website - Did the sponsors or funders play any role in the study design, data collection and analysis, decision to publish, or preparation of the manuscript? - NO - Include this sentence at the end of your statement: The funders had no role in study design, data collection and analysis, decision to publish, or preparation of the manuscript. - YES - Specify the role(s) played. |  |
| --- | --- |
| * typeset |  |
| Competing Interests  Use the instructions below to enter a competing interest statement for this submission. On behalf of all authors, disclose any [competing interests](http://journals.plos.org/plosone/s/competing-interests) that could be perceived to bias this work—acknowledging all financial support and any other relevant financial or nonfinancial competing interests.  This statement will appear in the published article if the submission is accepted. Please make sure it is accurate. View published research articles from [PLOS ONE](http://journals.plos.org/plosone/) for specific examples. | The authors have declared that no competing interests exist. |

| NO authors have competing interests Enter: The authors have declared that no competing interests exist. Authors with competing interests Enter competing interest details beginning with this statement: I have read the journal's policy and the authors of this manuscript have the following competing interests: [insert competing interests here] |  |
| --- | --- |
| * typeset |  |
| Ethics Statement  Enter an ethics statement for this submission. This statement is required if the study involved:   - Human participants - Human specimens or tissue - Vertebrate animals or cephalopods - Vertebrate embryos or tissues - Field research   Write "N/A" if the submission does not require an ethics statement.  General guidance is provided below. Consult the [submission guidelines](http://journals.plos.org/plosone/s/submission-guidelines#loc-guidelines-for-specific-study-types) for detailed instructions. Make sure that all information entered here is included in the Methods section of the manuscript. | N/A |

Format for specific study types

Human Subject Research (involving human

participants and/or tissue)

Give the name of the institutional review

board or ethics committee that approved the

study

•

Include the approval number and/or a

statement indicating approval of this

research

•

Indicate the form of consent obtained

(

written/oral) or the reason that consent was

not obtained (e.g. the data were analyzed

anonymously)

•

Animal Research (involving vertebrate

animals, embryos or tissues)

Provide the name of the Institutional Animal

Care and Use Committee (IACUC) or other

relevant ethics board that reviewed the

study protocol, and indicate whether they

approved this research or granted a formal

waiver of ethical approval

•

Include an approval number if one was

obtained

•

If the study involved

non-human primates

,

add

additional details

about animal welfare

and steps taken to ameliorate suffering

•

If anesthesia, euthanasia, or any kind of

animal sacrifice is part of the study, include

briefly which substances and/or methods

were applied

•

Field Research

Include the following details if this study

involves the collection of plant, animal, or

other materials from a natural setting:

Field permit number

•

Name of the institution or relevant body that

granted permission

•

Data Availability

Authors are required to make all data

underlying the findings described fully

available, without restriction, and from the

time of publication. PLOS allows rare

exceptions to address legal and ethical

concerns. See the

[PLOS Data Polic](http://journals.plos.org/plosone/s/data-availability)

[y](http://journals.plos.org/plosone/s/data-availability)

and

[FA](http://journals.plos.org/plosone/s/data-availability#loc-faqs-for-data-policy)

[Q](http://journals.plos.org/plosone/s/data-availability#loc-faqs-for-data-policy)

for detailed information.

Yes - all data are fully available without restriction

| A Data Availability Statement describing where the data can be found is required at submission. Your answers to this question constitute the Data Availability Statement and will be published in the article, if accepted. |  |
| --- | --- |
| Important: Stating ‘data available on request from the author’ is not sufficient. If your data are only available upon request, select ‘No’ for the first question and explain your exceptional situation in the text box. |  |
| Do the authors confirm that all data underlying the findings described in their manuscript are fully available without restriction? |  |
| Describe where the data may be found in full sentences. If you are copying our sample text, replace any instances of XXX with the appropriate details.   - If the data are held or will be held in a public repository, include URLs, accession numbers or DOIs. If this information will only be available after acceptance, indicate this by ticking the box below. For example: All XXX files   are available from the XXX database (accession number(s) XXX, XXX.).   - If the data are all contained within the manuscript and/or Supporting Information files, enter the following: All relevant data are within the manuscript and its Supporting Information files. - If neither of these applies but you are able to provide details of access elsewhere, with or without limitations, please do so. For example:   Data cannot be shared publicly because of [XXX]. Data are available from the XXX Institutional Data Access / Ethics Committee (contact via XXX) for researchers who meet the criteria for access to confidential data.  The data underlying the results presented in the study are available from (include the name of the third party | No primary data, all reviewed documents available online |

| and contact information or URL).  • This text is appropriate if the data are owned by a third party and authors do not have permission to share the data.  * typeset |  |
| --- | --- |
| Additional data availability information: |  |

[Click here to access/download;Manuscript;2021.01.22 Disposal Paper - HJR DJB for submission.docx](https://www.editorialmanager.com/pone/download.aspx?id=28251286&guid=2e45f15e-6080-404a-8f0b-34adc3a44c75&scheme=1)

# Drivers of menstrual material disposal and washing practices: A systematic review

**Hannah Jayne Robinson^1*^, Dani Jennifer Barrington^1,2^**

1. University of Leeds, Woodhouse Lane, Leeds LS2 9JT, West Yorkshire, United Kingdom
2. The University of Western Australia, 35 Stirling Highway, Crawley 6008, Western Australia, Australia

*Corresponding author, cn16hjr@leeds.ac.uk

# Abstract

**Background:** Disposal and washing facilities and services for menstrual materials are often designed based upon technical specifications rather than an in-depth understanding of what drives peoples’ choices of practices.

**Objectives and data sources:** This systematic review identified and summarised the main behavioural drivers pertaining to the choice of disposal and washing methods of menstrual materials through the thematic content analysis and study appraisal of 55 publications (54 studies) on menstrual health and hygiene published since 1999, reporting the outcomes of primary research across 25 countries.

**Results:** Disposal and washing behaviours are primarily driven by the physical state of sanitation facilities; however, this is intrinsically linked to taboos surrounding and knowledge of menstruation.

**Implications:** Using reasons given for disposal and washing practices by menstruators or those who know them well, or inferred by authors, we identify the key considerations needed to design facilities and services which best suit the desired behaviours of both planners and those who menstruate.

**Registration:** The review protocol is registered on PROSPERO: [42019140029](https://www.crd.york.ac.uk/prospero/display_record.php?ID=CRD42019140029)

**Inclusivity:** The term menstruators is used throughout to encompass all those mentioned in the studies reviewed (girls and women); although no studies explicitly stated including non-binary or transgender participants, this review uses inclusive language that represents the spectrum of genders that may experience menstruation.

***Keywords:*** *Menstruation; Systematic Review; Disposal; Washing; Behavioural drivers; Sanitation*

*Facilities; Taboo*

# 1. Introduction

Menstrual health and hygiene (MHH) are an integral part of public health, recognized by an increase in research on this topic in the past decade (e.g. 1, 2). Within the MHH space there has been a lot of research into the provision of menstrual materials, and subsequent interventions that provide those who menstruate with both reusable and disposable materials (3, 4, 5). However, there has been less research into what happens once these materials have been used; the full lifecycle of these materials has often not been documented.

Understanding the full lifecycle of menstrual materials is especially important for those designing the infrastructure of water, sanitation and hygiene (WASH) programs, including, but not limited to, toilets, bathing facilities, washing and drying facilities, incinerators, and solid waste management services. WASH facilities need to be technically and socially appropriate to allow for people to change and dispose of menstrual materials safely for them, the associated infrastructural systems and the environment. Disposal choices directly affect the functioning of sanitation systems; if materials are discarded in toilets or pit latrines, they can create blockages which reduce functionality of a system (6). Disposal and washing practices can also have adverse effects on users, for example, if there are no spaces for drying reusable materials, it is possible that infections could manifest if materials are used before they are dried properly

(7).

Currently, the drivers behind menstruators’ choice of disposal and washing practices are not documented and rarely considered when WASH facilities are designed. For example, although it alludes to the need for ‘cultural considerations’ when designing facilities, the International Standard on Non-Sewered

Sanitation requires technology developers to provide users with instructions on how to dispose of their menstrual materials so as to protect the functioning of the technology, but this does not necessarily consider the reasons why a user may choose to flush materials despite knowing that it may harm the infrastructure (8). This views users through a deficit lens (9), it assumes that if only they knew better they would change their behaviour. But WASH behaviours are not always driven by possessing the appropriate ‘knowledge’, for example, people may have been taught that it is unsafe to practice open defecation but choose to do so for reasons of convenience, pride and mental well-being (10). Such disconnects between ‘knowledge’ and ‘action’ are why the field of WASH behaviour change scholarship exists (11).

Two systematic reviews have been published compiling the methods of menstrual disposal used in low and middle income countries (12, 13), but neither thoroughly investigates why users practice these behaviours. A recent critical review of ‘unflushable’ objects entering waterborne sewerage highlights the lack of research into the drivers behind user decisions to dispose of non-biodegradable materials in toilets around the world (14). No reviews have been published on menstrual material washing practices.

To better design WASH systems that meet the needs of those who menstruate it is important to understand what disposal and washing practices are currently used, but more importantly, why: if WASH professionals can understand what drives washing and disposal methods in different contexts, they can design technically robust systems that those who menstruate want to use. To address this, we systematically investigated the extant peer-reviewed literature on menstrual disposal and washing practices so as to determine: 1) What drives the behaviours of those who menstruate when deciding on a method of disposal or washing of used menstrual products?; 2) Are there differences in behavioural drivers and practices between low, middle and high income economies?; 3) If menstrual health programming is to be socially, environmentally, economically and technically sustainable, how does it need to engage with the drivers of behaviour around disposal and washing?

# 2. Method

The review protocol is registered on PROSPERO: [42019140029](https://www.crd.york.ac.uk/prospero/display_record.php?ID=CRD42019140029)

(https://www.crd.york.ac.uk/prospero/display_record.php?RecordID=140029) and is reported according to PRISMA guidance (15).

## 2.1 Search Strategy

A systematic search of peer-reviewed literature was conducted according to the PRISMA guidelines (16) (Figure 1). The search was performed on 20^th^ June 2019, and included documents published in the last 20 years, to ensure that findings were relevant to current disposal and washing practices.

Topics to be searched for in the documents included: solid waste disposal; menstrual waste disposal; health effects regarding disposal and material usage, and factors affecting disposal routes. Multiple databases were searched with the following string:

*(menstru* or menarche) AND (wash* OR dispos* OR dri* OR dry* OR recycl* OR reus* OR process* OR waste* or "used product*" OR threw* OR throw* OR rubbish OR garbage OR landfill OR bin OR hide OR bury* OR buried OR burn* OR hygien*)*

This yielded the following results for initial screening: Scopus (6,787), Web of Science (4,333), EBSCO

(consisting of CINAHL, GreenFILE, MEDLINE, Social Work Abstracts, Child Development & Adolescent Studies) (5,744) and Proquest Dissertations and theses (331). We also hand-searched the bibliographies of the two existing reviews on low/middle income country menstrual material disposal methods (12, 13)

After removing duplicates, 11,888 publications were screened against Criteria 1 (primarily about menstruation or sexual and reproductive health), followed by Criteria 2 (published post-1999 and discusses behaviours post-1999; in English or has an English translation available; discusses menstrual material disposal, washing, drying and/or reuse and gives reasons for these behaviours). This led to the inclusion of 55 publications (54 studies) (Table 1 provides a summary of the included studies, for full details see Supplementary Material 1). Grey literature was not searched as the themes which arose from the peer-reviewed publications reached saturation.

Figure 1: Inclusion and Exclusion Flowchart for Systematic Review (16). Criteria 1: Primarily about menstruation or sexual and reproductive health; Criteria 2: Published post-1999 and discusses behaviours post-1999, in English or has an English translation available, discusses menstrual material disposal, washing, drying and/or reuse and gives reasons for these behaviours.

## 2.2 Quality Appraisal

Included studies were assessed on their level of trustworthiness and relevance by adapting the method of Rees et al (17) to be applicable to both quantitative and qualitative MHH studies. The assessment of trustworthiness was dependant on the sampling, data collection and analysis, and interpretation of data; this yielded high, medium, and low ratings. Relevance related to the proportion of the paper dedicated to menstrual material disposal or washing, whether confidentiality had been assured and appropriate consent obtained, and who gave the reasons for behaviours; this yielded high, medium, and low ratings (for full details of the quality appraisal of each study, see Supplementary Material 2). We considered studies which took a social constructivist approach to understand~~ing~~ drivers of menstrual disposal and washing, evidenced by the lived experiences of menstruators, to be of higher relevance than those which presented less direct evidence and tended along positivist epistemological lines. Considering the trustworthiness and relevance of studies allowed us to weight themes more heavily where they were results of robust research, particularly where the reasons for disposal and washing behaviours were given by those who menstruate themselves.

## 2.3 Thematic Content Analysis

After reviewing the full texts of each publication, deductive and inductive coding was undertaken using NVivo 12 (18):

1. Examples of practices were deductively coded as “intentional disposal” or “washing drying or reuse”.
2. Axial coding was conducted to identify the drivers behind each practice (see Table 2 for codebook and coding frequency). This led to an understanding of the influence of physical and social drivers on menstrual material disposal and washing practices and how these must be considered by those planning WASH facilities.
3. Publications were also classified demographically in order to understand the study context (Table

1 and Supplementary Material 1).

A conceptual model was developed to understand the breadth of reasoning behind disposal and washing practices.

# 3. Results

Publications detailed studies in low income economies (9 studies), lower middle income economies (42 studies), upper-middle income economies (3 studies), high income economies (1 studies), and displacement camps (2 studies) (as defined by World Bank in 2020 (19)) (Table 1 and Supplementary

Material 1). There was a skew towards girls’ (<18 years old) experiences over women’s experiences (44 instances vs. 16 instances). There were no studies which detailed the experiences of those who identify as trans-men or gender non-binary.

29 studies were rated as high trustworthiness, 24 medium trustworthiness and 1 low trustworthiness. High trustworthiness papers were characterised by having more than 50 participants, a clear analysis description, and supportive quotes that were clearly distinguishable between participants. 16 studies were considered of high relevance to this review, 32 medium relevance and 6 low relevance. High relevance studies tended to have a high proportion of the paper discussing findings relevant to disposal and washing of menstrual materials, evidence of behavioural drivers as stated by menstruators themselves, and consent and confidentiality measures stated clearly. Lower relevance studies only briefly mentioned disposal and washing behaviours and/or presented the reasons for behaviours mostly from author inferences, rather than given by menstruators themselves.

In 20 studies, reasons for disposal and washing practise were given by menstruators alone, in 14 reasons were given solely by authors, and in 20 there was a mixture of authors’ reasoning and menstruators reasoning. The reasons given for disposal and washing practices for each study are detailed in

181	Supplementary Material 1 and a summary of the frequency of reasons is detailed in Table 2. Illustrative 182	examples of the drivers of disposal and washing behaviours are provided in Supplementary Material 3.

183

184	Table 1: Included studies.

| **Citation** | **Country** | **Economic status** | **Population** | **Sample Size** |
| --- | --- | --- | --- | --- |
| Abera, 2004 (20) | Ethiopia | Low | Girls in school*, school staff | 863 questionnaires (female, grades 9-10 (≈ aged 1416), across 8 schools), 4 focus group discussions (8 students in each), and key informant interviews with school authorities (number unspecified) |
| Alexander et al, 2014 (21) | Kenya | Lower-middle | School staff | 62 Headteachers |
| Asimah et al, 2017 (22) | Ghana | Lower-middle | Girls in school*, guardians | 319 pupils (aged 10-19, with 229 females, 90 males across 15 schools), and 333 household heads (241 males, 92 females) |
| Averbach, et al, 2009 (23) | Zimbabwe | Lower-middle | Women | 43 women (aged 18-45) |
| Behera et al, 2015 (24) | India | Lower-middle | Adolescent girls** | 32 adolescent girls (female, aged 14-15) |
| Caruso et al, 2017 (25) | India | Lower-middle | Women | 69 women (for interviews, aged 18-75), and 46 women (for discussions, aged 18-70) |
| Caruso et al, 2014 (26) | Kenya | Lower-middle | Girls in school*, school staff | 36 students (female, aged 11-17, across 3 primary schools for focus groups), 6 students (selected from the focus group discussions, for in-depth interviews), 2 teachers (for in-depth interviews) |
| Chakravarthy et al, 2019  (27) *(Paper uses 3 studies – 1 available report and 2 unpublished documents)* | India | Lower-middle | Women & girls, Government  officials | Unspecified number of adolescent girls (aged 10-19) women (aged 20-49) and 20 government officials.  Breakdown of participants not specified. |
| Chinyama et al, 2019 (28) | Zambia | Lower-middle | Girls in school*, school staff, guardians | 64 students (aged 14-18, 48 female, 16 male, for 8 focus group discussions), 12 students (aged 14-18, female, for in-depth interviews), 7 teachers (for key informant interviews), (all across 6 schools), 7 guardians (for key informant interviews), and 11 leaders (both male and female (for key informant interviews) |
| Chothe et al, 2014 (29) | India | Lower-middle | Girls in school* | 381 students (female, aged 9-13) |
| Connolly and Sommer, 2013 (30) | Cambodia | Lower-middle | Adolescent girls**, school staff, guardians | 146 adolescent girls (female, aged 16-19, mix of in and out of school), and 15 parents/ teachers |
| Coswosk et al, 2019 (31) | Brazil | Upper-middle | Girls in school*, school staff | School principal and vice-principal, 39 students (female and male, aged 13-17) |
| Crichton et al, 2013 (32) | Kenya | Lower-middle | Adolescent girls** | 87 students (aged 12-17), 69 women, 5 teachers, 1 nurse |
| Crofts and Fisher, 2011 and Crofts and Fisher, 2012 (33, 34) | Uganda | Low | Girls in school*, school staff, business leaders | 134 students (female, aged 13-20, for participatory activities and FDGs), 9 business leaders, 12 school staff |
| Daniels, 2016 (35) | Cambodia | Lower-middle | Adolescent girls**, adolescent boys, women, men, school staff | 165 participants (for interviews), 181 participants (for focus group discussions), including girls, boys, mothers, fathers, and teachers. Breakdown of participants not specified. |

| Dhingra et al, 2009 (36) | India | Lower-middle | Adolescent girls** | 200 girls (aged 13-15) |
| --- | --- | --- | --- | --- |
| Dolan et al, 2014 (37) | Ghana | Lower-middle | Girls in school*, parents, school staff | 99 girls (age unspecified, for interviews), 136 girls (age unspecified, including dropouts, for focus group discussions), 246 parents, 12 school staff (for key informant interviews), 156 school staff (for focus group discussions) |
| Ellis et al, 2016 (38) | Philippines | Lower-middle | Girls in school* | 79 students (female, aged 11-18, across 3 schools in urban Manilla, and 10 rural schools) |
| Garikipati and Boudot, 2017 (39) | India | Lower-middle | Adolescent girls**and women | 150 women and adolescent girls (aged 15-49, from 3 slum locations) |
| Girod et al, 2017 (40) | Kenya | Lower-middle | Girls in school*, school staff | 51 students (approximately – number of students not explicitly stated, female, grades 6-8 (≈ aged 11-14),  across 6 different primary schools) and 6 Headteachers |
| Gultie et al, 2014 (41) | Ethiopia | Low | Girls in school* | 492 students (female, grades 9-12, aged 13-21+) |
| Hennegan et al, 2017 (42) | Uganda | Lower-middle | Girls in school* | 27 students (female, aged 12-17, across 8 schools) |
| Hennegan et al, 2016 (43) | Uganda | Lower-middle | Girls in school* | 205 students (female, aged 10-19, across 8 schools) |
| Karibu et al, 2019 (44) | Nigeria | Lower-middle | Adolescent girls** | 492 adolescent girls (aged 10-19, covering both those in and out of school) |
| Kohler et al, 2019 (45) | India, Uganda | Lower-middle | Women, and men  (inpatients and healthcare staff) | 50 Indian participants and 40 Ugandan participants (across 4 hospitals, for workshops, interviewees selected from this sample). Both samples included inpatients and staff. |
| Lahme et al, 2018 (46) | Zambia | Lower-middle | Girls in school* | 51 students (female, aged 13-20, across 3 schools) |
| Mason et al, 2013 (47) | Kenya | Lower-middle | Girls in school* | 120 Students (female, aged 14-16, cross 6 schools) |
| Miiro et al, 2018 (48) | Uganda | Lower-middle | Girls in school*, boys in school, school staff, Municipality officials, parents | 562 Students (352 female and 210 male, aged 13-18, across 4 schools), 11 teachers, 2 municipality officials (Ministry of Education and the Ministry of Health), 10 parents |
| Mohamed et al, 2018 (49) | Fiji, Papua  New  Guinea,  Solomon  Islands | Lower-middle | Women & girls, men, school staff, community members (including vendors, employers, health workers, community leaders and  vulnerable women) | 54 girls in school (aged 13-26), 43 adolescent girls  (aged 13-29), 118 women (aged 19-61), 51 men (aged 23-70), 8 school staff, and 34 community members |
| Muralidharan, 2019 (50) | India | Lower-middle | Women & girls | Up to 72 adolescent girls (aged 15-24), and their mothers (total of number of participants not stated) |
| Ndlovu and Bhala, 2016  (51) | Zimbabwe | Lower-middle | Women, NGOs,  Public Sector, Religious institutions | 40 women, 30 key informants (15 males and 5 females, including public sector departments, churches and NGOs) |
| Oche et al, 2012 (52) | Nigeria | Lower-middle | Adolescent girls** | 122 adolescent girls (aged 15-20, across 4 schools) |
| Parker et al, 2014 (53) | Uganda | Lower-middle, and Displacement Camp (in and out of displacement camps) | Girls in school*, women, school  staff, health workers | Up to 240 students (aged 9-20, across 14 schools), 8 senior/head teachers, 9 health workers, up to 75 women (across 4 villages), up to 450 women (across 13 IDP settings) (Total of number of participants not stated) |

| Rajagopal and Mathur, 2017 (54) | India | Lower-middle | Adolescent girls** | 270 adolescent girls (130 school-going, 140 nonschool-going, aged 10-20, across 5 schools) |
| --- | --- | --- | --- | --- |
| Rajaraman et al, 2013 (55) | India | Lower-middle | Women | 48 women |
| Ramathuba, 2015 (56) | South Africa | Upper-middle | Girls in school* | 273 students (female, aged 14-19, across 6 schools) |
| Rastogi et al, 2019 (57) | India | Lower-middle | Girls in school*, parents, school staff | 187 students (female, aged 13-15, across 4 schools), parents and school staff. Total of number of participants not stated. |
| Rheinländer et al, 2019  (58) | Ghana | Lower-middle | Girls in school*, school staff | 33 students (female, aged 14-23, across 2 schools), 4 school staff (female) |
| Rizvi and Ali, 2016 (59) | Pakistan | Lower-middle | Adolescent girls** | 20 adolescent girls (aged 13-19, non-school-going) |
| Schmitt et al, 2017 (60) | Lebanon, Myanmar | Displacement  Camp | Women & girls, humanitarian staff | 117 women (aged 19-49), 71 adolescent girls (aged 14-18, 32 for focus group discussions and 39 for participatory mapping), 17 emergency response staff |
| Scorgie et al, 2016 (61) | South Africa | Upper-middle | Women | 21 women (aged 18-35, 17 of these completed the photovoice segment, then 7 of these then completed interviews) |
| Shah et al, 2019 (62) | Gambia | Low | Girls in school*, mothers, school staff | 470 students (427 female - aged 11-21, 43 male – aged 15-21), 3 school staff, 5 mothers |
| Sivakami et al, 2019 (63) | India | Lower-Middle | Girls in school* | 2564 students (female, aged 12+ (average age 14), across 43 schools) |
| Sommer et al, 2015 (64) | Cambodia,  Ghana,  Ethiopia (This study also draws from a previous study from Tanzania –  Sommer, 2009, for comparison purposes, this study is detailed below) | Low and LowerMiddle | Adolescent girls**, school staff, parents, health staff | ≈ 450 adolescent girls (aged 16-19, both in and out of school, across the 3 countries), school staff, parents, health staff (total of number of participants not stated) |
| Sommer, 2009 (65) | Tanzania | Lower-Middle | Adolescent girls** | ≈ 140 adolescent girls (aged 16-19) (Total of number of participants not stated) |
| Tamiru et al, 2015 (66) | Ethiopia,  South  Sudan,  Tanzania,  Uganda,  Zimbabwe | Low and LowerMiddle | Girls in school*, boys in school,  school staff, community members | Total of number of participants not stated |
| Tegegne and Sisay, 2014  (67) | Ethiopia | Low | Adolescent girls**, school Staff | At least 595 students (female, aged 10-19), 5 adolescent girls (who had dropped out of school), 4 teachers (all female). Total of number of participants not stated. |
| Trinies et al, 2015 (68) | Mali | Low | Girls in school*, school Staff | 26 students (female, aged 12-17, across 8 schools),  14 school staff (4 female, 10 male, across 8 schools). |
| Umeora and Egwuatu, 2008 (69) | Nigeria | Lower-middle | Women | 1692 women (female, aged 17-56). |
| Visaria and Mishra, 2017  (70) | India | Lower-middle | Adolescent girls** | 585 adolescent girls (aged 12-19, split across the experiment area (406) and a control group (179), spanning rural and urban communities). Total number of participants not explicitly stated. |

| Wardell and Czerwinski, 2001 (71) | USA | High | Women | 33 women (aged 22-27, on active duty or reserve forces for the military) |
| --- | --- | --- | --- | --- |
| WaterAid Nepal, 2009  (72) | Nepal | Low | Girls in school* | 204 students (female, aged 12-20, across 4 schools) |
| Wilson et al, 2014 (73) | Kenya | Lower-middle | Girls in School* | 302 students (female, aged 4-4, across 10 schools) |
| Yeasmin et al, 2017 (74) | Bangladesh | Lower-middle | Women, men, waste emptiers | 43 women, 25 men, 14 children, 5 faecal sludge emptying operators, 4 waste bin emptiers, |

*‘Girls in school’ refers to studies that were specifically conducted in a school environment, **‘adolescent girls’ refers to participants either not in education, or studies that were set outside the school environment

Table 2: Codebook definitions and coding frequency.

|  | **Code** | **Definition** | | **Studies** |
| --- | --- | --- | --- | --- |
| **Practise**    **(**  **)**  **Deductive Codes** | Intentional  Disposal | Menstruators chose to engage in a certain disposal technique (e.g. throwing into a latrine, field, jungle, canal, or bin; flushing down a toilet, burying them; wrapping them in newspaper, plastic, or paper; or  leaving them on the toilet floor) | | 37 |
|  | Washing,  drying or reuse | Washing between uses to reuse materials; washing blood of materials for religious/cultural reasons before disposing; drying in the sun (on roofs/washing lines); or drying inside homes (open-air drying and hiding  whilst drying) | | 30 |
| **Reason / Behavioural Driver**    **(**  **)**  **Inductive Codes** | State of  Available  Facilities  *(40*    *Studies)* | Physical Infrastructure | Does the sanitation facility meet desired physical sanitation needs? | 36 |
|  |  | Social Perceptions | Does the sanitation facility meet desired social needs? | 29 |
|  | Knowledge    *(11*  *)*  *Studies* | Lack of knowledge | Menstruators have not been taught how to dispose / wash / dry materials | 11 |
|  | Menstrual Taboos and Social Stigma    *(36*  *)*  *studies* | Cultural Beliefs | General beliefs discouraging / encouraging certain methods of disposal | 19 |
|  |  | Embarrassment and Worry | Unpleasant emotions related to doing something considered by others to be wrong or shameful | 20 |
|  |  | Fear | Unpleasant emotion caused by the threat of danger, pain or other harmful consequences | 11 |

## 3.1 Reasoning behind behaviour

When investigating the reasoning behind menstruators’ choice to use certain disposal and washing practices, the predominant factor was the availability of appropriate WASH facilities (where

‘appropriateness’ was defined by users). Of the 54 studies, 40 mentioned that the reason for the disposal or washing practice directly related to the state of the facilities used to manage menstruation, 36 the physical needs of the individual, and 29 the social perceptions of the facility according to individuals. There were 11 instances of menstruators stating lack of knowledge as a reason that affected their behaviour, as they stated they were unsure what was supposed to be done after menstrual materials had been used. Menstrual stigma and taboos were stated in 36 papers as a reason influencing disposal and washing practises.

Where the physical state of WASH infrastructure was mentioned, it related to the quantity of available physically functional toilets/latrines (22, 25, 27, 31, 35, 38, 40, 53, 55, 56, 58, 60, 63, 64, 67), the design of toilets/latrines (31, 35, 38, 40, 43, 45, 48, 51, 53, 55, 60, 61, 64, 66), the quality and availability of running water in and around toilets or latrines (20, 30, 35, 37, 38, 41, 43, 45, 46, 51, 53, 57, 64, 65, 67, 68, 70, 71, 72), the availability of soap for washing (45, 47, 48, 53, 57, 66, 67) and the availability of a physically functional disposal mechanism and/or service for used material (20, 27, 30, 31, 32, 33, 34, 35, 39, 40, 41, 45, 51, 56, 57, 61, 63, 66, 67, 71, 72). Social perceptions of appropriate infrastructure were driven by the presence or absence of a private/safe space for managing menstruation (27, 32, 34, 35, 37, 38, 40, 41, 43, 45, 48, 50, 51, 54, 56, 60, 61, 64, 66, 67, 68, 72), the cleanliness and maintenance of the facility (25, 26, 27, 30, 34, 35, 38, 40, 45, 51, 54, 57, 58, 64, 65, 66, 68, 72), the time they had available to change, wash or dispose of materials when using facilities, (27, 63) and the availability of gender segregated toilets / latrines (60, 64, 67, 68).

In 11 of the studies girls explicitly stated that they had been given no, or limited~~,~~ advice regarding how to dispose of menstrual materials (21, 24, 27, 29, 35, 41, 48, 54, 58, 60, 61). However, menstruators also stated they chose their method of disposal to limit environmental harm (44), or not cause detrimental harm to infrastructure systems (61) (e.g. disposable pads blocking flush systems).

36 studies indicated that the choice of disposal or washing behaviour was driven by menstrual taboos and social stigma. For example, menstruators that used reusable materials often dried them discreetly to hide them from others, often drying their washed materials inside, sometimes in a hidden corner (36, 42, 43,

44, 53, 54, 62, 67, 68, 69, 70), under their clothes (50, 54, 56, 60), or hidden under other drying items (34, 49, 53, 72). There were instances of women drying menstrual materials outside (42, 44, 49, 53, 73) but only in cases where they believed that there was adequate separation of homes so that neighbours could not see. When using disposable materials, many menstruators wrapped their used menstrual materials in newspaper or polythene bags before disposal, so as to obscure their waste (24, 31, 38, 50, 54, 57, 59, 60, 61, 74). In addition, some cultures discouraged the use of open disposal, such as bins, due to fears of witchcraft and infertility (28, 29, 34, 39, 45, 51, 52, 61, 64, 66, 68, 69, 74), or due to beliefs it is a sin to throw (especially unwashed) materials into a bin (59).

# 4. Discussion

There are three main drivers of menstrual disposal and washing behaviour, and they can be independent or influenced by one another. They can be individual, for example, there may be no bin ~~in the~~ facility so a menstruator cannot dispose of materials; the menstruator may not have been taught how to dispose of used materials so does not know what to do, or the menstruator may have been taught that they must not incinerate materials lest evil spirits negatively affect their health. However, the reasons for disposal and washing menstrual materials can also be multi-faceted. In some instances, all three combine to influence behaviour: for example, a menstruator may not have been taught how to dispose of materials, feels uncomfortable openly discussing disposal options due to menstrual stigma, may not know what facilities are needed or available, or how to change facilities or practices to make them more appropriate.

## 4.1 Understanding the drivers

The state of WASH facilities was the predominant driver in 40 studies. This included the physical needs and social perceptions of menstruators, but often also incorporated menstrual taboos and social stigma, for example, just because facilities existed, and were technically ‘appropriate’, did not mean they were used. Crofts and Fisher noted that incinerators had been constructed in five of the 18 schools where interviews took place, so there was a physically functioning menstrual disposal option present, however the actual usage was low (34). The incinerators had been built on the opposite side of the school to the toilets, so students did not want to be seen entering the incinerator building, and even when disposal buckets were provided in toilets, there was no management in place to take waste from toilets to the incinerator (34). So, although there were physically appropriate disposal facilities, due to stigma around being seen holding or disposing of used materials, the disposal facility was not used. The theme of girls not wanting to be seen disposing or washing their used materials was driven by two emotions: being embarrassed and/or worried (20 studies), or fearing that they would be seen, and what would happen if they were seen (11 studies).

Embarrassment and worry were direct results of being seen disposing or washing menstrual materials, and often lead to hiding of materials, and secretive behaviours (25, 26, 27, 32, 33, 35, 38, 40, 49, 53, 54, 56,

57, 58, 59, 60, 61, 62, 68, 74) . This worry was usually related to having ones’ menstrual status exposed (meaning those around the menstruating individual are aware that they are currently experiencing their period). Embarrassment drove disposal and washing behaviours and methods that favoured privacy. This behaviour can be harmful, as if menstruators are choosing to hide materials when drying them (e.g. under beds or other clothes), they may not be properly dried, and it is possible to contract infections and skin irritation. Menstruators are often aware of the dangers of incomplete drying of reusable products (23, 34, 51), but feel they have no other alternative due to the stigma surrounding exposing menstrual status, an example of knowledge existing but stigma more strongly influencing a behaviour.

Beyond embarrassment, fear of being seen disposing or washing was a recurring theme (32, 33, 38, 46, 47, 50, 58, 60, 61, 64, 67). This related to favouring methods of disposal or washing that were deemed secretive or hidden in order to limit girls’ menstrual status being exposed (32, 58, 67). Within this theme, girls noted they wanted to hide their used materials so that animals, specifically dogs, couldn’t find their materials and expose their status (61, 64). Girls especially wanted to hide their status from males, specifically fathers (38, 47). This was in part due to fears of unwanted sexual advance (33), abuse and violence (50, 60) and being forced to drop out of school at menarche so as to get married (46). Other fears to use disposal methods stemmed from girls being afraid that having a period was a “punishment from God” so hid all materials so that their parents would not find out (33, p.523) or that if seen, they would risk “infertility” or “being cursed” (64, p.596).

Cultural knowledge also influenced the choices that drove disposal and washing behaviours. For example, incinerators are often used for menstrual waste (33), and although they may be technically appropriate, in some cultures there are negative connotations surrounding the burning of menstrual blood (62) or instances where menstrual blood is kept to be used for ritual purposes (22, 69). These feeling were highlighted in Karibu et al’s Nigerian study where “38.0% stated that they chose what they considered to be the best disposal method to ensure protection from metaphysical forces…[and]… 2.4% said they chose their method to avoid evil people” (44, pp.108-9).

When examining the language used in the papers, it became apparent that there is a bias in the way some disposal and washing methods, as well as menstrual materials themselves, are written about by the authors, most of whom are WASH researchers or practitioners. There has been a tendency to write about material use or disposal and washing behaviours with a view that some are superior to others, even where there is limited health or technical evidence to support this. Bias was highlighted in statements such as ‘*only* x % of interviewees use sanitary pads’ (emphasis added, (39, (p. 42), 66, (p.96), 67, (p.6), 70,

(p.102)). Although sometimes the bias was used in ways to highlight unhygienic practices, for example

“only a few used any kind of antiseptic soap or liquid [to wash their pads]” (24, p.515) it still singled out and shamed individuals rather than considering the myriad of factors contributing to their behaviours and the suitability of their washing and disposal practices to their personal circumstances. A similar bias occurs in Community Led Total Sanitation and some sanitation marketing programmes, where people are identified, and often shamed for their behaviours, regardless of their ability to change a situation, whether that be due to insufficient funds, lack of access to different options, or personal behavioural and cultural choices (75, 76).

It must be understood that there are many reasons for choosing specific menstrual materials and disposal and washing behaviours, and that writing in a style that judges choices may vilify individuals, reinforce harmful taboos, and not succeed in changing behaviours to those which may improve the wellbeing of those who menstruate. WASH practitioners who read such studies may develop their own biases against behaviours which are in fact appropriate to local contexts and low risk to menstruators’ health. For example, there has been a frequent bias in the WASH literature, and programming, against the use of ‘cloths’ or ‘rags’, often conflated as one. However, this does not consider that a cleaned and dried reusable pad or cloth may be just as hygienic and clean as a disposable pad, whilst a dirty rag poses obvious hygiene risks. In Chakravarty et al’s paper, this infiltrated bias had directly affected material use, with one menstruator stating, “here we use only pads, we now realise how unhygienic it is to use cloth” (27, p.147). There are several factors that contribute to menstrual material choice including, but not limited to, the preferences of and options for disposal and washing available to those who menstruate.

When programming for MHH and the associated WASH facilities, WASH professionals must consider not just preferences for menstrual material choice, technologies, and disposal and washing practices, but also the physical and social perceptions of menstruators, the availability and form of knowledge in the cultural context, and the menstrual taboos and social stigma that continue to impact on those who menstruate across the globe. The focus of MHH programming must be to improve the physical, mental, and social well-being of menstruators within their own contexts, not to promote specific WASH facilities or technologies preferred by implementers; considering the drivers of menstrual behaviour throughout the lifecycle of materials will assist in improved experiences for menstruators.

## 4.2 Limitations

This was a global review of published data. We originally aimed to highlight and understand the disposal and washing practices of those who menstruate around the world, with no geographical or economic limitations. However, of the 54 studies included in this review, only one was from a high-income economy and another three were from upper-middle income economies. This was surprising given the media coverage in recent years surrounding the ‘fatbergs’ negatively impacting the functionality of sewers in cities in higher income countries (77). Such fatbergs are attributed largely to the flushing of menstrual materials and wet wipes. Water utilities have put out calls to stop such behaviour (78), but, similar to the way users are often encouraged to change their menstrual material disposal behaviours in LMICs through the imparting of knowledge, these campaigns tend to assume that people flush these items because they do not know how harmful the practice is. Very limited work has investigated the behavioural drivers of menstrual material disposal in higher income economies.

Thus, although this study set out to reflect worldwide practices, due to the lack of data from higher income economies, this study is not representative of global practices. There were parallels within the small amount of higher/upper-middle income data that showed similar themes and reasoning behind drivers of behaviour to LMICs, but due to the low proportion of these papers, we cannot determine with certainty whether these behaviours are universal. We join with Alda-Vidal et al. (14) in calling for further social science research on the clear gap of higher income economy data, and suggest it will be of particular interest in areas where fatbergs are impacting on WASH systems.

# 5. Conclusion

This review demonstrates the complex nature of washing and disposal behaviours related to menstruation. Behaviours are often not solely reliant on one factor, but several interrelated considerations. It is the first review that has aimed at understanding why people choose to engage in various menstrual material disposal and washing practices.

WASH professionals and other implementers of menstrual disposal and washing facilities need to ensure that disposal and washing options are appropriate for their context. Even when facilities are installed and accessible, if considered inappropriate they will not be used (34, 45, 51). In addition, educational policy needs to allow for the teaching of menstruation in a scientific, judgement-free zone, where girls feel comfortable to learn without the fear of embarrassment. Many girls, and teachers in some instances, were highlighted as not having sufficient knowledge about menstruation and menstrual management, with a specific emphasis on missing disposal information (21, 24, 29). By creating a safe space to facilitate discussion, girls will be able to learn more about this taboo topic.

Menstrual material disposal and washing is an area that is poorly understood globally, and as the first systematic review compiling these behaviours, this paper begins to provide clarity in an under-researched area. By exploring the drivers of disposal and washing behaviours, the interfaces between facilities, knowledge and taboos have been demonstrated. It is clearly important to integrate these aspects into the planning and provision of designing infrastructure systems that directly connect to MHH in order to provide accessible and appropriate facilities for all.

# Acknowledgements

The authors would like to thank Professor Jamie Bartram, Dr Celia Way, Dr Fiona Zakaria, Ms Mariam Zaqout and Mr Mofwe Kapulu for comments on an earlier version of this manuscript. The lead author of this paper is supported by the Engineering and Physical Sciences Research Council Grant number

EP/S022066/1.

References:

1. Sommer M, Sahin M. Advancing the global agenda for menstrual hygiene management for schoolgirls. American Journal of Public Health. 2013;103(9):1556-9.
2. Hennegan J, Montgomery P. Do menstrual hygiene management interventions improve education and psychosocial outcomes for women and girls in low and middle income countries? A systematic review. PLoS ONE. 2016;11(2).
3. Shah SP, Nair R, Shah PP, Modi DK, Desai SA, Desai L. Improving quality of life with new menstrual hygiene practices among adolescent tribal girls in rural Gujarat, India. Reproductive Health Matters. 2013;21(41):205-13.
4. APS Group Scotland. Access to Sanitary Products Aberdeen Pilot: Evaluation Report. Edinburgh, UK: The Scottish Government; 2018.
5. Kansiime C, Hytti L, Nalugya R, Nakuya K, Namirembe P, Nakalema S, et al. Menstrual health intervention and school attendance in Uganda (MENISCUS-2): a pilot intervention study. BMJ Open. 2020;10(2):e031182.
6. Mattsson J, Hedström A, Ashley RM, Viklander M. Impacts and managerial implications for sewer systems due to recent changes to inputs in domestic wastewater – A review. Journal of Environmental Management. 2015;161:188-97.
7. Sumpter C, Torondel B. A Systematic Review of the Health and Social Effects of Menstrual Hygiene Management. PLoS ONE. 2013;8(4).
8. International Standards Organisation. Non-sewered sanitation systems — Prefabricated integrated treatment units — General safety and performance requirements for design and testing. ISO 30500:2018. Switzerland: Geneva.: International Standards Organisation,; 2018.
9. Wynne B. Knowledges in Context. Science, Technology, & Human Values. 1991;16(1):111-21.
10. Coffey D, Gupta A, Hathi P, Khurana N, Spears D, Srivastav N, et al. Revealed preference for open defecation. Economic & Political Weekly. 2014;49(38):43-55.
11. Curtis VA, Danquah LO, Aunger RV. Planned, motivated and habitual hygiene behaviour: an eleven country review. Health Education Research. 2009;24(4):655-73.
12. Elledge MF, Muralidharan A, Parker A, Ravndal KT, Siddiqui M, Toolaram AP, et al. Menstrual Hygiene Management and Waste Disposal in Low and Middle Income Countries - A Review of the Literature. International Journal of Environmental Research and Public Health. 2018;15(11):1-16.
13. Kaur R, Kaur K, Kaur R. Menstrual Hygiene, Management, and Waste Disposal: Practices and Challenges Faced by Girls/Women of Developing Countries. Journal of Environmental and Public Health. 2018;2018.
14. Alda Vidal C, Browne A, Hooloha, C. Unflushables”: Establishing a global agenda for action on

everyday practices associated with sewer blockages, water quality, and plastic pollution. Wiley Interdisciplinary Reviews: Water 2020;7(7):e1452.

15. Moher D, Liberati A, Tetzlaff J, Altman DG, The Prisma Group. Preferred Reporting Items for Systematic Reviews and Meta-Analyses: The PRISMA Statement. PLOS Medicine. 2009;6(7):e1000097.
16. Preferred Reporting Items for Systematic Reviews and Meta-Analyses. PRISMA 2009 Checklist 2009 [Available from: [http://www.prisma-statement.org/PRISMAStatement/Checklist.aspx.](http://www.prisma-statement.org/PRISMAStatement/Checklist.aspx)
17. Rees R, Oliver K, Woodman J, Thomas J. Children’s views about obesity, body size, shape and weight. University of London: Institute of Education; 2009. Available from:

[http://eppi.ioe.ac.uk/cms/Portals/0/Obesity%20Views%20Children%20R2009Rees.pdf?ver=2010-12-22121209-040.](http://eppi.ioe.ac.uk/cms/Portals/0/Obesity%20Views%20Children%20R2009Rees.pdf?ver=2010-12-22-121209-040)

18. . NVivo qualitative data analysis software: QSR International Pty Ltd; Version 12, 2018.
19. The World Bank. World Bank Country and Lending Groups. 2020 [18th November 2020]. Available from: [https://datahelpdesk.worldbank.org/knowledgebase/articles/906519-world-bank-countryand-lending-groups.](https://datahelpdesk.worldbank.org/knowledgebase/articles/906519-world-bank-country-and-lending-groups)
20. Abera Y. Menarche, Menstruation related Problems and Practices among Adolescent High School Girls in Addis Ababa, 2003/04: Addis Ababa University; 2004.
21. Alexander KT, Oduor C, Nyothach E, Laserson KF, Amek N, Eleveld A, et al. Water, sanitation and hygiene conditions in kenyan rural schools: Are schools meeting the needs of menstruating girls? Water (Switzerland). 2014;6(5):1453-66.
22. Asimah SA, Diabene PY, Wellington SNL. Menstrual hygiene management in Ghana:

understanding the socio-cultural, economic, political factors, challenges and opportunities. Local action with international cooperation to improve and sustain water, sanitation and hygiene (WASH) services; Loughborough, UK. Loughborough: Loughborough University; 2017.

23. Averbach S, Sahin-Hodoglugil N, Musara P, Chipato T, van der Straten A. Duet® for menstrual protection: a feasibility study in Zimbabwe. Contraception. 2009;79(6):463-8.
24. Behera D, Sivakami M, Behera MR. Menarche and Menstruation in Rural Adolescent Girls in Maharashtra, India. Journal of Health Management. 2015;17(4):510-9.
25. Caruso BA, Clasen TF, Hadley C, Yount KM, Haardörfer R, Rout M, et al. Understanding and defining sanitation insecurity: Women's gendered experiences of urination, defecation and menstruation in rural Odisha, India. BMJ Global Health. 2017;2(4).
26. Caruso BA, Dreibelbis R, Ogutu EA, Rheingans R. If you build it will they come? Factors influencing rural primary pupils' urination and defecation practices at school in western Kenya. Journal of Water Sanitation and Hygiene for Development. 2014;4(4):642-53.
27. Chakravarthy V, Rajagopal S, Joshi B. Does Menstrual Hygiene Management in Urban Slums Need a Different Lens? Challenges Faced by Women and Girls in Jaipur and Delhi. Indian Journal of Gender Studies. 2019;26(1-2):138-59.
28. Chinyama J, Chipungu J, Rudd C, Mwale M, Verstraete L, Sikamo C, et al. Menstrual hygiene management in rural schools of Zambia: A descriptive study of knowledge, experiences and challenges faced by schoolgirls. BMC Public Health. 2019;19(1).
29. Chothe V, Khubchandani J, Seabert D, Asalkar M, Rakshe S, Firke A, et al. Students' Perceptions and Doubts About Menstruation in Developing Countries: A Case Study From India. Health Promotion Practice. 2014;15(3):319-26.
30. Connolly S, Sommer M. Cambodian girls' recommendations for facilitating menstrual hygiene management in school. Journal of Water Sanitation and Hygiene for Development. 2013;3(4):612-22.
31. Coswosk ÉD, Neves-Silva P, Modena CM, Heller L. Having a toilet is not enough: The limitations in fulfilling the human rights to water and sanitation in a municipal school in Bahia, Brazil. BMC Public Health. 2019;19(1).
32. Crichton J, Okal J, Kabiru CW, Zulu EM. Emotional and Psychosocial Aspects of Menstrual Poverty in Resource-Poor Settings: A Qualitative Study of the Experiences of Adolescent Girls in an Informal Settlement in Nairobi. Health Care for Women International. 2013;34(10):891-916.
33. Crofts T, J., Fisher J, editors. Schoolgirls' experiences of managing menstrual hygiene in Uganda. 2011 35th WEDC International Conference - The Future of Water, Sanitation and Hygiene in LowIncome Countries: Innovation, Adaptation and Engagement in a Changing World; 2011; Loughborough.
34. Crofts T, Fisher J. Menstrual hygiene in Ugandan schools: An investigation of low-cost sanitary pads. Journal of Water Sanitation and Hygiene for Development. 2012;2(1):50-8.
35. Daniels GJ. Investigating Fear, Shyness, And Discomfort Related To Menstrual Hygiene Management In Rural Cambodia: Yale University; 2016.
36. Dhingra R, Kumar A, Kour M. Knowledge and practices related to menstruation among Tribal (Gujjar) adolescent girls. Studies on Ethno-Medicine. 2009;3(1):43-8.
37. Dolan CS, Ryus CR, Dopson S, Montgomery P, Scott L. A blind spot in girls' education:

Menarche and its webs of exclusion in Ghana. Journal of International Development. 2014;26(5):643-57.

38. Ellis A, Haver J, Villasenor J, Parawan A, Venkatesh M, Freeman MC, et al. WASH challenges to girls' menstrual hygiene management in Metro Manila, Masbate, and South Central Mindanao, Philippines. Waterlines. 2016;35(3):306-23.
39. Garikipati S, Boudot C. To Pad or Not to Pad: Towards Better Sanitary Care for Women in Indian Slums. Journal of International Development. 2017;29(1):32-51.
40. Girod C, Ellis A, Andes KL, Freeman MC, Caruso BA. Physical, Social, and Political Inequities Constraining Girls’ Menstrual Management at Schools in Informal Settlements of Nairobi, Kenya. Journal of Urban Health. 2017;94(6):835-46.
41. Gultie T, Hailu D, Workineh Y. Age of menarche and knowledge about menstrual hygiene management among adolescent school girls in amhara province, Ethiopia: Implication to health care workers & school teachers. PLoS ONE. 2014;9(9).
42. Hennegan J, Dolan C, Steinfield L, Montgomery P. A qualitative understanding of the effects of reusable sanitary pads and puberty education: Implications for future research and practice. Reproductive Health. 2017;14(1).
43. Hennegan J, Dolan C, Wu M, Scott L, Montgomery P. Measuring the prevalence and impact of poor menstrual hygiene management: A quantitative survey of schoolgirls in rural Uganda. BMJ Open. 2016;6(12).
44. Karibu K, Salami JC, Azeez M, Azeez A. Onset of Menarche and Adolescent Menstrual Hygiene Practices in Semi-Urban Ibadan Community, Nigeria. Women's Reproductive Health. 2019;6(2):102-17.
45. Kohler P, Renggli S, Lüthi C. WASH and gender in health care facilities: The uncharted territory. Health Care for Women International. 2019;40(1):3-12.
46. Lahme AM, Stern R, Cooper D. Factors impacting on menstrual hygiene and their implications for health promotion. Global Health Promotion. 2018;25(1):54-62.
47. Mason L, Nyothach E, Alexander K, Odhiambo FO, Eleveld A, Vulule J, et al. 'We keep it secret so no one should know' - A qualitative study to explore young schoolgirls attitudes and experiences with menstruation in rural Western Kenya. PLoS ONE. 2013;8(11).
48. Miiro G, Rutakumwa R, Nakiyingi-Miiro J, Nakuya K, Musoke S, Namakula J, et al. Menstrual health and school absenteeism among adolescent girls in Uganda (MENISCUS): A feasibility study. BMC Women's Health. 2018;18(1).
49. Mohamed Y, Durrant K, Huggett C, Davis J, Macintyre A, Menu S, et al. A qualitative exploration of menstruation-related restrictive practices in Fiji, Solomon Islands and Papua New Guinea. PLoS ONE. 2018;13(12).
50. Muralidharan A. Constrained Choices? Menstrual Health and Hygiene Needs Among Adolescents in Mumbai Slums. Indian Journal of Gender Studies. 2019;26(1-2):12-39.
51. Ndlovu E, Bhala E. Menstrual hygiene - A salient hazard in rural schools: A case of Masvingo district of Zimbabwe. Jamba: Journal of Disaster Risk Studies. 2016;8(2):1-8.
52. Oche MO, Umar AS, Gana GJ, Ango JT. Menstrual health: The unmet needs of adolescent girls' in Sokoto, Nigeria. Scientific Research and Essays. 2012;7(3):410-8.
53. Parker AH, Smith JA, Verdemato T, Cooke J, Webster J, Carter RC. Menstrual management: A neglected aspect of hygiene interventions. Disaster Prevention and Management. 2014;23(4):437-54.
54. Rajagopal S, Mathur K. ‘Breaking the silence around menstruation’: experiences of adolescent girls in an urban setting in India. Gender and Development. 2017;25(2):303-17.
55. Rajaraman D, Travasso SM, Heymann SJ. A qualitative study of access to sanitation amongst low-income working women in Bangalore, India. Journal of Water Sanitation and Hygiene for Development. 2013;3(3):432-40.
56. Ramathuba DU. Menstrual knowledge and practices of female adolescents in Vhembe district, Limpopo Province, South Africa. Curationis. 2015;38(1).
57. Rastogi S, Khanna A, Mathur P. Uncovering the challenges to menstrual health: Knowledge, attitudes and practices of adolescent girls in government schools of Delhi. Health Education Journal. 2019;78(7):839-50.
58. Rheinländer T, Gyapong M, Akpakli DE, Konradsen F. Secrets, shame and discipline: School girls' experiences of sanitation and menstrual hygiene management in a peri-urban community in Ghana. Health Care for Women International. 2019;40(1):13-32.
59. Rizvi N, Ali TS. Misconceptions and Mismanagement of Menstruation among Adolescents Girls who do not attend School in Pakistan. Journal of Asian Midwives. 2016;3(1):46–62.
60. Schmitt ML, Clatworthy D, Ratnayake R, Klaesener-Metzner N, Roesch E, Wheeler E, et al. Understanding the menstrual hygiene management challenges facing displaced girls and women: Findings from qualitative assessments in Myanmar and Lebanon. Conflict and Health. 2017;11(1).
61. Scorgie F, Foster J, Stadler J, Phiri T, Hoppenjans L, Rees H, et al. “Bitten By Shyness”: Menstrual Hygiene Management, Sanitation, and the Quest for Privacy in South Africa. Medical Anthropology: Cross Cultural Studies in Health and Illness. 2016;35(2):161-76.
62. Shah V, Nabwera HM, Sosseh F, Jallow Y, Comma E, Keita O, et al. A rite of passage: A mixed methodology study about knowledge, perceptions and practices of menstrual hygiene management in rural Gambia. BMC Public Health. 2019;19(1).
63. Sivakami M, van Eijk AM, Thakur H, Kakade N, Patil C, Shinde S, et al. Effect of menstruation on girls and their schooling, and facilitators of menstrual hygiene management in schools: Surveys in government schools in three states in India, 2015. Journal of Global Health. 2019;9(1).
64. Sommer M, Ackatia-Armah N, Connolly S, Smiles D. A comparison of the menstruation and education experiences of girls in Tanzania, Ghana, Cambodia and Ethiopia. Compare. 2015;45(4):589609.
65. Sommer M. Ideologies of sexuality, menstruation and risk: girls' experiences of puberty and schooling in northern Tanzania. Culture, Health & Sexuality. 2009;11(4):383-98.
66. Tamiru S, Mamo K, Acidria P, Mushi R, Ali CS, Ndebele L. Towards a sustainable solution for school menstrual hygiene management: Cases of Ethiopia, Uganda, South-Sudan, Tanzania, and Zimbabwe. Waterlines. 2015;34(1):92-102.
67. Tegegne TK, Sisay MM. Menstrual hygiene management and school absenteeism among female adolescent students in Northeast Ethiopia. BMC Public Health. 2014;14(1).
68. Trinies V, Caruso BA, Sogoré A, Toubkiss J, Freeman MC. Uncovering the challenges to menstrual hygiene management in schools in Mali. Waterlines. 2015;34(1):31-40.
69. Umeora OU, Egwuatu VE. Menstruation in rural Igbo women of south east Nigeria: attitudes, beliefs and practices. African journal of reproductive health. 2008;12(1):109-15.
70. Visaria L, Mishra RN. Health Training Programme for Adolescent Girls: Some Lessons from India’s NGO Initiative. Journal of Health Management. 2017;19(1):97-108.
71. Wardell DW, Czerwinski B. A military challenge to managing feminine and personal hygiene. Journal of the American Academy of Nurse Practitioners. 2001;13(4):187-93.
72. WaterAid Nepal. Is Menstrual Hygiene and Management an Issue for Adolescent School Girls? A Comparative Study of Four Schools in Different Settings of Nepal. Kathmandu: WaterAid Nepal; 2009.
73. Wilson E, Reeve J, Pitt A. Education. Period. Developing an acceptable and replicable menstrual hygiene intervention. Development in Practice. 2014;24(1):63-80.
74. Yeasmin F, Luby SP, Saxton RE, Nizame FA, Alam M, Dutta NC, et al. Piloting a low-cost hardware intervention to reduce improper disposal of solid waste in communal toilets in low-income settlements in Dhaka, Bangladesh. BMC Public Health. 2017;17:1-11.
75. Bartram J, Charles K, Evans B, O'Hanlon L, Pedley S. Commentary on community-led total sanitation and human rights: should the right to community-wide health be won at the cost of individual rights? Journal of Water and Health. 2012;10(4):499-503.
76. Barrington DJ, Sridharan S, Shields KF, Saunders SG, Souter RT, Bartram J. Sanitation marketing: A systematic review and theoretical critique using the capability approach. Social Science & Medicine. 2017;194:128-34.
77. Michael M. London’s fatbergs and affective infrastructuring. Social Studies of Science. 2020;50(3):377-97.
78. ABC. Wet wipes, tampons, condoms targeted in new fatberg-fighting campaign. 2019 [18 November 2020]. Available from: [https://www.abc.net.au/radio/programs/pm/wet-wipes-tamponscondoms-targeted-fatberg-fighting-campaign/11339770.](https://www.abc.net.au/radio/programs/pm/wet-wipes-tampons-condoms-targeted-fatberg-fighting-campaign/11339770)

[Click here to access/download;Figure;Figure 1 - Inclusion and Exclusion Criteria - HJR DJB.docx](https://www.editorialmanager.com/pone/download.aspx?id=28182583&guid=6c896734-e8c9-44e8-938d-64aa2ab8be62&scheme=1)

Records identified through database searching and

existing reviews (n = 17,302)

Records after duplicates removed

(

n

= 11,888)

Titles and abstracts

screened

= 11,888)

n

(

Records excluded as per criteria 1

= 11,260)

n

(

Full records

assessed for eligibility

(

n

= 628)

Records excluded as per criteria 2

(

n

= 564)

Records

included in qualitative synthesis and analysis

(

n =

55

, describing 54 studies

)

[Click here to access/download](https://www.editorialmanager.com/pone/download.aspx?id=28182573&guid=d86fa40c-39c7-48f7-9966-a5731f1f3f1a&scheme=1)

[Supporting Information](https://www.editorialmanager.com/pone/download.aspx?id=28182573&guid=d86fa40c-39c7-48f7-9966-a5731f1f3f1a&scheme=1)

[Supplementary Material 1 - Final Included Studies .docx](https://www.editorialmanager.com/pone/download.aspx?id=28182573&guid=d86fa40c-39c7-48f7-9966-a5731f1f3f1a&scheme=1)

[Click here to access/download](https://www.editorialmanager.com/pone/download.aspx?id=28182574&guid=d353bbfa-66a2-48b4-a851-00e239a018a5&scheme=1)

[Supporting Information](https://www.editorialmanager.com/pone/download.aspx?id=28182574&guid=d353bbfa-66a2-48b4-a851-00e239a018a5&scheme=1)

[Supplementary Material 2 - Final Quality Appraisal.docx](https://www.editorialmanager.com/pone/download.aspx?id=28182574&guid=d353bbfa-66a2-48b4-a851-00e239a018a5&scheme=1)

[Click here to access/download](https://www.editorialmanager.com/pone/download.aspx?id=28182575&guid=bd1925fc-0cf3-4458-89e7-2d8ba384770a&scheme=1)

[Supporting Information](https://www.editorialmanager.com/pone/download.aspx?id=28182575&guid=bd1925fc-0cf3-4458-89e7-2d8ba384770a&scheme=1)

[Supplementary Material 3 - Final Driver Examples.docx](https://www.editorialmanager.com/pone/download.aspx?id=28182575&guid=bd1925fc-0cf3-4458-89e7-2d8ba384770a&scheme=1)

PRISMA Checklist

[Click here to access/download](https://www.editorialmanager.com/pone/download.aspx?id=28251295&guid=dd604295-cb46-4939-876f-5e633b289585&scheme=1)

[Supporting Information](https://www.editorialmanager.com/pone/download.aspx?id=28251295&guid=dd604295-cb46-4939-876f-5e633b289585&scheme=1)

[PRISMA Checklist - HJR DJB final.docx](https://www.editorialmanager.com/pone/download.aspx?id=28251295&guid=dd604295-cb46-4939-876f-5e633b289585&scheme=1)
