# Supplementary material for: Drivers of menstrual material disposal and washing practices: A systematic review
Source: PLoS One. 2021 Dec 3;16(12):e0260472. doi: 10.1371/journal.pone.0260472 (PMC8641861; doi:10.1371/journal.pone.0260472)
Supplement: Attachment — Submitted filename: S6_Response_to_Reviewers.docx [file pone.0260472.s006.docx]

Response to Reviewers

*RE: PONE-D-21-01105, September, 2021*

Additional Requirements:

| 1 | Please ensure that your manuscript meets PLOS ONE's style requirements, including those for file naming | File names have been adjusted accordingly and formatting has been adjusted to ensure style compliance. |
| --- | --- | --- |
|  |  |  |
| 2 | Please review your reference list to ensure that it is complete and correct. | No references have been retracted since initial submission.  27 new references have been added as per the updated systematic review. |
| 3 | Please confirm that you have included all items recommended in the PRISMA checklist including:   - The full electronic search strategy used to identify studies with all search terms and limits for at least one database. - An updated search and analysis that includes studies published since June 2019 | The search strategy is now included as Table 1.  An updated search was performed that identified 2,310 additional papers (after duplicates were removed) in June 2021, this resulted in an additional 27 references being included. Analysis of the new references did not change the findings of this study. |
|  |  |  |
| 4 | In your Data Availability statement, you have not specified where the minimal data set underlying the results described in your manuscript can be found. | This manuscript utilised secondary data in the form of published, peer-reviewed journal articles and theses. All of these articles are available online, and DOIs are provided in the reference list where possible. |
| 5 | Please include captions for your Supporting Information files at the end of your manuscript, and update any in-text citations to match accordingly. | Captions have been added, a new table has been included and existing tables have been updated accordingly. |

Reviewer Comments:

|  | Reviewer #1: | The manuscript is well written. But certain facts need to be cleared, which is mentioned in the corrected manuscript. The alignment of references could be checked. New references could be further added. Certain facts could be authenticated. | Alignment of all references has been addressed.  Additional references have been added where requested. |
| --- | --- | --- | --- |
|  |  |  |  |
|  | Reviewer #2: | Review article is well written. References to be rechecked and written as per journals instruction.  References should be changed as per instruction of the journal. Sufficient data enclosed and satisfactory | References have been shortened to use abbreviated titles, and DOIs have been included where possible. |
